# Iron(II) Complexes of 4-(Alkyldisulfanyl)-2,6-di(pyrazolyl)pyridine Derivatives. Correlation of Spin-Crossover Cooperativity with Molecular Structure Following Single-Crystal-to-Single-Crystal Desolvation

**DOI:** 10.1021/acs.cgd.2c00005

**Published:** 2022-02-04

**Authors:** Rafal Kulmaczewski, Laurence J. Kershaw Cook, Christopher M. Pask, Oscar Cespedes, Malcolm A. Halcrow

**Affiliations:** † School of Chemistry, University of Leeds, Woodhouse Lane, Leeds LS2 9JT, U.K.; ‡ School of Physics and Astronomy, University of Leeds, E. C. Stoner Building, Leeds LS2 9JT, U.K.

## Abstract

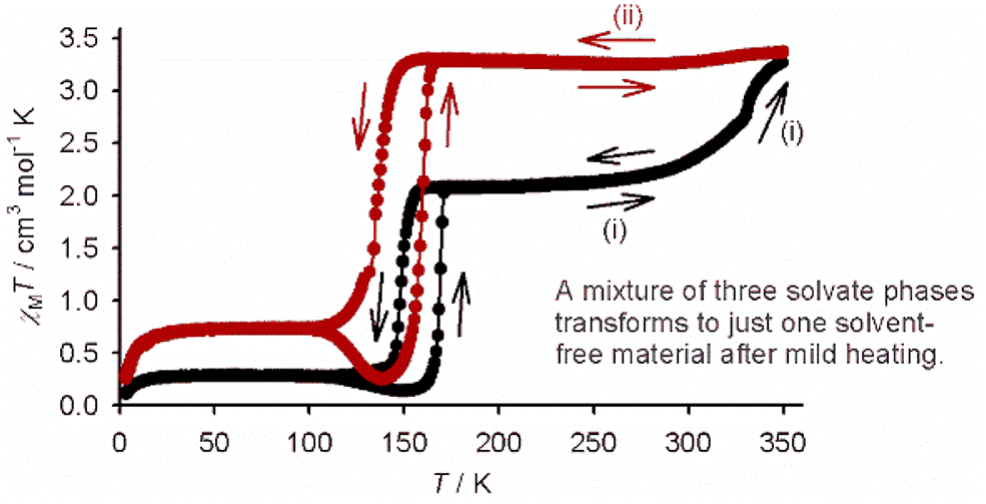

The complex salts [Fe(*L*^1^)_2_]X_2_ (**1X**_**2**_; *L*^1^ = 4-(isopropyldisulfanyl)-2,6-bis(pyrazolyl)pyridine; X^–^ = BF_4_^–^, ClO_4_^–^) form solvated crystals from common organic solvents. Crystals of **1X**_**2**_·Me_2_CO show abrupt spin transitions near 160 K, with up to 22 K thermal hysteresis. **1X**_**2**_·Me_2_CO cocrystallizes with other, less cooperative acetone solvates, which all transform into the same solvent-free materials **1X**_**2**_·sf upon exposure to air, or mild heating. Conversion of **1X**_**2**_·Me_2_CO to **1X**_**2**_·sf proceeds in a single-crystal to single-crystal fashion. **1X**_**2**_·sf are not isomorphous with the acetone solvates, and exhibit abrupt spin transitions at low temperature with hysteresis loops of 30–38 K (X^–^ = BF_4_^–^) and 10–20 K (X^–^ = ClO_4_^–^), depending on the measurement method. Interestingly, the desolvation has an opposite effect on the SCO temperature and hysteresis in the two salts. The hysteretic spin transitions in **1X**_**2**_·Me_2_CO and **1X**_**2**_·sf do not involve a crystallographic phase change but are accompanied by a significant rearrangement of the metal coordination sphere. Other solvates **1X**_**2**_·MeNO_2_, **1X**_**2**_·MeCN, and **1X**_**2**_·H_2_O are mostly isomorphous with each other and show more gradual spin-crossover equilibria near room temperature. All three of these lattice types have similar unit cell dimensions and contain cations associated into chains through pairwise, intermolecular S···π interactions. Polycrystalline [Fe(*L*^2^)_2_][BF_4_]_2_·MeNO_2_ (**2[BF**_**4**_**]**_**2**_·MeNO_2_; *L*^2^ = 4-(methyldisulfanyl)-2,6-bis(pyrazolyl)pyridine) shows an abrupt spin transition just above room temperature, with an unsymmetrical and structured hysteresis loop, whose main features are reversible upon repeated thermal scanning.

## Abstract 2

[Fe(*L*^1^)_2_]X_2_ (**1X**_**2**_; *L*^1^ = 4-(isopropyldisulfanyl)-2,6-bis(pyrazolyl)pyridine, X^−^ = BF_4_^−^, ClO_4_^−^) form mixtures of solvated phases from acetone/diethyl ether, which all transform to one solvent-free material **1X**_**2**_·sf upon mild heating. The conversion of **1X**_**2**_·Me_2_CO to **1X**_**2**_·sf is a single-crystal to single-crystal reaction. **1X**_**2**_·sf exhibit abrupt spin transitions with up to 38 K thermal hysteresis, accompanied by a significant angular rearrangement of the metal coordination geometry.

## Introduction

Crystal engineering of metal/organic spin-crossover (SCO) materials^1−7^ involves the interplay between the individual molecular switching centers and their surrounding lattice.^8^ The cooperativity of spin crossover reflects the structural changes occurring during the transition. That is, greater structural changes between the high-spin and low-spin forms lead to abrupt and/or hysteretic spin transitions and *vice versa*.^9^ SCO materials that are isomorphous or exhibit variations of the same packing motif are particularly helpful in allowing small differences between materials to be correlated with their switching function within the same lattice environment.^10−20^

Cooperative spin transitions often involve a crystallographic phase change,^21,22^ but wide hysteresis can arise without a phase change if the complex undergoes a large, anisotropic structural rearrangement between its spin states.^23−26^ However, to complicate matters, SCO may not occur if the structural difference between the spin states is too great or if the crystal is too densely packed.^9,27,28^ Both scenarios increase the activation energy of SCO so that it becomes quenched on kinetic grounds, even where a compound exhibits SCO under other conditions, such as in solution.^29^ Cooperative SCO requires a balanced combination of structural factors that are not too large, but not too small.

**Chart 1:**
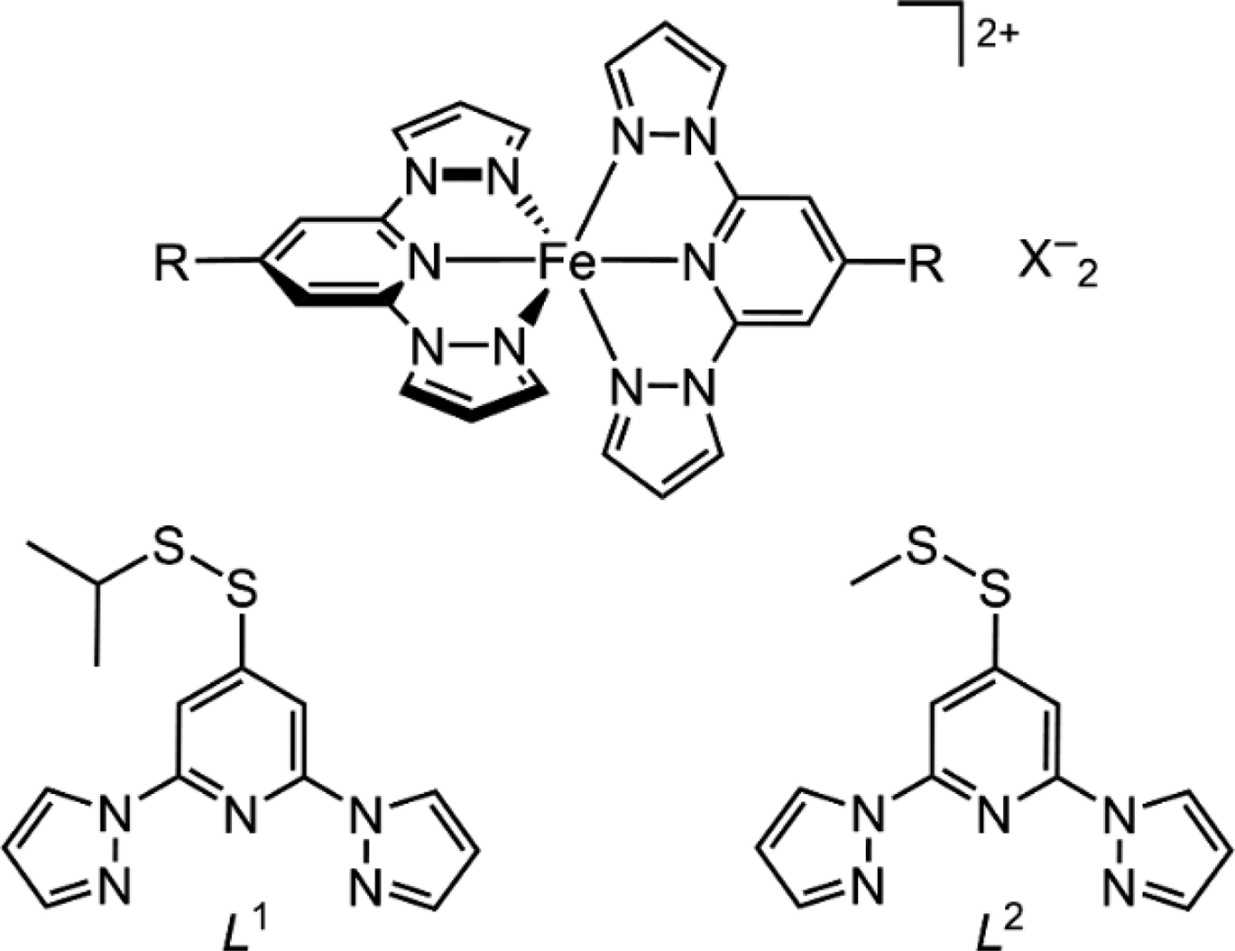
(Top) Structure of [Fe(bpp^R^)_2_]^2+^ (the Parent Complex [Fe(bpp)_2_]^2+^ Has R = H) and (Bottom) the Two New bpp^R^ Derivatives Described in This Work

Derivatives of [Fe(bpp)_2_]^2+^ (bpp = 2,6-bis(pyrazol-1-yl)pyridine; Chart 1) can be prepared with a variety of pyridyl and/or pyrazolyl substituents, which often exhibit SCO at accessible temperatures.^27,28,30,31^ The library of [Fe(bpp^R^)_2_]X_2_ (X^–^ = a monovalent anion) compounds is now large enough to allow structure–function correlations to be derived.^32,33^ Iron complexes of 4-alkylsulfanyl-2,6-bis(pyrazol-1-yl)pyridine ligands (bpp^R^; R = SMe, S*i*Pr, S*t*Bu) have been particularly useful.^19,34−38^ For example, solvate crystals of formula [Fe(bpp^S*i*Pr^)_2_]X_2_·solv (X^–^ = BF_4_^–^, ClO_4_^–^; solv = MeCN, EtCN, MeNO_2_, Me_2_CO, H_2_O, sf (solvent-free)) are all isomorphous in both spin states and can be interconverted by single-crystal to single-crystal solvent exchange. These exhibit a variety of spin-state behaviors that correlate with the shape of the lattice solvent molecule.^19,36,37^

The bpp^SMe^ and bpp^S*i*Pr^ ligands in these studies were synthesized by the alkylation of 4-mercapto-2,6-bis(pyrazol-1-yl)pyridine (bpp^SH^) with the appropriate iodoalkane.^34,36,39^ Since the product mixtures from these reactions contained lower yields of the corresponding 4-alkyldisulfanyl-substituted byproducts, we decided to investigate the iron complex chemistry of those ligands as well. We report here a family of SCO-active solvate crystals [Fe(*L*^1^)_2_]X_2_ (**1X**_**2**_; *L*^1^ = 4-isopropyldisulfanyl-2,6-bis(pyrazol-1-yl)pyridine; X^–^ = BF_4_^–^, ClO_4_^–^). Many of these solvates show clear structural similarities, which can be correlated with their SCO characteristics. Moreover, annealing some solvates causes single-crystal to single-crystal conversion to a solvent-free phase,^40,41^ showing a hysteretic spin transition that we have fully structurally characterized. A solvate of [Fe(*L*^2^)_2_][BF_4_]_2_ (**2[BF**_**4**_**]**_**2**_; *L*^2^ = 4-methyldisulfanyl-2,6-bis(pyrazol-1-yl)pyridine) showing an unusual asymmetric spin-transition profile is also briefly presented.

## Experimental Section

The synthetic protocol and characterization data for *L*^1^ are given in the Supporting Information. The synthesis of *L*^2^ followed our reported method.^34^ Unless otherwise stated, reagents and solvents were purchased commercially and used as supplied.

*Caution*! Although we have experienced no problems in using the perchlorate salts in this study, metal–organic perchlorates are potentially explosive and should be handled with care in small quantities.

### Synthesis of [Fe(*L*^1^)_2_][BF_4_]_2_ (**1[BF**_**4**_**]**_**2**_)

A mixture of *L*^1^ (50 mg, 0.16 mmol) and Fe[BF_4_]_2_·6H_2_O (27 mg, 0.080 mmol) in nitromethane (10 cm^3^) was stirred at room temperature until all the solid had dissolved. The orange solution was filtered, and the complex was precipitated by dropwise addition of diethyl ether (50 cm^3^). The orange powder was collected on a glass frit and washed with diethyl ether. Yield: 44 mg, 64%.

Solvate crystals of the complex were obtained by recrystallizing the crude powder from acetone, acetonitrile, or nitromethane by diethyl ether vapor diffusion. Monohydrate crystals of the complexes were produced similarly, from undried methanol solutions. The lattice solvent in the organic solvate crystals is replaced by atmospheric moisture upon exposure to air. Most microanalyses from samples of these materials were approximately consistent with a sesquihydrate formulation. Anal. Found: C, 37.6; H, 3.48; N, 15.5. Calcd for C_28_H_30_B_2_F_8_FeN_10_S_4_·1.5H_2_O: C, 37.7; H, 3.73; N, 15.7. ^1^H NMR (CD_3_NO_2_): δ 1.2 (12H, SCH{C*H*_3_}_2_), 3.1 (2H, SC*H*{CH_3_}_2_), 40.2 (4H, Py *H*^3/5^), 40.6 (4H, Pz *H*^5^), 59.3 (4H, Pz *H*^4^), 68.4 (4H, Pz *H*^3^).

A good microanalysis was obtained from one organic solvate formulation, produced by recrystallization from acetone/diethyl ether. Anal. Found: C, 40.8; H, 4.00; N, 14.9. Calcd for C_28_H_30_B_2_F_8_FeN_10_S_4_·C_3_H_6_O: C, 40.4; H, 3.93; N, 15.2.

### Synthesis of [Fe(*L*^1^)_2_][ClO_4_]_2_ (**1[ClO**_**4**_**]**_**2**_)

The same method as for **1[BF**_**4**_**]**_**2**_ was used, with Fe[ClO_4_]_2_·6H_2_O (29 mg, 0.080 mmol). The product was an orange powder. Yield: 51 mg, 72%.

Solvate crystals of **1[ClO**_**4**_**]**_**2**_ were produced as above and were similarly sensitive to solvent loss in air. Most samples of **1[ClO**_**4**_**]**_**2**_ also were analyzed to have a sesquihydrate formulation. Anal. Found: C, 36.7; H, 3.40; N, 15.4. Calcd for C_28_H_30_B_2_F_8_FeN_10_S_4_·1.5H_2_O: C, 36.7; H, 3.63; N, 15.3.

A good microanalysis was obtained from a solvent-free sample, produced by annealing a mixture of acetone solvate crystals at 370 K. Anal. Found: C, 37.6; H, 3.47; N, 15.4. Calcd for C_28_H_30_Cl_2_FeN_10_O_8_S_4_: C, 37.8; H, 3.40; N, 15.7.

### Synthesis of [Fe(*L*^2^)_2_][BF_4_]_2_ (**2[BF**_**4**_**]**_**2**_)

The same method as for **1[BF**_**4**_**]**_**2**_, with *L*^2^ (47 mg, 0.16 mmol). The product was an orange powder, which formed brown single crystals on recrystallization from MeCN or MeNO_2_ solution with a diethyl ether vapor. The crystals decomposed to a solvent-free powder on drying *in vacuo*. Yield: 57 mg, 88%. Anal. Found: C, 35.3; H, 2.80; N, 17.3. Calcd for C_24_H_22_B_2_F_8_FeN_10_S_4_: C, 35.7; H, 2.74; N, 17.3. ^1^H NMR (CD_3_NO_2_): δ 2.5 (6H, SC*H*_3_), 39.6 (4H, Py *H*^3/5^), 40.2 (4H, Pz *H*^5^), 58.6 (4H, Pz *H*^4^), 68.5 (4H, Pz *H*^3^).

### Single-Crystal X-ray Structure Analyses

Crystals of *L*^1^ were obtained upon slow evaporation of an NMR sample of that compound in CDCl_3_. Crystals of **1[BF**_**4**_**]**_**2**_·solv, **1[ClO**_**4**_**]**_**2**_·solv, and **2[BF**_**4**_**]**_**2**_·solv material were prepared as described above. The **1X**_**2**_·sf (X^–^ = BF_4_^–^, ClO_4_^–^) crystals were obtained by annealing crystals of **1X**_**2**_·Me_2_CO on the diffractometer at 370 K for 30 min. Where relevant, the same crystal was used for data collections at multiple temperatures.

All diffraction data were collected with an Agilent Supernova dual-source diffractometer using monochromated Cu Kα radiation (λ = 1.54184 Å). Experimental details of each structure determination and full details of all the crystallographic refinements, are given in Table S1 in the Supporting Information. The structures were solved by direct methods (SHELXS) and developed by full least-squares refinement on *F*^2^ (SHELXL-2018).^42^ Crystallographic figures were prepared using X-SEED,^43^ and structural parameters tabulated in the Supporting Information were calculated with Olex 2.^44^ Hirshfeld surface calculations were performed with CrystalExplorer.^45^

### Other Measurements

Elemental analyses were performed by the microanalytical services at the University of Leeds School of Chemistry or the London Metropolitan University School of Human Sciences. Electrospray mass spectra were recorded on a Bruker MicroTOF-q instrument from CHCl_3_ solution. Diamagnetic NMR spectra employed a Bruker AV3HD spectrometer operating at 400.1 (^1^H) or 100.6 MHz (^13^C), while paramagnetic ^1^H NMR spectra were obtained with a Bruker AV3 spectrometer operating at 300.1 MHz. X-ray powder diffraction data were measured at 298 K with a Bruker D2 Phaser diffractometer, using Cu Kα radiation (λ = 1.5419 Å). Some powder diffraction samples were coated in Nujol to protect against solvent loss during measurement; details are given in the Supporting Information. Thermogravimetric analyses were obtained with a TA Instruments TGA Q50 analyzer heating at a rate of 10 K min^–1^ under a stream of nitrogen gas.

Magnetic susceptibility measurements were performed using a Quantum Design MPMS-3 VSM magnetometer, in an applied field of 5000 G. Unless otherwise stated, samples were measured at a scan rate of 5 K min^–1^. Diamagnetic corrections for the samples were estimated from Pascal’s constants;^46^ a diamagnetic correction for the sample holder was also applied to the data. Samples were protected against solvent loss by saturating the tightly sealed MPMS-3 powder capsules with diethyl ether vapor, although the acetone solvates often desolvated rapidly *in situ* despite that precaution (Figure S25).

Susceptibility measurements in solution were obtained by the Evans method using a Bruker AV-NEO spectrometer operating at 500.2 MHz.^47^ A diamagnetic correction for the sample^46^ and a correction for the variation of the density of the CD_3_CN solvent with temperature^48^ were applied to these data.

## Results and Discussion

The reaction of bpp^SH 49^ with 2-iodopropane or iodomethane in refluxing acetonitrile, in the presence of potassium carbonate, affords a mixture including bpp^SR^′^^ (R′ = Me, *i*Pr), bpp^SSR^′^^ (i.e. *L*^1^ or *L*^2^; Chart 1) and bis(2,6-bis(pyrazol-1-yl)pyrid-4-yl) disulfide. These were separated by a sequence of precipitation and chromatography steps, from which *L*^1^ and *L*^2^ can be isolated in 20–30% yield.^39^ We obtained a significant quantity of *L*^1^ during our studies of the [Fe(bpp^S*i*Pr^)_2_]X_2_·solv system,^19,36,37^ allowing us to investigate its iron chemistry in detail. Since *L*^2^ was only available in smaller amounts, however, fewer experiments were undertaken with that ligand.^34^

The complex salts **1[BF**_**4**_**]**_**2**_, **1[ClO**_**4**_**]**_**2**_, and **2[BF**_**4**_**]**_**2**_ were obtained by treatment of Fe[BF_4_]_2_·6H_2_O or Fe[ClO_4_]_2_·6H_2_O with 2 equiv of the appropriate ligand in nitromethane. Addition of excess diethyl ether afforded the complexes as orange powders, which were recrystallized from different organic solvents by diethyl ether vapor diffusion. Dried polycrystalline **1[BF**_**4**_**]**_**2**_ and **1[ClO**_**4**_**]**_**2**_ readily absorb atmospheric moisture and consistently analyzed as having the formulations **1[BF**_**4**_**]**_**2**_·1.5H_2_O and **1[ClO**_**4**_**]**_**2**_·1.5H_2_O. Dried samples of **2[BF**_**4**_**]**_**2**_ were solvent-free, as determined by elemental analysis.

Recrystallization of **1[BF**_**4**_**]**_**2**_ and **1[ClO**_**4**_**]**_**2**_ from acetone/diethyl ether yielded mixtures of crystal phases, which could be distinguished by their color and morphology. These included yellow needles of the composition **1X**_**2**_·Me_2_CO (X^–^ = BF_4_^–^, ClO_4_^–^; monoclinic, space group *P*2_1_/*c*, *Z* = 4), whose metric parameters show that they are high-spin at 250 K but low spin at 143 K (X^–^ = BF_4_^–^) or 100 K (X^–^ = ClO_4_^–^). Variable-temperature unit cell data confirm that both crystals undergo abrupt spin transitions near 150 K, with a 10 K thermal hysteresis being measured for the perchlorate salt (Table 1 and Figures S7–S10).^50^

**Table 1:** Summary of the Solvate Crystal Phases Obtained in This Work and Their Spin-State Properties

phase	spin-state properties, *T*_1/2_ (K)
**1[BF**_**4**_**]**_**2**_·Me_2_CO	*T*_1/2_↓ = 175 ± 5, *T*_1/2_↑ = 175 ± 5^a^^,^^b^
**1[ClO]**_**2**_·Me_2_CO	*T*_1/2_↓ = 155 ± 5, *T*_1/2_↑ = 165 ± 5^a^
	*T*_1/2_↓ = 151, *T*_1/2_↑ = 173^c^
	
**1[BF**_**4**_**]**_**2**_·0.75Me_2_CO	1:1 low-spin:high-spin at 250 K^a^
**1[BF**_**4**_**]**_**2**_·0.5Me_2_CO·0.5H_2_O	low-spin at *T* ≤ 250 K^a^
**1[ClO**_**4**_**]**_**2**_·*m*Me_2_CO·0.5H_2_O	gradual SCO; *T*_1/2_ = 325 ± 2^c^
	
**1[BF**_**4**_**]**_**2**_·MeNO_2_	gradual SCO; *T*_1/2_ = 270^c^
**1[ClO**_**4**_**]**_**2**_·*n*MeNO_2_	gradual SCO; *T*_1/2_ = 264^c^
	
**1[BF**_**4**_**]**_**2**_·MeCN	gradual SCO; *T*_1/2_ = 316^c^
**1[ClO**_**4**_**]**_**2**_·MeCN	gradual SCO; *T*_1/2_ = 299^c^^,^^d^
	
**1[BF**_**4**_**]**_**2**_·H_2_O	gradual SCO; *T*_1/2_ = 342^c^
**1[ClO**_**4**_**]**_**2**_·H_2_O	gradual SCO; *T*_1/2_ = 321^c^
	
**1[BF**_**4**_**]**_**2**_·sf	*T*_1/2_↓ = 127.5 ± 2.5, *T*_1/2_↑ = 165 ± 5^a^
	*T*_1/2_↓ = 135, *T*_1/2_↑ = 159^c^^,^^e^
	
**1[ClO**_**4**_**]**_**2**_·sf	*T*_1/2_↓ = 165 ± 5, *T*_1/2_↑ = 185 ± 5^a^
	*T*_1/2_↓ = 174, *T*_1/2_↑ = 184^c^

a From crystallographic data.

b A magnetic measurement of this transition from a phase-pure sample was not achieved. See also ref (50).

c From magnetic susceptibility data.

d These magnetic data are inconsistent with the crystal structure of this compound. See the text for more details.

e SCO is incomplete in the magnetic measurements, because a fraction of the sample is kinetically trapped in its high-spin state below the transition temperature.

The solvate **1[BF**_**4**_**]**_**2**_·Me_2_CO cocrystallized with two brown pseudopolymorphs with needle and prismatic morphologies, with the respective formulas **1[BF**_**4**_**]**_**2**_·0.75Me_2_CO (triclinic, *P*1̅, *Z* = 4) and **1[BF**_**4**_**]**_**2**_·0.5Me_2_CO·0.5H_2_O (monoclinic, *P*2_1_/*c*, *Z* = 8). Both of these solvates contain two unique complex molecules per asymmetric unit. Molecule A of **1[BF**_**4**_**]**_**2**_·0.75Me_2_CO is low-spin, while molecule B is high-spin at 250 K. However, at 120 K molecule B exhibits whole-ligand disorder, implying a ca. 3:1 high- to low-spin population, which indicates the onset of SCO at that temperature. In contrast, both cation environments in **1[BF**_**4**_**]**_**2**_·0.5Me_2_CO·0.5H_2_O are low-spin at both 120 and 250 K.

One brown single-crystalline contaminant was noted in samples of **1[ClO**_**4**_**]**_**2**_·Me_2_CO, namely **1[ClO**_**4**_**]**_**2**_·*m*Me_2_CO·0.5H_2_O (*m* ≈ 0.34; monoclinic, *P*2_1_/*c*, *Z* = 8). This is not isomorphous with **1[BF**_**4**_**]**_**2**_·0.5Me_2_CO·0.5H_2_O but, like that compound, **1[ClO**_**4**_**]**_**2**_·*m*Me_2_CO·0.5H_2_O is fully low-spin at 120 K (an attempted measurement at higher temperature led to crystal decomposition). A residual low-temperature paramagnetism in fresh samples of “**1[ClO**_**4**_**]**_**2**_·*x*Me_2_CO” (see below) implies that a third phase may also be present in those samples, but it was not isolated as a pure (poly)crystalline material.

The acetone solvate crystals of **1[BF**_**4**_**]**_**2**_ and **1[ClO**_**4**_**]**_**2**_ were manually separated for characterization by X-ray powder diffraction (Figures S20 and S21). Those samples were each phase-pure, implying that there were no other uncharacterized materials in the mixtures. However, useful powder patterns were only obtained when the samples were coated with Nujol, to protect them against solvent loss. This sensitivity also made it hard to obtain consistent TGA or magnetic measurements from the individual acetone solvate phases. After several attempts, consistent magnetic data were obtained from pure samples of **1[ClO**_**4**_**]**_**2**_·Me_2_CO and **1[ClO**_**4**_**]**_**2**_·*m*Me_2_CO·0.5H_2_O. However, the BF_4_^–^ solvates could only be magnetically characterized as a mixture of the **1[BF**_**4**_**]**_**2**_·Me_2_CO, **1[BF**_**4**_**]**_**2**_·0.75Me_2_CO, and **1[BF**_**4**_**]**_**2**_·0.5Me_2_CO·0.5H_2_O phases, which is labeled “**1[BF**_**4**_**]**_**2**_·*x*Me_2_CO” in the following discussion.

**Figure 1:**
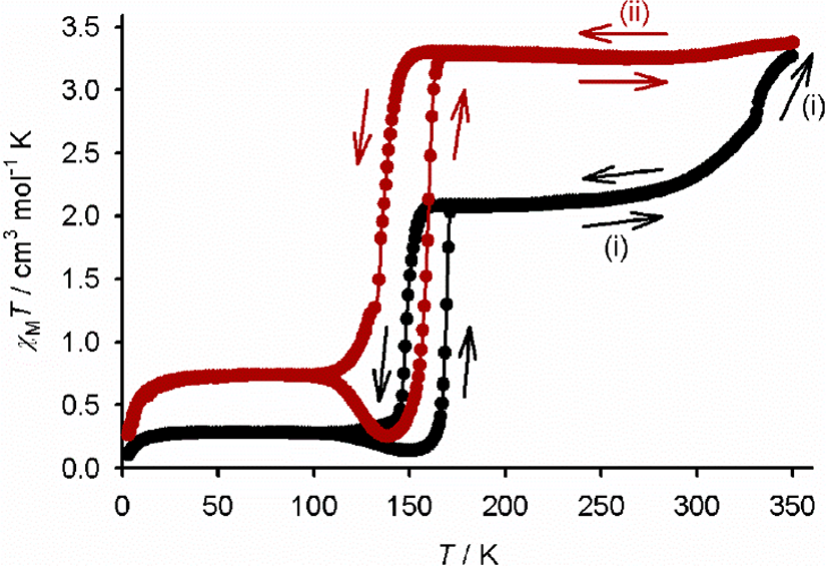
Magnetic susceptibility measurement for a mixed-phase sample of “**1[BF**_**4**_**]**_**2**_·*x*Me_2_CO”, showing its *in situ* conversion to **1[BF**_**4**_**]**_**2**_·sf: (i) first cycle, 300 → 3 → 350 K (black); (ii) second cycle, 350 → 3 → 300 K (red). Data points are connected by spline curves for clarity. Scan rate: 5 K min^–1^.

Mixed “**1[BF**_**4**_**]**_**2**_·*x*Me_2_CO” samples show χ_M_*T* = 2.0 ± 0.2 cm^3^ mol^–1^ K at 300 K, indicating a mixed high-spin → low-spin population at room temperature. This stays roughly constant on cooling until 150 K, where an abrupt decrease in χ_M_*T* is observed, corresponding to an abrupt high-spin → low-spin transition (Figure 1). A constant residual high-spin fraction with χ_M_*T* = 0.5 ± 0.2 cm^3^ mol^–1^ K remains on further cooling. The reverse low-spin → high spin-transition occurs at *T*_1/2_ = 168 ± 1 K on rewarming. This is always preceded by a small, gradual decrease in χ_M_*T* between 100 and 150 K, which is characteristic for the thermal trapping of some SCO-active material in its high-spin form at such low temperatures.^51−55^ This has also been seen in salts of other [Fe(bpp^R^)_2_]^2+^ derivatives showing cooperative SCO at temperatures approaching 100 K.^37,56−58^

While the temperature of the partial abrupt spin transition in Figure 1 is consistent with single crystals of **1[BF**_**4**_**]**_**2**_·Me_2_CO (Figures S7 and S8), the hysteresis loop is wider than expected from the unit cell data (Table 1).^50^ This might be explained by the faster temperature ramp in the magnetic measurement, which can widen kinetic hysteresis in an SCO material.^59^ Alternatively, it might reflect the onset of solvent loss from the sample in the high-vacuum magnetometer cavity. In any case, the magnitude of the abrupt spin transition implies samples of “**1[BF**_**4**_**]**_**2**_·*x*Me_2_CO” contain between 35 and 55% of the cooperative SCO phase **1[BF**_**4**_**]**_**2**_·Me_2_CO.

**Figure 2:**
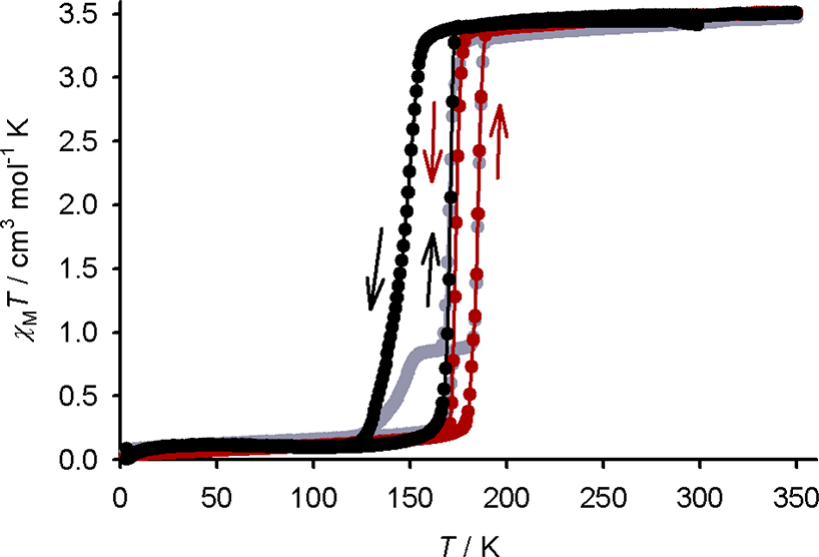
Magnetic susceptibility measurement for phase-pure **1[ClO**_**4**_**]**_**2**_·Me_2_CO, showing its *in situ* conversion to **1[ClO**_**4**_**]**_**2**_·sf. Three consecutive thermal scans are shown: (i) 300 → 3 → 350 K (black); (ii) 350 → 3 → 350 K (gray); (iii) 350 → 3 → 300 K (red). Other details are as for Figure 1.

Yellow **1[ClO**_**4**_**]**_**2**_·Me_2_CO is high-spin at room temperature and exhibits a complete, hysteretic spin transition centered at 162 K (Figure 2). As for the BF_4_^–^ salt, the 22 K hysteresis width in the magnetic measurement is larger than that in the single crystal. The discrepancy for this compound is only just outside the error of the crystallographic measurement, however (Table 1). In contrast, the brown material **1[ClO**_**4**_**]**_**2**_·*m*Me_2_CO·0.5H_2_O exhibits gradual SCO with *T*_1/2_ ≈ 325 K, which is ca. 80% complete at 350 K (Figure S24).

Heating “**1[BF**_**4**_**]**_**2**_·*x*Me_2_CO” and **1[ClO**_**4**_**]**_**2**_·Me_2_CO to 350 K converts them to a new single-phase material, which was assigned as solvent-free **1X**_**2**_·sf from the single-crystal experiments described below (Figures 1 and 2). The desolvation of “**1[BF**_**4**_**]**_**2**_·*x*Me_2_CO” occurs rapidly in the magnetometer, within one thermal scan, but three or four scans were required for full conversion of **1[ClO**_**4**_**]**_**2**_·Me_2_CO to **1[ClO**_**4**_**]**_**2**_·sf. Interestingly, all components of the “**1[BF**_**4**_**]**_**2**_·*x*Me_2_CO” mixture transform to the same **1[BF**_**4**_**]**_**2**_·sf material under these conditions. This was also observed for “**1[ClO**_**4**_**]**_**2**_·*x*Me_2_CO” mixed phase samples (Figure S25).

The annealed **1X**_**2**_·sf samples are fully high spin at room temperature and also exhibit abrupt spin transitions below 200 K. The spin transitions in both annealed materials also exhibit thermal hysteresis. Interestingly, SCO in **1[BF**_**4**_**]**_**2**_·sf occurs at ca. 15 K lower temperature than for **1[BF**_**4**_**]**_**2**_·Me_2_CO, with a wider thermal hysteresis (Figure 1). However, the opposite is observed for the perchlorate salt; *T*_1/2_ for **1[ClO**_**4**_**]**_**2**_·sf shifts to ca. 10 K higher temperature, with a narrower hysteresis, after the desolvation process (Figure 2). A possible explanation for these differences is discussed below. Thermal trapping of a residual high-spin fraction of the sample was also observed during SCO in **1[BF**_**4**_**]**_**2**_·sf, but not for **1[ClO**_**4**_**]**_**2**_·sf. Thermal trapping in **1[BF**_**4**_**]**_**2**_·sf occurs more efficiently on measurement at a faster scan rate, confirming its kinetic origin (Figure S23).^37,51−58^

**Figure 3:**
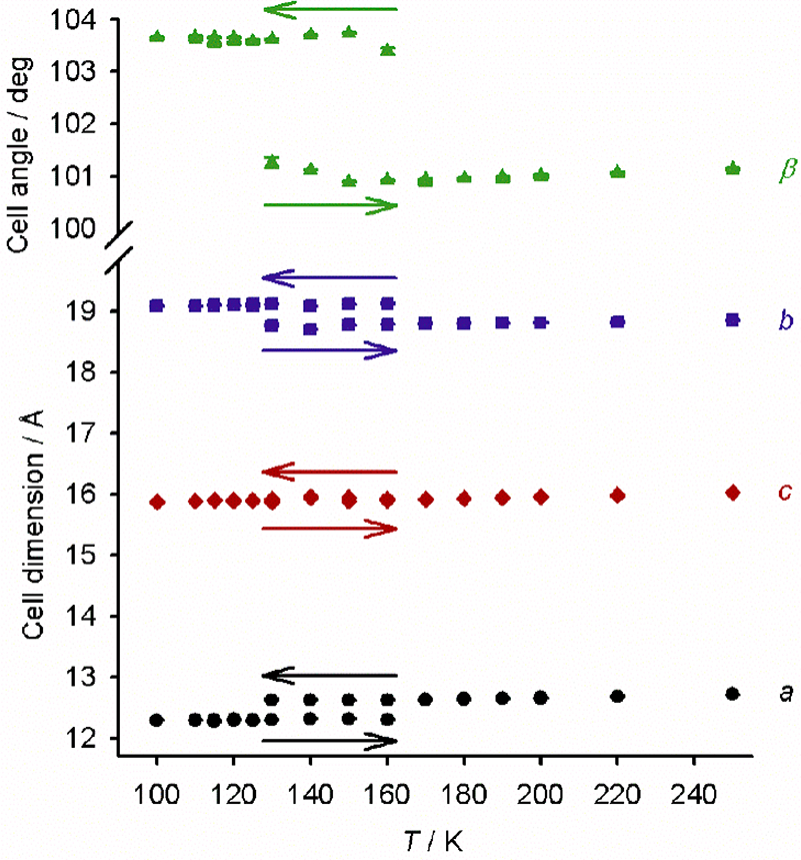
Variable-temperature unit cell parameters for **1[BF**_**4**_**]**_**2**_·sf, measured in cooling and warming modes and showing thermal hysteresis in the spin transition (Table S9).

Heating crystals of **1X**_**2**_·Me_2_CO (X^–^ = BF_4_^–^, ClO_4_^–^) at 370 K on the diffractometer caused a rapid transformation to **1X**_**2**_·sf (monoclinic, space group *P*2_1_/*n*, *Z* = 4), without degradation of crystal quality. Unit cell determinations from **1X**·sf confirmed that their spin-transition temperatures match the magnetic data from the annealed “**1X**_**2**_·*x*Me_2_CO” samples (Figure 3 and Figures S17–S19). However, the crystallographic SCO hysteresis loops for both **1X**_**2**_·sf crystals are a few degrees *wider* than in the magnetic data, which is opposite to the trend expected if the hysteresis were controlled by the thermal scan rate (Table 1).^59^ Rather, it might reflect the improved crystallinity and larger particle size of a single crystal of **1X**_**2**_·sf, in comparison to a polycrystalline sample from annealing a mixture of precursor phases.^60−63^

### Structural Comparison of **1X_2_**·Me_2_CO and **1X_2_**·sf

**Figure 4:**
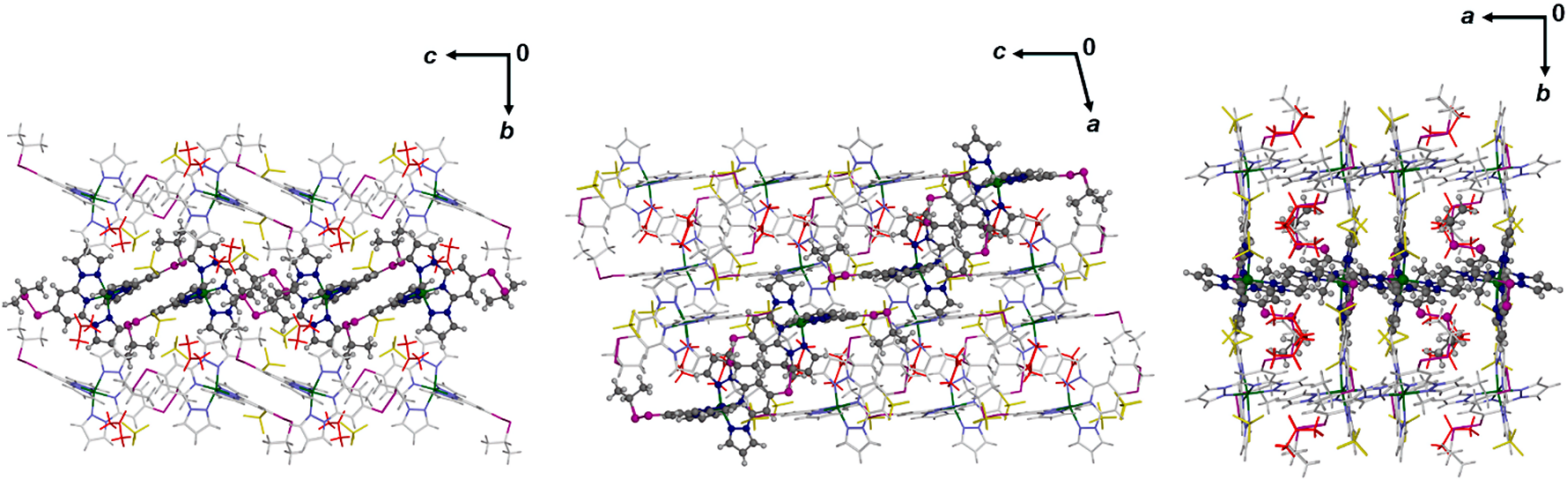
Packing diagrams of low-spin **1[BF**_**4**_**]**_**2**_·Me_2_CO at 143 K, viewed along the [100] (left), [010] (center), and [001] (right) crystal vectors. One chain of cations linked by n···π interactions is highlighted in each diagram, and the directions of the unit cell axes are shown for each view. Color code: C{complex}, white or dark gray; H{complex}, pale gray; N, pale or dark blue; S, purple; BF_4_^–^, yellow; solvent, red.

The unit cells of **1X**_**2**_·sf (in the space group setting *P*2_1_/*n*) resemble those of the precursor **1X**_**2**_·Me_2_CO crystals (in the setting *P*2_1_/*c*), but with the *b* and *c* axes exchanged; that is, *a* ≈ *a*′, *b* ≈ *c*′, *c* ≈ *b*′ and β ≈ β′. The cations in **1X**_**2**_·Me_2_CO are roughly coaligned, but with alternate canting of their molecular *z* axes about the crystallographic *c* direction (Figure 4). Cations related by a crystallographic inversion center exchange intermolecular n···π contacts through the β-S atom of each SS*i*Pr group. One of these n···π contacts is formed to a pyridyl ring from the neighboring molecule, while the other involves a pyrazolyl group. These pairwise n···π interactions propagate into chains parallel to the [101] crystal vector.

**Figure 5:**
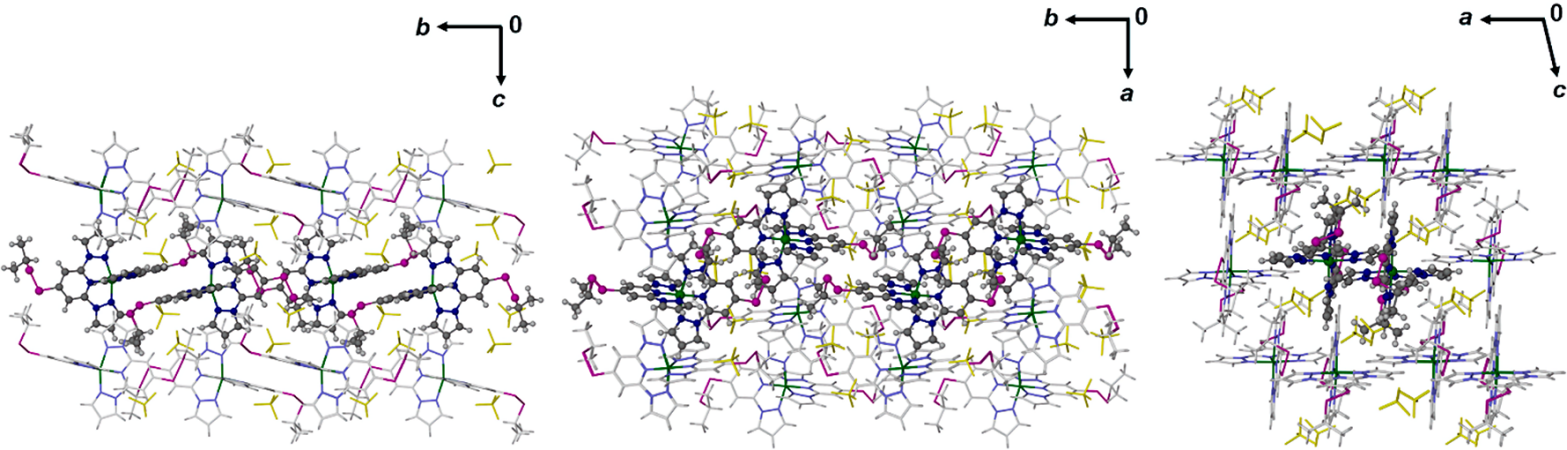
Packing diagrams of **1[BF**_**4**_**]**_**2**_·sf at 100 K, viewed along the [100] (left), [001] (center), and [010] (right) crystal vectors. The views are arranged to facilitate comparison with Figure 4. Details are as for Figure 4.

The chain of n···π dimers motif is retained in **1X**_**2**_·sf. One dimerization interaction is geometrically similar in both lattice types. However, each pair of cations in **1X**_**2**_·sf is translated by 1 + *x*, *y*, *z* in comparison to their equivalent positions in **1X**_**2**_·Me_2_CO, so that those S atoms interact with opposite faces of the heterocyclic ligand in the two lattice types. That gives the chains in **1X**_**2**_·sf a zigzag geometry, aligned along the [010] vector (Figure 5).

The closest S···π distances for each interaction in the low-spin structures range from 3.26 to 3.44 Å for **1X**_**2**_·Me_2_CO and 3.31–3.50 Å for **1X**_**2**_·sf; these values are generally longer in the high-spin forms of the crystals (Tables S3 and S8). For comparison, the sum of the Pauling van der Waals radii of an S atom and an aromatic ring is 3.55 Å.^64^ Hirshfeld surface analyses of these structures also highlight weak C–H···Y (Y = F, O) and/or anion···π contacts between the cations and anions in some of the structures (Figures S35–S37).^65^ These secondary interactions are less likely to contribute to SCO cooperativity, however, since they do not directly link the cation switching centers in the materials.

Structures of both **1X**_**2**_·sf crystals were determined at 250 K, when they were high-spin, and at 100 or 110 K. Both the low-spin and high-spin states of **1[BF**_**4**_**]**_**2**_·sf were achieved at 100 K, using the same crystal. This reflects the slow kinetics for the high-spin → low-spin transition observed in the magnetic data (Figure 1). Thus, the crystal was thermally trapped in its high-spin form when it was first cooled from 250 to 100 K on the diffractometer,^57,66−70^ but a subsequent, duplicate experiment yielded the low-spin state at 100 K. The different outcomes might be caused by small differences in the temperature ramp in the two experiments. Alternatively, they could reflect the introduction of additional defects or a reduction in domain size in the crystal following the first thermal cycle.^60−63^ While thermal trapping of **1[ClO**_**4**_**]**_**2**_·sf was not observed, isothermal high- and low-spin structures of that compound were achieved at 170 K, a temperature inside its SCO hysteresis loop.

Although the orientations of their *i*Pr substituents are different, in other respects the molecular structures of **1X**_**2**_·Me_2_CO and **1X**_**2**_·sf are very similar. Each shows a comparable displacement of one *L*^1^ ligand relative to the other in the complex during SCO, as quantified by the *trans*-N{pyridyl}–Fe–N{pyridyl} bond angle (ϕ; Table 2).^74^ The four crystals show 163.01(13) ≤ ϕ ≤ 166.37(12)° when they are high-spin, which is a significant deviation from the ideal value of 180°. High-spin [Fe(bpp^R^)_2_]^2+^ derivatives can show large distortions from idealized *D*_2*d*_ symmetry through reduced values of ϕ and of the dihedral angles between the least-squares planes of the two ligands (θ; Table 2).^28^ SCO in the solid state becomes more difficult as ϕ and θ deviate more strongly from the more regular geometries preferred by the low-spin complexes.^27^ The values of ϕ in **1X**_**2**_·Me_2_CO and for **1X**_**2**_·sf lie in a range where SCO is possible but is rarely observed in practice.^57^ The high-spin molecular geometries of **1X**_**2**_·sf appear to show a small temperature dependence, as we have observed before in some related compounds.^19,75^ More detailed investigations would be required to quantify that, however.

**Table 2:** Crystallographic Spin-Transition Temperatures for the **1X**_**2**_·Me_2_CO and **1X**_**2**_·sf Phases and Structural Changes during Their Thermal SCO^a^^,^^b^

	**1[BF**_**4**_**]**_**2**_·Me_2_CO	**1[ClO]**_**2**_·Me_2_CO	**1[BF**_**4**_**]**_**2**_·sf	**1[ClO]**_**2**_·sf
*T*_1/2_↓ (K)	175 ± 5^c^	155 ± 5	127.5 ± 2.5	165 ± 5
*T*_1/2_↑ (K)	175 ± 5^c^	165 ± 5	165 ± 5	185 ± 5
Δ*T*_1/2_ (K)		10 ± 7	38 ± 6	20 ± 7
Δ*V*_*O_h_*_ (Å^3^)	2.419(15)	2.439(14)	2.555(17) [2.476(13)]	2.548(15) [2.458(17)]
ΔΣ (deg)	64.8(6)	64.8(5)	70.0(7) [69.2(6)]	67.3(6) [63.1(6)]
ΔΘ (deg)	230	231	225 [216]	218 [202]
Δϕ (deg)	–11.32(16)	–11.47(15)	–11.99(19) [−13.03(16)]	–11.39(17) [−11.24(19)]
Δθ (deg)	–1.29(6)	–1.30(4)	–1.88(6) [−2.34(4)]	–1.06(5) [−1.15(5)]

a Δ*V*_*O_h_*_ = *V*_*O_h_*_(high-spin) – *V*_*O_h_*_(low-spin). The other parameters in the table are calculated similarly. The parameters are computed from high- and low-temperature crystal structures, with the values in brackets for **1X**_**2**_·sf being calculated from their isothermal high-spin and low-spin structure refinements. More detailed metric parameters are given in Tables S2 and S7

b *V*_*O_h_*_ is the volume of the octahedron defined by the FeN_6_ coordination sphere.^71^ Σ is a general measure of the deviation of a metal ion from an ideal octahedral geometry, while Θ more specifically indicates its distortion toward a trigonal-prismatic structure.^71−73^ ϕ is the *trans*-N{pyridyl}–Fe–N{pyridyl} bond angle, while θ is the dihedral angle between the least-squares planes of the two tridentate ligands.^74^ More detailed definitions and discussions of these parameters are in the cited references, and in the Supporting Information to this article.

c See ref (50).

**Figure 6:**
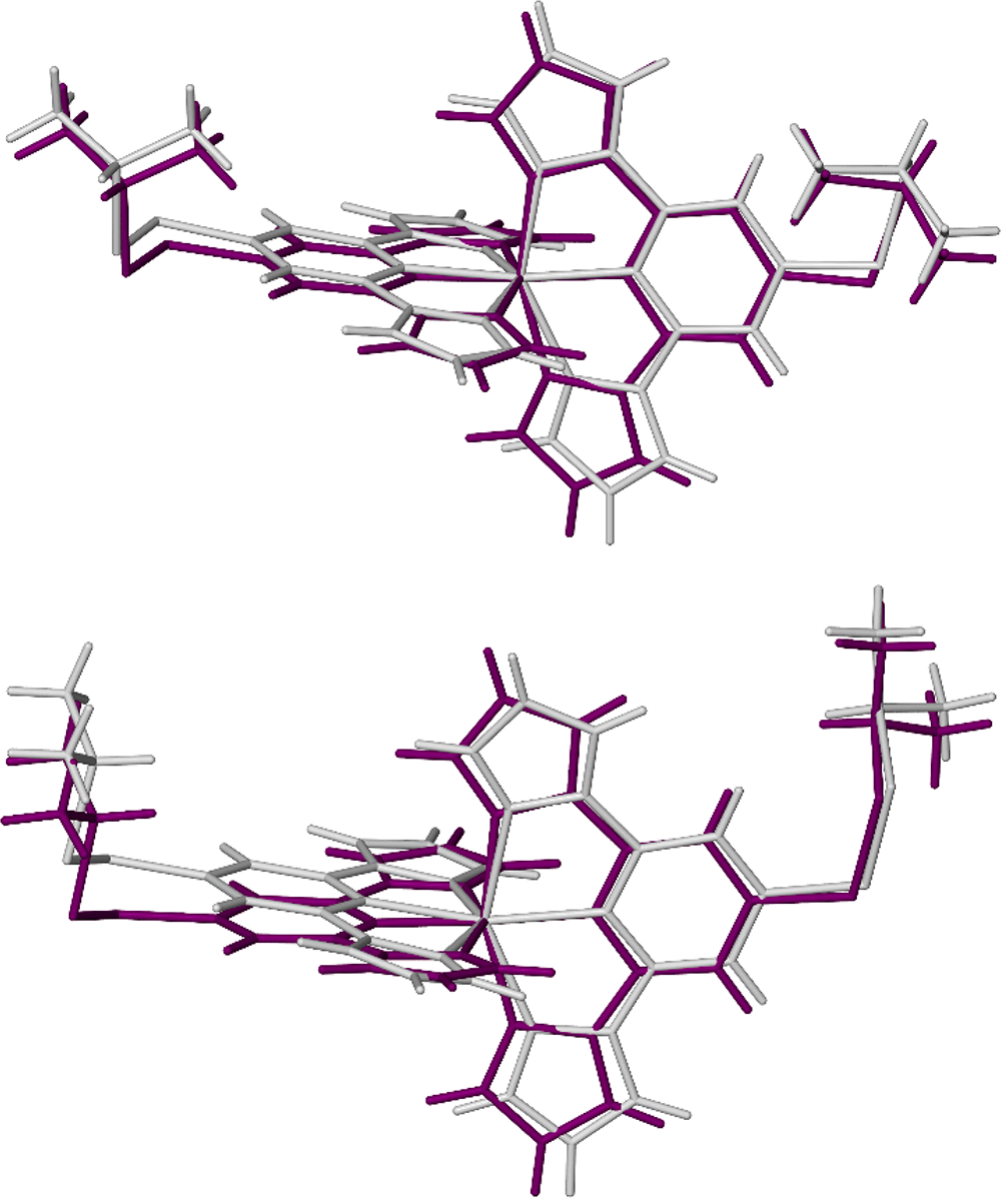
Overlaid high-spin (white) and low-spin (purple) structures of **1[BF**_**4**_**]**_**2**_·Me_2_CO (top) and **1[BF**_**4**_**]**_**2**_·sf (bottom), showing the angular displacement of the *L*^1^ ligands during SCO. Only the major orientation of the disordered isopropyl residue in high-spin **1[BF**_**4**_**]**_**2**_·Me_2_CO is shown. The **1[BF**_**4**_**]**_**2**_·sf view was generated from the isothermal high- and low-spin structures of that compound at 100 K.

The low-spin forms of the compounds have more regular geometries with 174.33(10) ≤ ϕ ≤ 177.61(13)°. The change in ϕ between the spin states, Δϕ, is 11–13° (Table 2), which leads to a large, anisotropic geometric rearrangement of the molecules in the lattice during SCO (Figure 6). Such Δϕ values are unusually large for an SCO-active [Fe(bpp^R^)_2_]^2+^ derivative and are associated with cooperative hysteretic spin transitions when they have been observed before.^20,57,76,77^ Notably **1[BF**_**4**_**]**_**2**_·sf, which shows a wider hysteresis loop in comparison to the other compounds in Table 2, has both a larger Δϕ value and slightly higher Δθ value, which supports this structure–function relationship. These changes lead to lateral displacements of the peripheral atoms in the molecules, of up to 1.0 Å, which will be transmitted efficiently through the lattice by the intermolecular n···π interactions described above. This is the likely origin of the cooperative, hysteretic spin transitions in **1X**_**2**_·Me_2_CO and **1X**_**2**_·sf.

### Other **1X**_**2**_·solv Materials

Recrystallization of the **1X**_**2**_ salts from undried nitromethane, acetonitrile, or methanol yielded visually homogeneous samples of **1X**_**2**_·MeNO_2_, **1X**_**2**_·MeCN, and **1X**_**2**_·H_2_O, respectively. Crystals of **1[BF**_**4**_**]**_**2**_·MeNO_2_ and **1[ClO**_**4**_**]**_**2**_·*n*MeNO_2_ (*n* ≈ 0.9; both monoclinic, *P*2_1_/*n*, *Z* = 4) are isomorphous. The perchlorate crystal was slightly substoichiometric in nitromethane, which might reflect a steric clash between the solvent molecule and a neighboring, disordered ClO_4_^–^ anion. Crystals of **1[BF**_**4**_**]**_**2**_·H_2_O (monoclinic, *P*2_1_/*n*, *Z* = 4) are isomorphous with the nitromethane solvates and, although they were not crystallographically characterized, the X-ray powder patterns from **1[BF**_**4**_**]**_**2**_·MeCN and **1[ClO**_**4**_**]**_**2**_·H_2_O imply that they are also isomorphous with these materials (Figures S32 and S33). However, **1[ClO**_**4**_**]**_**2**_·MeCN (triclinic, *P*1̅, *Z* = 4) adopts a different symmetry, with two unique cations in its asymmetric unit. All of these materials are phase-pure by powder diffraction except for **1[ClO**_**4**_**]**_**2**_·MeCN, whose powder pattern is different from the others and does not agree well with the crystallographic simulation. Although no other single-crystal morphologies were apparent for that compound, bulk samples of **1[ClO**_**4**_**]**_**2**_·MeCN appear to contain a mixture of phases.

**Figure 7:**
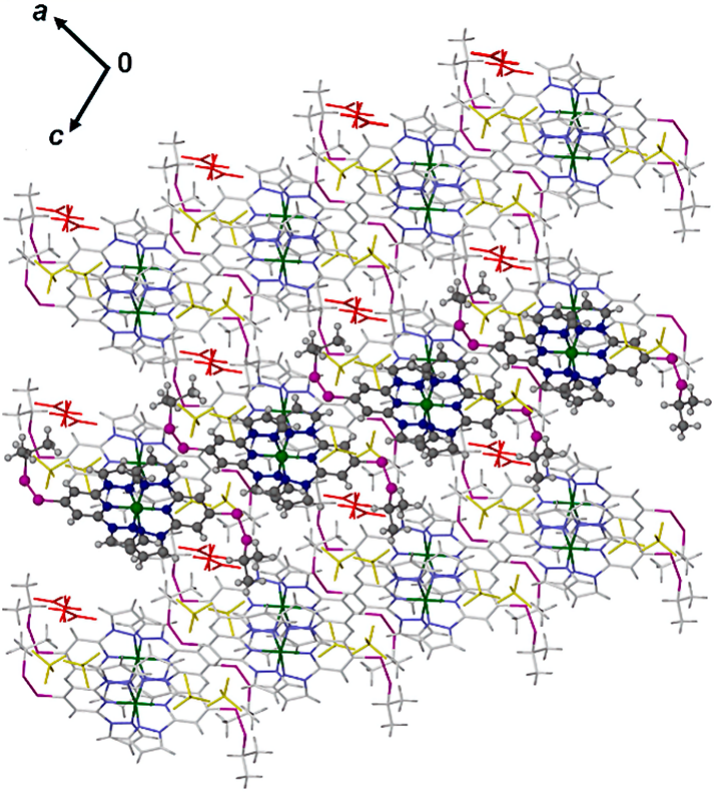
Packing diagram of **1[BF**_**4**_**]**_**2**_·MeNO_2_ at 120 K, viewed along the [010] vector. Only one orientation of the disordered residues in the structure is shown. One chain of cations linked by pairwise n···π interactions is highlighted, and the directions of the unit cell axes are shown. Color code: C{complex}, white or dark gray; H{complex}, pale gray; N, pale or dark blue; S, purple; BF_4_^–^, yellow; solvent, red.

The unit cell parameters of **1[BF**_**4**_**]**_**2**_·MeNO_2_, **1[BF**_**4**_**]**_**2**_·H_2_O, and **1[ClO**_**4**_**]**_**2**_·*n*MeNO_2_ (in the space group setting *P*2_1_/*n*) are also essentially identical with those of **1X**_**2**_·Me_2_CO (X^–^ = BF_4_^–^, ClO_4_^–^; in the setting *P*2_1_/*c*), with *a* ≈ *a*′′, *b* ≈ *b*′′, *c* ≈ *c*′′, and β ≈ β′. However, despite that coincidental similarity, the crystal packing in the two solvate lattices is quite different. The cations in **1[BF**_**4**_**]**_**2**_·MeNO_2_, **1[BF**_**4**_**]**_**2**_·H_2_O, and **1[ClO**_**4**_**]**_**2**_·*n*MeNO_2_ also associate into chains through intermolecular *n*···π interactions, involving sulfur atom lone pairs. However, pairs of interacting molecules in this lattice are related by a crystallographic *C*_2_ axis, which associates them loosely into chains parallel to the [101] vector (Figure 7).

The intermolecular S···π distances in this lattice type range from 3.36 to 3.60 Å and are slightly longer than in the more cooperative **1X**_**2**_·Me_2_CO and **1X**_**2**_·sf low-spin crystals (Table S13). While they are complicated by disorder, Hirshfeld surface analyses confirm that there are no short, directional intermolecular interactions in these lattices (Figure S38).^65^ Despite that, however, the overall packing density in this lattice type is greater than in the more cooperative materials, which is evidenced by the crystallographic density (*D*_c_) of the compounds. For example, **1[ClO**_**4**_**]**_**2**_·Me_2_CO (*M*_r_ = 947.69) has *D*_c_ = 1.593 g cm^–3^ at 100 K, while **1[ClO**_**4**_**]**_**2**_·*n*MeNO_2_ (*M*_r_ = 944.55) gives *D*_c_ = 1.614 g cm^–3^ at the slightly higher temperature of 120 K.

The MeNO_2_ and MeCN solvates are more stable to solvent loss in comparison to the acetone solvate crystals. These samples afforded TGA analyses consistent with their crystallographic formulations (Figure S31) and reproducible magnetic data. The hydrate crystals easily lose their lattice water on heating by TGA but also regain it quickly on re-exposure to air. However, those samples also gave reproducible magnetic data when they were protected against solvent loss.

**Figure 8:**
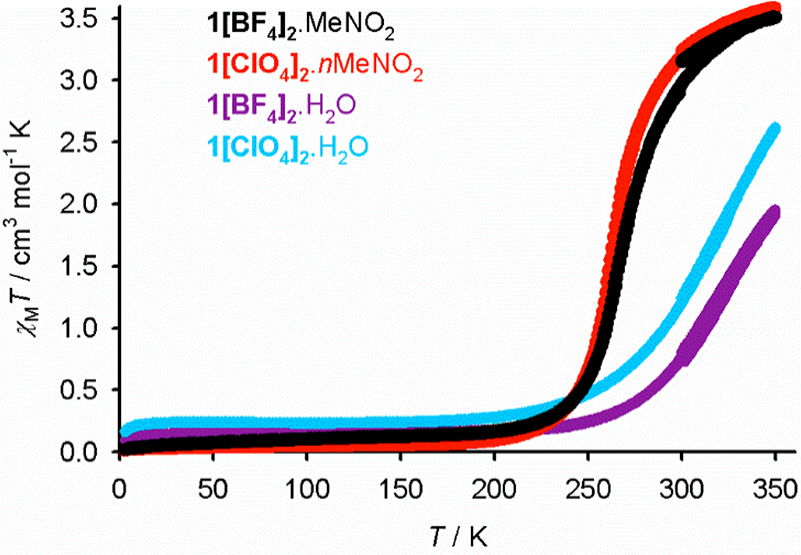
Variable-temperature magnetic susceptibility data for the isomorphous **1X**_**2**_·MeNO_2_ and **1X**_**2**_·H_2_O materials. Data were measured on a 300 → 350 → 3 → 300 K thermal cycle, at a scan rate of 5 K min^–1^.

The MeNO_2_, MeCN, and H_2_O solvates all exhibit gradual SCO equilibria as shown by magnetic susceptibility data, with 264 ≤ *T*_1/2_ ≤ 342 K (Figure 8 and Figure S34). Their high-temperature susceptibility behavior was reversible at temperatures up to 350 K, showing that these spin-state changes are not associated with *in situ* solvent loss. The spin states shown by the magnetic data at different temperatures agree well with the crystallographic predictions, except for **1[ClO**_**4**_**]**_**2**_·MeCN. The two unique cation environments in that crystal are both low-spin at 120 K and predominantly high-spin at 250 K, implying they undergo SCO between those temperatures. However, the bulk material undergoes gradual SCO at higher temperature and is only 20% high-spin at 250 K in the magnetic data. As was mentioned above, this sample apparently contained a mixture of phases by powder diffraction; thus, the single-crystal structures of **1[ClO**_**4**_**]**_**2**_·MeCN are not representative of that bulk sample.

The isomorphous **1X**_**2**_·MeNO_2_, **1X**_**2**_·MeCN, and **1X**_**2**_·H_2_O crystals could not be characterized in their high-spin form without crystal decomposition from solvent loss. Hence, it is unclear whether their SCO is associated with smaller structural changes between their spin states in comparison to those in the more cooperative **1X**_**2**_·Me_2_CO and **1X**_**2**_·sf series.^78^

Annealing crystals of **1[BF**_**4**_**]**_**2**_·MeNO_2_ and **1[BF**_**4**_**]**_**2**_·H_2_O at 370 K for 1 h on the diffractometer afforded the same **1[BF**_**4**_**]**_**2**_·sf phase described above. These annealed crystals were often twinned but retained their single crystallinity on some occasions. The transformation is not evident in the magnetic data from the same phases, however, implying that it requires conditions more forcing than those for the acetone solvates (Figure 8).

### Spin Crossover in **2[BF**_**4**_**]**_**2**_

Since *L*^2^ was available in small quantities, only one salt of its iron complex was investigated, **2[BF**_**4**_**]**_**2**_. Two isomorphous solvates of this material were structurally characterized, **2[BF**_**4**_**]**_**2**_·0.5MeNO_2_ and **2[BF**_**4**_**]**_**2**_·0.5MeCN (both triclinic, *P*1̅, *Z* = 2). These were low-spin at 100 and 120 K, respectively, while a second structure determination of **2[BF**_**4**_**]**_**2**_·0.5MeNO_2_ confirmed that it remained low-spin at room temperature (Figures S40 and S41 and Table S14). A third measurement at 350 K led to twinning of the crystal, however, which we were unable to resolve.

**Figure 9:**
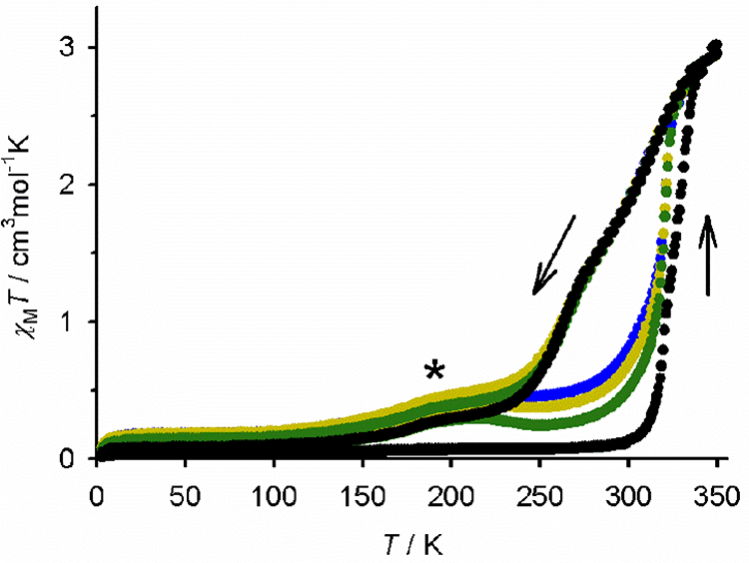
Variable-temperature magnetic susceptibility data for **2[BF**_**4**_**]**_**2**_·0.5MeNO_2_. Four consecutive thermal scans are shown (Figure S43): (i) 300 → 3 → 350 → 3 K (black); (ii) 3 → 350 → 3 K (green); (iii) 3 → 350 → 3 K (yellow); (iv) 3 → 350 → 300 K (blue). Scan rate: 5 K min^–1^. The feature marked with an asterisk grows on repeated scanning and may arise from slow desolvation of the sample as the experiment proceeds.

Variable-temperature magnetic data from **2[BF**_**4**_**]**_**2**_·0.5MeNO_2_ proved unexpectedly complicated (Figure 9). The freshly prepared compound is low-spin at 290 K, as expected, but transforms abruptly just above room temperature to a predominantly high-spin material (χ_M_*T* = 2.8 cm^3^ mol^–1^ K at 340 K). A further small increase in χ_M_*T* between 340 and 350 K implies that its SCO continues in a more gradual fashion on further heating. The high-spin → low-spin SCO upon recooling occurs gradually in three apparent steps near 340, 275, and 200 K; the material only regains its fully low-spin state below 140 K. The 200 K feature, which is marked with an asterisk in Figure 9, appears in both heating and cooling modes in scans ii–iv and slowly grows in each successive scan. The other features of the susceptibility curve are reproducible in all four scans, however.

The structural origin of this unusual behavior could not be probed in detail, because crystal structures of **[BF**_**4**_**]**_**2**_·0.5MeNO_2_ following the low-spin → high-spin transformation are unavailable. However, we postulate an abrupt crystallographic phase change from a low-spin phase A to an SCO-active phase B, on heating above 300 K. Phase B would then undergo gradual SCO on cooling, in two steps at around 340 and 275 K, and transform back to phase A at lower temperature after regaining its low-spin state. Phase B may contain two or more unique iron environments in its crystal lattice, to account for the stepwise SCO in cooling mode.^79−83^ Superimposed on this reversible behavior, the feature marked with an asterisk near 200 K may arise from partial desolvation of the sample on heating, which becomes more pronounced as the experiment proceeds. TGA data show minimal solvent loss from the material below 340 K, which is consistent with that suggestion (Figure S44).

## Conclusion

This study reports solvate compounds of [Fe(*L*^1^)_2_]X_2_ (**1X**_**2**_; X^–^ = BF_4_^–^, ClO_4_^–^). Many of the materials adopt one of three lattice types (**1X**_**2**_·Me_2_CO, **1X**_**2**_·sf, and **1X**_**2**_·MeNO_2_/**1X**_**2**_·H_2_O) exhibiting similar unit cell dimensions, but in different monoclinic space group settings. These adopt different packing motifs based on chains of [Fe(*L*^1^)_2_]^2+^ molecules linked by pairwise, intermolecular *n*···π interactions involving their disulfanyl β-S atoms (Figures S6, S16, and S30). The relationship among these structures is emphasized by the fact that **1X**_**2**_·sf is prepared from **1X**_**2**_·Me_2_CO in a single-crystal to single-crystal fashion; the transformation is so facile that it makes **1X**_**2**_·Me_2_CO difficult to characterize. Some **1X**_**2**_·MeNO_2_/**1X**_**2**_·H_2_O crystals were also converted to **1X**_**2**_·sf after more extended annealing on the diffractometer.

While not all of the intermolecular n···π contacts are notably short, they afford a large surface contact area between nearest-neighbor cations that could facilitate cooperative SCO switching. Thus, both **1X**_**2**_·Me_2_CO and **1X**_**2**_·sf exhibit abrupt thermal spin transitions at *T*_1/2_ = 150 ± 20 K, with thermal hysteresis widths of up to 38 K depending on the measurement method (Figures 1–3). However, the hysteresis widths for these compounds determined by crystallographic and magnetic measurements do not follow a consistent trend (Table 1), which implies that the solid-state kinetics^59^ and sample crystallinity^60−63^ may both contribute to the form of the transitions. All four crystals undergo a rearrangement of molecular structure between their spin states, involving a large angular displacement of their *L*^1^ ligands (Table 2). This angular rearrangement is somewhat greater for **1[BF**_**4**_**]**_**2**_·sf, whose SCO hysteresis loop is also wider than those for the other crystals.

This observation can explain the wider anion dependence of the effect of single-crystal to single-crystal desolvation of **1X**_**2**_·Me_2_CO on their SCO properties. The molecular structures of the two spin states in **1[ClO**_**4**_**]**_**2**_·Me_2_CO and **1[ClO**_**4**_**]**_**2**_·sf are very similar. However, **1[BF**_**4**_**]**_**2**_·sf undergoes a greater structural rearrangement during SCO in comparison to **1[BF**_**4**_**]**_**2**_·Me_2_CO. That larger structural change should increase the activation energy of SCO in **1[BF**_**4**_**]**_**2**_·sf, widening its hysteresis loop. Moreover, the more distorted molecular structure in the high-spin **1[BF**_**4**_**]**_**2**_·sf crystal will destabilize its low-spin state, thus lowering *T*_1/2_ as observed.^27^

Compounds adopting the third variant of this packing structure, **1X**_**2**_·MeNO_2_/**1X**_**2**_·H_2_O, exhibit more typically gradual thermal SCO equilibria centered at higher temperatures. While no high-spin crystal structures were achieved, this may imply the structure changes during SCO are smaller for this series. Notably, the less cooperative **1X**_**2**_·MeNO_2_/**1X**_**2**_·H_2_O lattice also has a higher crystal packing density in comparison to the more cooperative **1X**_**2**_·Me_2_CO. One might expect a denser crystal to exhibit more cooperative switching behavior, other things being equal, but that is not the case in this system. In fact, the literature contains examples of polymorphic or closely related SCO materials where a higher crystal density is associated with both stronger^84,85^ and weaker^86−88^ transition cooperativity.

Finally, **2[BF**_**4**_**]**_**2**_·0.5MeNO_2_ undergoes an abrupt SCO with an unusual asymmetric hysteresis loop, which is centered around room temperature and has at least two steps in its more gradual cooling branch (Figure 9). We know of only one other material whose spin-transition profile resembles that in Figure 9, but without steps on the cooling branch of the transition.^89^ Some other compounds exhibit spin transitions with more abrupt, unsymmetrical, stepped hysteresis loops.^17,35,90−95^ Where structural data are available, the asymmetry always reflects a crystallographic phase change during SCO, as proposed here.^35,89−92^ The high-spin and low-spin phases then have different lattice structures, which can lead to different transition cooperativity in the low-spin → high-spin and high-spin → low-spin processes. The forward and reverse crystallographic phase changes can also occur at different rates, especially where thermal hysteresis dictates that they take place at very different temperatures.^96,97^

This work has afforded structure–function correlations for SCO in **1X**_**2**_ solvate salts, in three related crystal lattices. The structure types exhibit similar unit cell dimensions and variations in the crystal packing motif based on chains of cations linked by pairwise intermolecular S···π contacts. Their structural similarity makes them especially valuable for determining the structural basis of cooperative phase transitions in SCO compounds and other types of functional molecular crystals.

## Supporting Information Available

Synthetic details and characterization data for the new ligand *L*^1^, experimental data, refinement procedures, and tabulated metric parameters for the crystal structure determinations, Hirshfeld fingerprint maps, X-ray powder diffraction and TGA data, and additional solid-state and solution magnetic susceptibility measurements (PDF)

## Accession Codes

CCDC 2123746–2123772 contain the supplementary crystallographic data for this paper. These data can be obtained free of charge via www.ccdc.cam.ac.uk/data_request/cif, or by emailing data_request@ccdc.cam.ac.uk, or by contacting The Cambridge Crystallographic Data Centre, 12 Union Road, Cambridge CB2 1EZ, UK; fax: +44 1223 336033.
