# Supplementary material for: Iron(II) Complexes of 4-(Alkyldisulfanyl)-2,6-di(pyrazolyl)pyridine Derivatives. Correlation of Spin-Crossover Cooperativity with Molecular Structure Following Single-Crystal-to-Single-Crystal Desolvation
Source: Cryst Growth Des. 2022 Feb 4;22(3):1960–71. doi: 10.1021/acs.cgd.2c00005 (PMC9007408; doi:10.1021/acs.cgd.2c00005)
Supplement: Supplementary file 1 — cg2c00005_si_001.pdf [file cg2c00005_si_001.pdf]

**Supporting Information**

# Iron(II) Complexes of 4-(Alkyldisulfanyl)-2,6-di(pyrazolyl)pyridine Derivatives. Correlation of Spin-Crossover Cooperativity with Molecular Structure Following Single-Crystal-to-Single-Crystal Desolvation

Rafal Kulmaczewski[a], Laurence J. Kershaw Cook[a,b], Christopher M. Pask[a], Oscar Cespedes[c] and Malcolm A. Halcrow[a,]*

[a]*School of Chemistry, University of Leeds, Woodhouse Lane, Leeds LS2 9JT, United Kingdom.*
*E-mail: m.a.halcrow@leeds.ac.uk*

[b]*Current address: Department of Chemistry, University of Liverpool, Crown Street, Liverpool L69 7ZD, UK.*

[c]*School of Physics and Astronomy, University of Leeds, EC Stoner Building, Leeds LS2 9JT, United Kingdom.*

| | | Page |
|---|---|---|
| **Experimental** | | S4 |
| **Scheme S1** | Synthesis of $L^1$. | S4 |
| **Table S1** | Experimental data for the crystal structure determinations. | S9 |
| **Figure S1** | $^{1}$H and $^{13}$C NMR spectra of $L^1$. | S14 |
| **Figure S2** | The asymmetric unit and molecular packing in the crystal structure of $L^1$. | S15 |
| **Definitions of the structural parameters discussed in the paper** | | S16 |
| **Chart S1** | Angles used in the definitions of the coordination distortion parameters $\Sigma$ and $\Theta$. | S16 |
| **Chart S2** | Definition of the Jahn-Teller distortion parameters $\theta$ and $\phi$. | S16 |
| **Figure S3** | The asymmetric unit of **1[ClO$_4$]$_2$**·Me$_2$CO, showing the full atom numbering scheme. | S17 |
| **Figure S4** | The asymmetric unit of **1[BF$_4$]$_2$**·Me$_2$CO at 250 and 143 K. | S18 |
| **Figure S5** | The asymmetric unit of **1[ClO$_4$]$_2$**·Me$_2$CO at 250 and 100 K. | S19 |
| **Table S2** | Selected bond lengths, angles and structural parameters for **1X$_2$**·Me$_2$CO. | S20 |
| **Figure S6** | Intermolecular $n\cdots\pi$ contacts in **1[BF$_4$]$_2$**·Me$_2$CO. | S21 |
| **Table S3** | Intermolecular $n\cdots\pi$ contacts in **1X$_2$**·Me$_2$CO. | S21 |
| **Table S4** | Variable temperature unit cell parameters for **1[BF$_4$]$_2$**·Me$_2$CO. | S22 |
| **Figure S7** | Variable temperature unit cell dimensions for **1[BF$_4$]$_2$**·Me$_2$CO. | S23 |
| **Figure S8** | Variable temperature unit cell volumes for **1[BF$_4$]$_2$**·Me$_2$CO. | S23 |
| **Table S5** | Variable temperature unit cell parameters for **1[ClO$_4$]$_2$**·Me$_2$CO. | S24 |
| **Figure S9** | Variable temperature unit cell dimensions for **1[ClO$_4$]$_2$**·Me$_2$CO. | S25 |
| **Figure S10** | Variable temperature unit cell volumes for **1[ClO$_4$]$_2$**·Me$_2$CO. | S25 |
| **Figure S11** | The asymmetric unit of **1[BF$_4$]$_2$**·0.75Me$_2$CO. | S26 |
| **Figure S12** | The asymmetric unit of **1[BF$_4$]$_2$**·0.5Me$_2$CO·0.5H$_2$O at 250 and 120 K. | S28 |
| **Figure S13** | The asymmetric unit of **1[ClO$_4$]$_2$**·*m*Me$_2$CO·0.5H$_2$O | S29 |
| **Table S6** | Selected bond lengths, angles and structural parameters for the other acetone solvates. | S30 |
| **Figure S14** | The asymmetric unit of **1[BF$_4$]$_2$**·sf at different temperatures. | S32 |
| **Figure S15** | The asymmetric unit of **1[ClO$_4$]$_2$**·sf at different temperatures. | S33 |
| **Table S7** | Selected bond lengths, angles and other structural parameters for **1X$_2$**·sf. | S35 |
| **Figure S16** | Intermolecular $n\cdots\pi$ contacts in **1[BF$_4$]$_2$**·sf. | S36 |
| **Table S8** | Intermolecular $n\cdots\pi$ contacts in **1X$_2$**·sf. | S37 |
| **Table S9** | Variable temperature unit cell parameters for **1[BF$_4$]$_2$**·sf. | S38 |
| **Figure S17** | Variable temperature unit cell volumes for **1[BF$_4$]$_2$**·sf. | S39 |
| **Table S10** | Variable temperature unit cell parameters for **1[ClO$_4$]$_2$**·sf. | S40 |
| **Figure S18** | Variable temperature unit cell dimensions for **1[ClO$_4$]$_2$**·sf. | S41 |
| **Figure S19** | Variable temperature unit cell volumes for **1[ClO$_4$]$_2$**·sf. | S41 |
| **Figure S20** | X-ray powder diffraction patterns for acetone solvates of **1[BF$_4$]$_2$**, and **1[BF$_4$]$_2$**·sf. | S42 |
| **Figure S21** | X-ray powder diffraction patterns for acetone solvates of **1[ClO$_4$]$_2$**, and **1[ClO$_4$]$_2$**·sf. | S43 |
| **Figure S22** | Thermogravimetric analyses for '**1[BF$_4$]$_2$**·*x*Me$_2$CO' and '**1[ClO$_4$]$_2$**·*x*Me$_2$CO'. | S44 |
| **Figure S23** | Magnetic susceptibility data for **1[BF$_4$]$_2$**·sf, at different scan rates. | S44 |
| **Figure S24** | Magnetic susceptibility data for a phase-pure sample of **1[ClO$_4$]$_2$**·*m*Me$_2$CO·0.5H$_2$O. | S46 |

| | | Page |
|---|---|---|
| **Figure S25** | Magnetic susceptibility data for a mixed-phase material ‘**1[ClO$_4$]$_2$**·$x$Me$_2$CO’. | S45 |
| **Figure S26** | The asymmetric unit of **1[BF$_4$]$_2$**·MeNO$_2$. | S47 |
| **Figure S27** | The asymmetric unit of **1[BF$_4$]$_2$**·H$_2$O. | S48 |
| **Figure S28** | The asymmetric unit of **1[ClO$_4$]$_2$**·$n$MeNO$_2$ at 250 and 120 K. | S49 |
| **Figure S29** | The asymmetric unit of **1[ClO$_4$]$_2$**·MeCN at 250 and 120 K. | S50 |
| **Table S11** | Selected bond lengths, angles and other structural parameters for the other solvate crystals. | S51 |
| **Table S12** | Hydrogen bond parameters for **1[BF$_4$]$_2$**·H$_2$O. | S52 |
| **Figure S30** | Intermolecular $n\cdots\pi$ contacts in **1[ClO$_4$]$_2$**·$n$MeNO$_2$. | S53 |
| **Table S13** | Intermolecular $n\cdots\pi$ contacts in **1[BF$_4$]$_2$**·MeNO$_2$**,** **1[BF$_4$]$_2$**·H$_2$O and **1[ClO$_4$]$_2$**·$n$MeNO$_2$. | S53 |
| **Figure S31** | TGA data for the nitromethane, acetonitrile solvate and hydrate **1X$_2$** materials. | S54 |
| **Figure S32** | X-ray powder diffraction data for **1[BF$_4$]$_2$**·solv (solv = MeNO$_2$, MeCN, H$_2$O) | S55 |
| **Figure S33** | X-ray powder diffraction data for **1[ClO$_4$]$_2$**·solv (solv = $n$MeNO$_2$, MeCN, H$_2$O) | S56 |
| **Figure S34** | Variable temperature magnetic data for the acetonitrile solvate materials. | S57 |
| **Introduction to the Hirshfeld Surface analyses** | | S58 |
| **Figure S35** | Hirshfeld fingerprint maps for **1[BF$_4$]$_2$**·Me$_2$CO and **1[ClO$_4$]$_2$**·Me$_2$CO. | S59 |
| **Figure S36** | Hirshfeld fingerprint maps for **1[BF$_4$]$_2$**·sf. | S60 |
| **Figure S37** | Hirshfeld fingerprint maps for **1[ClO$_4$]$_2$**·sf. | S61 |
| **Figure S38** | Hirshfeld fingerprint maps for the other, isomorphous solvate crystals | S62 |
| **Figure S39** | The asymmetric unit of **2[BF$_4$]$_2$**·0.5MeNO$_2$, showing the full atom numbering scheme. | S63 |
| **Figure S40** | The asymmetric unit of **2[BF$_4$]$_2$**·0.5MeNO$_2$ at 290 and 100 K. | S64 |
| **Figure S41** | The asymmetric unit of **2[BF$_4$]$_2$**·0.5MeCN. | S65 |
| **Table S14** | Selected bond lengths, angles and other structural parameters for the solvates of **2[BF$_4$]$_2$**. | S66 |
| **Figure S42** | X-ray powder diffraction data for **2[BF$_4$]$_2$**·0.5MeNO$_2$ | S66 |
| **Figure S43** | Individual plots of the multiple scans of the magnetic data for **2[BF$_4$]$_2$**·0.5MeNO$_2$. | S67 |
| **Figure S44** | Thermogravimetric analyses for **2[BF$_4$]$_2$**·0.5MeNO$_2$. | S68 |
| **Figure S45** | $^1$H NMR spectra of **1[BF$_4$]$_2$** and **2[BF$_4$]$_2$**. | S69 |
| **Figure S46** | Magnetic data for **1[BF$_4$]$_2$** and **2[BF$_4$]$_2$** in solution. | S70 |
| **Table S15** | Fitted spin-crossover parameters for **1[BF$_4$]$_2$** and **2[BF$_4$]$_2$** in solution. | S70 |
| **References** | | S71 |

## Experimental

Ligand 4-(methyldisulfanyl)-2,6-di(pyrazol-1-yl)pyridine ($L^2$)[1] and the starting material 4-mercapto-2,6-di(pyrazol-yl)pyridine[2] were prepared by our published procedures. Other reagents were purchased commercially and used as supplied. Unless otherwise stated, reactions were performed under ambient conditions using undried reagent-grade solvents.

**Synthesis of 4-(*iso*propyldisulfanyl)-2,6-di(pyrazol-1-yl)pyridine ($L^1$).** Ligand $L^1$ was obtained as a byproduct from our previously published synthesis of 4-(*iso*propylsulfanyl)-2,6-di(pyrazol-1-yl)pyridine (Scheme S1).[3] The method is described again below.

2-Iodopropane (0.18 cm$^3$, 1.80 mmol) was added to a stirred suspension of 4-mercapto-2,6-di(pyrazol-yl)pyridine (0.20 g, 0.83 mmol) and $K_2CO_3$ (0.27 g, 2.0 mmol) in MeCN (50 cm$^3$), in the presence of activated molecular sieves under an atmosphere of $N_2$. The mixture was heated to reflux for 4 hr, during which the yellow suspension became almost colourless. After cooling to room temperature, the solution was diluted to 80 cm$^3$ with $CHCl_3$, filtered, and the residue washed with additional $CHCl_3$. The filtrate and washings were combined, and all volatiles were then removed in vacuo. The pale yellow residue was taken up in aqueous $K_2CO_3$ (100 cm$^3$) and extracted with $CHCl_3$ (2x 100 cm$^3$). The organic extracts were combined, dried over $MgSO_4$, and evaporated to a yellow oil. Addition of hexane (30 cm$^3$) with stirring produced a pale yellow suspension which was filtered, yielding a solid which was identified as *bis*(2,6-di(pyrazol-1-yl)pyrid-4-yl)disulfide.[1] The yellow supernatant was subsequently evaporated to dryness, and the residue passed through a silica gel column ($CH_2Cl_2$ eluent), which yielded two products: 4-(*iso*propyl-sulfanyl)-2,6-di(pyrazol-1-yl)pyridine[2] ($R_f$ 0.45) and $L^1$ ($R_f$ 0.55). Ligand $L^1$ was isolated as a pale yellow oil which solidifies upon standing at room temperature. Yield 38 mg, 29 %. Mp 87-89 °C. ES-MS: *m*/*z* 318.0865 (calcd for $[HL^1]^+$ 318.0871), 340.0659 (calcd for $[NaL^1]^+$ 340.0667). $^1$H NMR ($CDCl_3$) δ 1.37 (d, 6.8 Hz, 6H, CH{C*H*$_3$}$_2$), 3.18 (septet, 6.9 Hz, 1H, C*H*{CH$_3$}$_2$), 6.50 (dd, J 1.7 and 2.6 Hz, 2H, Pz $H^4$), 7.78 (d, 1.7 Hz, 2H, Pz $H^3$), 8.06 (s, 2H, Py $H^{3/5}$), 8.56 (d, 2.6 Hz, 2H, Pz $H^5$) ppm. $^{13}$C NMR ($CDCl_3$) $\delta$ 22.5 (CH{*C*H$_3$}$_2$), 42.3 (*C*H{CH$_3$}$_2$), 105.6 (Py $C^{3/5}$), 108.0 (Pz $C^4$), 127.3 (Pz $C^5$), 142.5 (Pz $C^3$), 150.1 (Py $C^{2/6}$), 156.5 (Py $C^4$) ppm.

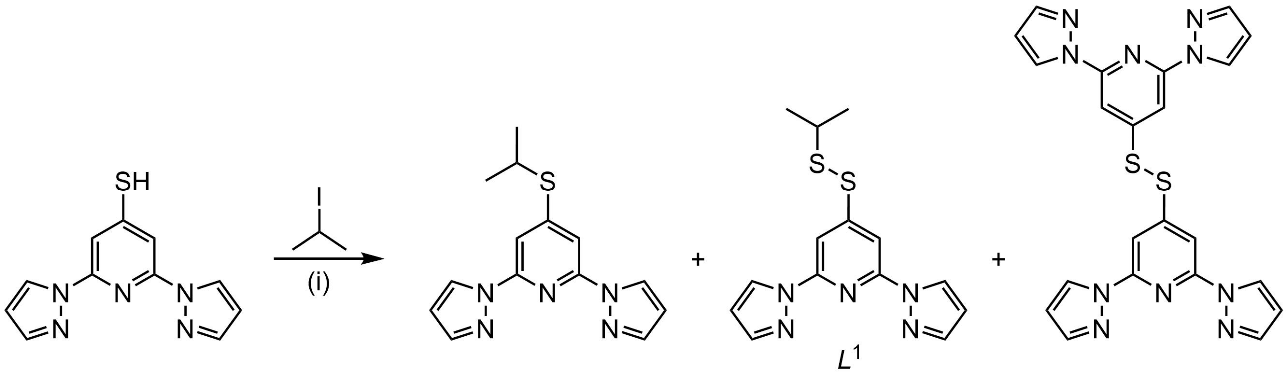


**Scheme S1** Synthesis of $L^1$. Reagents and conditions: (i) $K_2CO_3$, MeCN, reflux then $K_2CO_3$ (aq), $CHCl_3$. The same procedure using MeI as reagent was used to synthesize $L^2$.[1]

## Single Crystal Structure Analyses

Crystals of $L^1$ were grown by slow evaporation of an NMR sample of the compound in $CDCl_3$. All the complex solvate crystals were grown by slow diffusion of diethyl ether vapor into a filtered solution of the complex salt in the appropriate solvent; **1[BF$_4$]$_2$**·$H_2O$ was obtained similarly, by recrystallization from undried methanol. The **1X$_2$**·sf ($X^-$ = $BF_4^-$ and $ClO_4^-$) crystals were obtained by annealing crystals of **1X$_2$**·$Me_2CO$ on the diffractometer at 370 K for 30 mins.

All diffraction data were measured with an Agilent Supernova dual-source diffractometer using monochromated Cu-$K_\alpha$ ($\lambda$ = 1.5418 Å) radiation. The diffractometer was fitted with an Oxford Cryostream low-temperature device. Experimental details of the structure determinations in this study are given in Tables S1-S2. All the structures were solved by direct methods *(SHELXS*[4]), and developed by full least-squares refinement on $F^2$ (*SHELXL-2018*[4]). Crystallographic figures were prepared using *XSEED*,[5] and octahedral coordination volumes ($V_{Oh}$) were calculated with *Olex2*.[6]

Unless otherwise stated, the following protocols were used. All crystallographically ordered non-H atoms in these refinements were refined anisotropically. Disordered anions were modelled with refined B–F/Cl–O and F···F/O···O distance restraints; disordered *iso*propyl or *iso*propyldisulfanyl substituents were treated with fixed S–C, C–C, C···C and (where relevant) S–S restraints; and disordered lattice solvent molecules were modelled using fixed bond length and angle restraints. All C-bound H atoms were placed in calculated positions and refined using a riding model.

**Structure refinement of $L^1$.** No disorder is present in the model, and no restraints were applied to the refinement. All non-H atoms were refined anisotropically. All H atoms were located in the Fourier map and refined positionally, with $U_{iso}$ constrained to 1.2x $U_{eq}$ for the corresponding C atom ($sp^2$ and tertiary C–H), or 1.5x $U_{eq}$ (methyl C–H).

**Structure refinements of 1[BF$_4$]$_2$·Me$_2$CO**. Two datasets were collected from the same crystal at 143 and 250 K. The asymmetric unit contains one formula unit of the compound, with all residues on general crystallographic sites.

No disorder is present at 143 K, and no restraints were applied to that refinement. The highest residual Fourier peak of +1.1 $e$ Å$^{-3}$ is 1.0 Å from Fe(1).

At 250 K, the following residues are disordered. *Iso*propyldisulfanyl group S(39)-C(42) is disordered over two sites, with refined occupancies of 0.71:0.29. Both anions are also disordered over two sites. The disorder occupancies for B(44)-F(48) refined to 0.71:0.29, the same as for the SS*i*Pr group, but the disorder sites for B(49)-F(53) were refined as equally occupied. Lastly, the acetone molecule is badly disordered and was refined over three equally occupied sites, two of which share a common central C atom C(55A). All wholly occupied non-H atoms, plus the partial heavy atoms S(39A) and S(40A), were refined anisotropically.

**Structure refinements of 1[ClO$_4$]$_2$·Me$_2$CO**. Two datasets were collected from the same crystal at 100 and 250 K. The crystal is isomorphous with the $BF_4^-$ salt of this solvate, and contains one formula unit of the compound.

No disorder is present at 100 K, and no restraints were applied to that refinement.

The following residues are disordered at 250 K. Isopropyldisulfanyl group S(39)-C(42) is disordered over two sites, with refined occupancies of 0.67:0.33. Perchlorate ion Cl(49)-O(53) is also disordered over two orientations. Lastly, the acetone molecule is badly disordered and was refined over three equally occupied sites, two of which share a common central C atom C(55A). All wholly occupied non-H atoms, plus the partial heavy atoms S(39A), S(40A), Cl(49A) and Cl(49B), were refined anisotropically.

**Structure refinements of 1[BF$_4$]$_2$·0.5Me$_2$CO·0.5H$_2$O**. Two datasets were collected from the same crystal at 120 and 250 K. The asymmetric unit contains two complex dications, two half-tetrafluoroborate anions spanning crystallographic $C_2$ axes; three other whole $BF_4^-$ ions on general crystallographic sites; one molecule of acetone, and a disordered region that was modelled as a disordered molecule of water.

All three of the whole $BF_4^-$ ions are disordered at 120 K, over two sites whose occupancies each refined to 0.70:0.30 +/-0.02. All non-H atoms with occupancies >0.5 were modelled anisotropically.

At 250 K, the whole $BF_4^-$ ions are modelled over three equally occupied disorder sites, two of which share a common 0.67-occupied B atom. Two *iso*propyl groups are also modelled over two orientations, with occupancies of 0.50:0.50 or 0.67:0.33. Lastly, the acetone molecule is disordered over two sites which share a wholly occupied central C atom.

The disordered water H atoms were not apparent in the Fourier maps and are not included in the refinements, but are accounted for in the formula of the crystal and the density calculations.

**Structure refinements of 1[BF$_4$]$_2$·0.75Me$_2$CO**. Two datasets were collected from the same crystal at 120 and 250 K. The asymmetric unit contains two formula units of the compound; that is, two complex dications, four tetrafluorobrate anions; one whole molecule of acetone; and a half-molecule of acetone spanning a crystallographic inversion center.

At 120 K, consistently high displacement ellipsoids on ligand N(23B)-C(43B) in complex molecule 'B' were interpreted as disorder, which was modelled over two sites with refined occupancies of 0.74:0.26. This disorder was also extended to Fe(1B), to give more reasonable Fe–N bond lengths to the ligand disorder orientations. The disordered heterocycle core was modelled without restraints, but the fixed restraints listed above were applied to the *iso*propyldisulfanyl substituents. The F atoms of one of the $BF_4^-$ are also disordered, over two equally occupied orientations. All crystallographically occupied non-H atoms, plus the major orientations of the disordered S atoms, were modelled anisotropically.

At 250 K the heterocyclic ligand disorder in cation B is no longer evident, but the *iso*propyldisulfanyl group disorder is still present and was modelled over three equally occupied orientations. All four anions are also disordered, three of them over two equally occupied orientations and the fourth over three sites that share a common B atom.

Both refinements yield a highest residual Fourier peak of +1.3-1.5 $e$ Å$^{-3}$, which lies within the disordered *iso*propyldisulfanyl group.

**Structure refinements of 1[ClO$_4$]$_2$·*m*Me$_2$CO·0.5H$_2$O (*m* ≈ 0.34)**. This crystal is not isomorphous with **1[BF$_4$]**$_2$·0.5Me$_2$CO·0.5H$_2$O. Its asymmetric unit contains two complex dications, four perchlorate anions, one molecule of acetone which is ordered but only *ca.* two-thirds occupied, and one molecule of water. All these residues lie on general crystallographic sites.

A full refinement of this crystal was obtained at 120 K. The isopropyldisulfanyl group S(18B)-C(22B) is disordered over three sites with an occupancy ratio of 0.50:0.30:0.20. Two of the four perchlorate ions are also disordered, one over two equally occupied orientations and the other over three sites with occupancies of 0.67:0.20:0.13. Although the acetone molecule is ordered, fixed distance restraints were required for this residue to attain a sensible geometry. All wholly occupied non-H atoms, S and Cl atoms with occupancies ≥0.5, and the partial acetone site, were modelled anisotropically. The water H atoms were not apparent in the Fourier maps and are not included in the refinements, but are accounted for in the formula of the crystal and the density calculation. The highest residual Fourier peak of +1.7 $e$ Å$^{-3}$ is 0.7 Å from Fe(1A).

A second dataset was collected from the same crystal at 250 K. It retained the same unit cell and space group at this temperature, but the dataset had a high mosaicity. A preliminary solution showed all four anions and at least two S*i*Pr groups were disordered, and the occupancy of the lattice solvent sites was reduced. A full refinement was not achieved from this dataset, and this structure has not been deposited with the CCDC.

**Structure refinements of 1[BF$_4$]$_2$·sf**. All the datasets of this compound were obtained from the same crystal. The compound is high-spin at 250 K, and the crystal retained its high-spin state the first time it was cooled to 100 K on the diffractometer. Rewarming the crystal to 250 K, then recooling it under nominally the same conditions, then afforded the crystal in its low-spin state. This behavior is consistent with the magnetic data from this material, which imply its thermal SCO occurs slowly on cooling below 140 K with a significant kinetic barrier. The different outcomes of the two experiments might be caused by small differences in the temperature ramp in the two experiments. Alternatively, they could reflect the introduction of additional defects or a reduction in domain size in the crystal following the first thermal cycle.

The asymmetric unit contains one formula unit of the compound, with all residues on general crystallographic sites. No disorder is present in either of the low temperature refinements.

Both 250 K refinements include disorder at one *iso*propyl group, over two sites with refined occupancies of 0.61:0.39. Both anions are also disordered over three orientations. Attempts to model disorder in the other *iso*propyl group were unsuccessful, despite its slightly high displacement ellipsoids. All crystallographically ordered non-H atoms with occupancy >0.5 were refined anisotropically.

**Structure refinements of 1[ClO$_4$]$_2$·sf**. This compound is isomorphous with solvent-free **1[BF$_4$]$_2$**. The crystal was measured four times at temperatures spanning the hysteretic spin-transition in its magnetic data, at 250, 170 (cooling), 110 and 170 K (warming) in that order.

No disorder is present in the crystal at 110 K, and no restraints were applied to that refinement.

The crystal was characterized in both its high-spin and low-spin states at 170 K. The high-spin crystal has disorder in *iso*propyldisulfanyl group S(18)-C(22), which was modelled over three equally occupied sites. Both perchlorate ions are also disordered over three sites with equal occupancies. As well as the refined Cl–O and O···O distance restraints mentioned above, an antibumping restraint between the cation and one anion disorder site was also required to ensure a sensible geometry. This anion disorder is quenched in the low-spin form however, while the disordered *iso*propylsulfanyl group was refined over just two half-occupied sites which share a common wholly occupied atom S(18).

The 250 K refinement retains the same ligand and anion disorder as in the high-spin 170 K structure, and was treated in the same way.

**Structure refinement of 1[BF$_4$]$_2$·MeNO$_2$**. This compound was measured at 120 K. Both $BF_4^-$ ions are disordered. One of these was modelled over three complete sites with a 0.4:0.3:0.3 occupancy ratio, while the other was refined over three sites with occupancies of 0.5:0.3:0.2, which share a common wholly occupied B atom (although the latter anion sites deviate significantly from tetrahedrality, attempts to refine separate B atoms for each partial anion were unsuccessful). The *iso*propylsulfanyl group S(40)-C(43) is also disordered over two orientations, with refined occupancies of 0.60:0.04. Lastly, the nitromethane molecule was refined over two disorder orientations whose occupancies refined to 0.70:0.30. All fully occupied non-H atoms, plus the partial S atoms S(40A) and S(40B), were refined anisotropically. The highest residual Fourier peak of +1.8 *e* Å$^{-3}$ is associated with one of the anion disorder sites.

**Structure refinement of 1[BF$_4$]$_2$·H$_2$O**. This crystal is isomorphous with **1[BF$_4$]$_2$**·MeNO$_2$, and was measured at 120 K. One $BF_4^-$ ion is disordered, and was modelled over three sites with occupancies of 0.4. 0.4 and 0.2. *Iso*propyl group C(20)-C(22) is also disordered over two sites, whose occupancy ratio refined to 0.73:0.27. All crystallographically ordered non-H atoms, plus the major isopropyl disorder orientation, were refined anisotropically. The water H atoms were loctaed in the Fourier map and refined subject to the fixed restraints O−H = 0.90(2) and H···H = 1.47(2) Å, and with $U_{iso}$ constrained to 1.5x $U_{eq}$ for the attached O atom. The highest residual Fourier peak of +1.3 Å$^{-3}$ lies within the disordered anion.

**Structure refinements of 1[ClO$_4$]$_2$·*n*MeNO$_2$ (*m* ≈ 0.90)**. This crystal is isomorphous with **1X$_2$**·Me$_2$CO ($X^-$ = $BF_4^-$ and $ClO_4^-$) and with **1[BF$_4$]$_2$**·MeNO$_2$. It was measured at two temperatures, 120 and 250 K.

After an initial refinement of the 120 K structure, a large residual Fourier peak was incorporated into the model as a minor, 0.1-occupied disorder site for perchlorate ion Cl(49)-O(53). The close contact O(53B) ···O(57) = 1.58 Å implies that the minor anion site and the nitromethane molecule cannot be simultaneously occupied. Since a minor solvent disorder site was not apparent, the occupancy of the nitromethane molecule was reduced to 0.9 to account for this. All non-H atoms except the minor anion disorder site were refined anisotropically.

Both $ClO_4^-$ ions are disordered at 250 K. One of these was modelled over three complete sites with a 0.4:0.3:0.3 occupancy ratio, while the other was refined over three sites with occupancies of 0.6:0.2:0.2. The isopropylsulfanyl group S(40)-C(43) is also disordered over two orientations, which were modelled as equally occupied. Lastly, the nitromethane molecule was refined over three disorder orientations whose occupancies sum to 0.9 (the same solvent content used in the refinement of this crystal at 120 K, where the solvent is ordered). All fully occupied non-H atoms with occupancy >0.5, plus the disordered S atoms, were refined anisotropically.

**Structure refinements of 1[ClO$_4$]$_2$·MeCN**. This crystal is not isomorphous with the acetone or nitromethane solvates. Its asymmetric unit contains two complex dications, four perchlorate anions and two molecules of acetonitrile, all on general crystallographic sites. The crystal was measured at 120 and 250 K.

No disorder is present at 120 K, and no restraints were applied to that refinement.

At 250 K, *iso*propyl group C(20A)-C(22A) is disordered over two equally occupied sites. Two of the perchlorate ions are also disordered, over two or three equally occupied orientations respectively. Lastly, one acetonitrile molecule C67-N69 was refined over two half-occupied orientations. All wholly occupied non-H atoms, plus the half-occupied Cl atoms Cl(44A) and Cl(44B), were refined anisotropically.

**Structure refinement of 2[BF$_4$]$_2$·0.5MeNO$_2$**. Full refinements were achieved at 100 and 290 K, from the same crystal of this compound. The asymmetric unit contains one complex dication and one $BF_4^-$ ion on general crystallographic sites; a half $BF_4^-$ ion that spans the inversion center at 0, ½, 0; and, a disordered region on a general site near the inversion center at 1, 0, 0 that was modelled as the remaining half-anion, and an overlapping half-molecule of nitromethane. The latter residues occupy a cavity near that inversion center containining one anion and one solvent molecule, which are disordered about that inversion center through the lattice.

No disorder was included in the refinement at 100 K, and all non-H atoms except for the overlapping anion/solvent site were refined anisotropically.

At 290 K, all the anion sites and the partial solvent molecule were disordered. Each residue was modelled over two equally occupied orientations, with the two components of the disordered half-anion sites each sharing a common half-occupied B atom. All fully occupied non-H atoms were refined anisotropically.

An attempted third data collection at 350 K from this crystal was unsuccessful. The crystal had become twinned at this temperature, which could reflect a phase change associated with its spin-transition on warming. The alternative explanation, of crystal decomposition from solvent loss, is less likely on the basis of its magnetic data (Figure 9, main article, and Figure S43).

**Structure refinement of 2[BF$_4$]$_2$·0.5MeCN**. This crystal was measured at 120 K, where it is isomorphous with the nitromethane solvate of the compound. The same protocol was used as for the low-temperature refinement of that crystal, except that no restraints were required to successfully model the anion component of the overlapping anion/solvent site.

CCDC 2123746-2123772 contain the supplementary crystallographic data for this paper (Table S1). These data can be obtained free of charge from The Cambridge Crystallographic Data Center via www.ccdc.cam.ac.uk/data_request/cif.

**Table S1** Experimental data for the crystal structures in this work.

| | $L^1$ | **1[BF$_4$]$_2$**·Me$_2$CO | | **1[ClO$_4$]$_2$**·Me$_2$CO | |
|---|---|---|---|---|---|
| molecular formula | $C_{14}H_{15}N_5S_2$ | $C_{31}H_{36}B_2F_8FeN_{10}OS_4$ | | $C_{31}H_{36}Cl_2FeN_{10}O_9S_4$ | |
| $M_r$ | 317.43 | 922.41 | | 947.69 | |
| crystal class | orthorhombic | monoclinic | | monoclinic | |
| space group | *Pbca* | $P2_1/c$ | | $P2_1/c$ | |
| $a$ / Å | 9.76252(13) | 12.1943(2) | 12.7169(2) | 11.9704(1) | 12.4744(2) |
| $b$ / Å | 16.3359(2) | 16.8584(2) | 17.0491(3) | 17.0136(2) | 17.2699(3) |
| $c$ / Å | 18.5892(2) | 19.6797(2) | 19.4584(3) | 20.0001(2) | 19.8927(3) |
| $\alpha$ / ° | 90 | 90 | 90 | 90 | 90 |
| $\beta$ / ° | 90 | 104.496(1) | 105.211(2) | 104.057(1) | 105.507(2) |
| $\gamma$ / ° | 90 | 90 | 90 | 90 | 90 |
| $V$ / Å$^3$ | 2964.59(7) | 3916.89(9) | 4071.01(12) | 3951.24(7) | 4129.51(12) |
| $Z$ | 8 | 4 | 4 | 4 | 4 |
| $T$ / K | 120(2) | 143(2) | 250(2) | 100(2) | 250(2) |
| $\mu\{Cu\text{-}K_\alpha\}$ / mm$^{-1}$ | 3.258 | 5.800 | 5.580 | 6.847 | 6.552 |
| $D_c$ / gcm$^{-3}$ | 1.422 | 1.564 | 1.505 | 1.593 | 1.524 |
| measured reflections | 11929 | 15722 | 16505 | 15135 | 13883 |
| independent reflections | 2937 | 7538 | 7855 | 7053 | 7977 |
| $R_{int}$ | 0.033 | 0.038 | 0.035 | 0.041 | 0.037 |
| parameters | 235 | 518 | 520 | 518 | 535 |
| restraints | 0 | 0 | 62 | 0 | 50 |
| $R_1$ [$F_0 > 4\sigma(F_0)$][a] | 0.036 | 0.051 | 0.069 | 0.040 | 0.062 |
| $wR_2$, all data[b] | 0.101 | 0.140 | 0.205 | 0.111 | 0.185 |
| goodness of fit | 1.036 | 1.020 | 1.048 | 1.017 | 1.024 |
| $\Delta\rho_{min/max}$ / $e$Å$^{-3}$ | –0.33/0.37 | –0.62/1.11 | –0.69/0.81 | –0.47/0.67 | –0.49/0.60 |
| CCDC | 2123746 | 2123750 | 2123749 | 2123747 | 2123748 |

[a]$R = \Sigma[|F_o| - |F_c|] / \Sigma|F_o|$ [b]$wR = [\Sigma w(F_o^2 - F_c^2) / \Sigma wF_o^4]^{1/2}$ [c]The 250 K dataset of this crystal had a high mosaicity, and a preliminary solution showed it to be highly disordered. A publishable refinement of that dataset was not achieved, and it has not been deposited with the CCDC. [d]The crystal remained in its high-spin state the first time it was cooled to 100 K on the diffractometer. [e]The same crystal transformed to its low-spin state when it was rewarmed, then cooled to 100 K a second time.

**Table S1** continued.

| | $\mathbf{1[BF_4]_2}\cdot 0.75Me_2CO$ | | $\mathbf{1[BF_4]_2}\cdot 0.5Me_2CO\cdot 0.5H_2O$ | | $\mathbf{1[ClO_4]_2}\cdot m Me_2CO\cdot 0.5H_2O$ ($m \approx 0.34$)[c] | |
|---|---|---|---|---|---|---|
| molecular formula | $C_{30.25}H_{34.50}B_2F_8FeN_{10}O_{0.75}S_4$ | | $C_{29.50}H_{34}B_2F_8FeN_{10}OS_4$ | | $C_{29.02}H_{33.04}Cl_2FeN_{10}O_{8.84}S_4$ | |
| $M_r$ | 907.89 | | 902.37 | | 918.37 | |
| crystal class | triclinic | | monoclinic | | monoclinic | |
| space group | $P\bar{1}$ | | $P2/c$ | | $P2_1/c$ | |
| $a$ / Å | 12.8889(3) | 12.9621(3) | 25.2402(3) | 25.6296(8) | 19.9721(4) | 20.0674(5) |
| $b$ / Å | 13.3259(2) | 13.4851(2) | 8.4486(1) | 8.4917(2) | 16.5811(3) | 16.7466(4) |
| $c$ / Å | 23.6218(5) | 23.8350(5) | 37.2231(4) | 37.3752(9) | 24.8338(5) | 25.0930(8) |
| $\alpha$ / ° | 81.096(2) | 81.944(2) | 90 | 90 | 90 | 90 |
| $\beta$ / ° | 79.288(2) | 79.326(2) | 101.297(1) | 101.641(3) | 111.790(2) | 111.968(3) |
| $\gamma$ / ° | 88.153(2) | 87.878(2) | 90 | 90 | 90 | 90 |
| $V$ / Å$^3$ | 3938.42(14) | 4053.49(14) | 7783.82(16) | 7966.9(4) | 7636.4(3) | 7820.5(4) |
| $Z$ | 4 | 4 | 8 | 8 | 8 | 8 |
| $T$ / K | 120(2) | 250(2) | 120(2) | 250(2) | 120(2) | 250(2) |
| $\mu$\{Cu-$K_\alpha$\} / mm$^{-1}$ | 5.753 | 5.590 | 5.823 | 5.689 | 7.061 | – |
| $D_c$ / gcm$^{-3}$ | 1.531 | 1.488 | 1.540 | 1.505 | 1.597 | – |
| measured reflections | 33731 | 34791 | 30774 | 30958 | 35921 | – |
| independent reflections | 13602 | 13991 | 15276 | 15544 | 14593 | – |
| $R_{int}$ | 0.023 | 0.029 | 0.043 | 0.056 | 0.046 | – |
| parameters | 1019 | 1040 | 1075 | 1033 | 1057 | – |
| restraints | 32 | 116 | 60 | 121 | 74 | – |
| $R_1$ [$F_0 > 4\sigma(F_0)$][a] | 0.044 | 0.066 | 0.069 | 0.084 | 0.086 | – |
| $wR_2$, all data[b] | 0.113 | 0.189 | 0.186 | 0.249 | 0.235 | – |
| goodness of fit | 1.032 | 1.082 | 1.032 | 1.028 | 1.059 | – |
| $\Delta\rho_{min/max}$ / $e$Å$^{-3}$ | –0.85/1.52 | –1.10/1.24 | –0.89/0.90 | –0.66/0.68 | –0.78/1.40 | – |
| CCDC | 2123753 | 2123754 | 2123752 | 2123751 | 2123755 | – |

[a]$R = \Sigma[|F_o| - |F_c|] / \Sigma|F_o|$ [b]$wR = [\Sigma w(F_o^2 - F_c^2) / \Sigma wF_o^4]^{1/2}$ [c]The 250 K dataset of this crystal had a high mosaicity, and a preliminary solution showed it to be highly disordered. A publishable refinement of that dataset was not achieved, and it has not been deposited with the CCDC. [d]The crystal remained in its high-spin state the first time it was cooled to 100 K on the diffractometer. [e]The same crystal transformed to its low-spin state when it was rewarmed, then cooled to 100 K a second time.

**Table S1** continued.

| | **1[BF$_4$]$_2$**·sf (experiment 1)[d] | | **1[BF$_4$]$_2$**·sf (experiment 2)[e] | | **1[ClO$_4$]$_2$**·sf | | | |
|---|---|---|---|---|---|---|---|---|
| molecular formula | $C_{28}H_{30}B_2F_8FeN_{10}S_4$ | | $C_{28}H_{30}B_2F_8FeN_{10}S_4$ | | $C_{28}H_{30}Cl_2FeN_{10}O_8S_4$ | | | |
| $M_r$ | 864.33 | | 864.33 | | 889.61 | | | |
| crystal class | monoclinic | | monoclinic | | monoclinic | | | |
| space group | $P2_1/n$ | | $P2_1/n$ | | $P2_1/n$ | | | |
| $a$ / Å | 12.58782(15) | 12.7196(2) | 12.2754(3) | 12.7080(3) | 12.4030(2) | 12.4226(2) | 12.7092(4) | 12.8000(3) |
| $b$ / Å | 18.7203(2) | 18.8408(3) | 19.0782(3) | 18.8533(3) | 19.3039(3) | 19.3128(3) | 19.0565(5) | 19.0750(4) |
| $c$ / Å | 15.8938(2) | 16.0708(3) | 15.9164(3) | 16.0715(4) | 16.0229(3) | 16.0603(3) | 16.0778(5) | 16.1822(4) |
| $\alpha$ / ° | 90 | 90 | 90 | 90 | 90 | 90 | 90 | 90 |
| $\beta$ / ° | 100.8847(12) | 101.270(2) | 103.796(2) | 101.306(2) | 103.862(2) | 103.857(2)) | 102.225(3) | 102.365(3) |
| $\gamma$ / ° | 90 | 90 | 90 | 90 | 90 | 90 | 90 | 90 |
| $V$ / Å$^3$ | 3677.94(8) | 3777.06(11) | 3619.97(12) | 3775.81(15) | 3724.57(11) | 3740.97(11) | 3805.6(2) | 3859.39(16) |
| $Z$ | 4 | 4 | 4 | 4 | 4 | 4 | 4 | 4 |
| $T$ / K | 100(2) | 250(2) | 100(2) | 250(2) | 110(2) | 170(2) (warming) | 170(2) (cooling) | 250(2) |
| $\mu$\{Cu-$K_\alpha$\} / mm$^{-1}$ | 6.114 | 5.953 | 6.212 | 5.955 | 7.202 | 7.170 | 7.048 | 6.950 |
| $D_c$ / gcm$^{-3}$ | 1.561 | 1.520 | 1.586 | 1.520 | 1.586 | 1.580 | 1.553 | 1.531 |
| measured reflections | 15205 | 15069 | 13902 | 12028 | 15062 | 15176 | 15433 | 16936 |
| independent reflections | 7222 | 7431 | 7082 | 6662 | 7119 | 7147 | 7278 | 7448 |
| $R_{int}$ | 0.039 | 0.032 | 0.050 | 0.046 | 0.041 | 0.043 | 0.046 | 0.034 |
| parameters | 482 | 509 | 482 | 505 | 482 | 476 | 526 | 526 |
| restraints | 0 | 70 | 0 | 68 | 0 | 10 | 79 | 79 |
| $R_1$ [$F_0 > 4\sigma(F_0)$][a] | 0.044 | 0.059 | 0.060 | 0.071 | 0.053 | 0.059 | 0.066 | 0.064 |
| $wR_2$, all data[b] | 0.117 | 0.172 | 0.168 | 0.202 | 0.148 | 0.158 | 0.171 | 0.186 |
| goodness of fit | 1.018 | 1.076 | 1.061 | 1.028 | 1.034 | 1.030 | 1.034 | 1.059 |
| $\Delta\rho_{min/max}$ / $e$Å$^{-3}$ | –0.49/0.60 | –0.51/0.75 | –0.55/1.09 | –0.54/0.71 | –0.50/0.86 | –0.95/0.94 | –0.54/0.73 | –0.63/0.83 |
| CCDC | 2123759 | 2123760 | 2123761 | 2123762 | 2123756 | 2123757 | 2123758 | 2123763 |

[a]$R = \Sigma[|F_o| - |F_c|] / \Sigma|F_o|$ [b]$wR = [\Sigma w(F_o^2 - F_c^2) / \Sigma wF_o^4]^{1/2}$ [c]The 250 K dataset of this crystal had a high mosaicity, and a preliminary solution showed it to be highly disordered. A publishable refinement of that dataset was not achieved, and it has not been deposited with the CCDC. [d]The crystal remained in its high-spin state the first time it was cooled to 100 K on the diffractometer. [e]The same crystal transformed to its low-spin state when it was rewarmed, then cooled to 100 K a second time.

**Table S1** continued.

| | **1[BF$_4$]$_2$**·MeNO$_2$ | **1[BF$_4$]$_2$**·H$_2$O | **1[ClO$_4$]$_2$**·*n*MeNO$_2$ ($n \approx 0.90$) | | **1[ClO$_4$]$_2$**·MeCN | |
|---|---|---|---|---|---|---|
| molecular formula | $C_{29}H_{33}B_2F_8FeN_{11}O_2S_4$ | $C_{28}H_{32}B_2F_8FeN_{10}OS_4$ | $C_{28.90}H_{32.70}Cl_2FeN_{10.90}O_{9.80}S_4$ | | $C_{30}H_{33}Cl_2FeN_{11}O_8S_4$ | |
| $M_r$ | 925.37 | 882.34 | 944.55 | | 930.66 | |
| crystal class | monoclinic | monoclinic | monoclinic | | triclinic | |
| space group | $P2_1/n$ | $P2_1/n$ | $P2_1/n$ | | $P\bar{1}$ | |
| $a$ / Å | 12.9685(3) | 12.7457(2) | 12.8600(2) | 13.0821(2) | 8.7133(2) | 8.9802(4) |
| $b$ / Å | 16.8233(3) | 16.4226(3) | 16.9317(2) | 17.1004(3) | 20.8463(5) | 21.0508(4) |
| $c$ / Å | 18.3207(5) | 18.5464(3) | 18.3511(2) | 18.4896(3) | 21.8709(6) | 21.8430(8) |
| $\alpha$ / ° | 90 | 90 | 90 | 90 | 79.380(2) | 78.692(3) |
| $\beta$ / ° | 104.345(2) | 104.900(2) | 103.382(1) | 103.8318(16) | 87.267(2) | 87.797(3) |
| $\gamma$ / ° | 90 | 90 | 90 | 90 | 88.154(2) | 88.952(3) |
| $V$ / Å$^3$ | 3872.48(15) | 3751.56(11) | 3887.31(9) | 4016.35(11) | 3899.03(17) | 4045.8(2) |
| $Z$ | 4 | 4 | 4 | 4 | 4 | 4 |
| $T$ / K | 120(2) | 120(2) | 120(2) | 250(2) | 120(2) | 250(2) |
| $\mu$\{Cu-$K_\alpha$\} / mm$^{-1}$ | 5.897 | 6.026 | 6.981 | 6.757 | 6.916 | 6.665 |
| $D_c$ / gcm$^{-3}$ | 1.587 | 1.562 | 1.614 | 1.562 | 1.585 | 1.528 |
| measured reflections | 16581 | 15600 | 15648 | 13571 | 22245 | 32583 |
| independent reflections | 7402 | 7366 | 7631 | 7747 | 11575 | 13855 |
| $R_{int}$ | 0.044 | 0.031 | 0.040 | 0.034 | 0.037 | 0.060 |
| parameters | 541 | 527 | 537 | 579 | 1019 | 1033 |
| restraints | 79 | 41 | 20 | 90 | 0 | 66 |
| $R_1$ [$F_0 > 4\sigma(F_0)$][a] | 0.065 | 0.052 | 0.058 | 0.072 | 0.042 | 0.068 |
| $wR_2$, all data[b] | 0.186 | 0.142 | 0.163 | 0.215 | 0.110 | 0.206 |
| goodness of fit | 1.062 | 1.038 | 1.029 | 1.049 | 1.029 | 1.032 |
| $\Delta\rho_{min/max}$ / $e$Å$^{-3}$ | –0.64/1.78 | –0.50/1.32 | –0.70/1.18 | –0.60/0.81 | –0.36/0.49 | –0.48/0.68 |
| CCDC | 2123766 | 2123764 | 2123765 | 2123769 | 2123768 | 2123767 |

[a]$R = \Sigma[|F_o| - |F_c|] / \Sigma|F_o|$ [b]$wR = [\Sigma w(F_o^2 - F_c^2) / \Sigma wF_o^4]^{1/2}$ [c]The 250 K dataset of this crystal had a high mosaicity, and a preliminary solution showed it to be highly disordered. A publishable refinement of that dataset was not achieved, and it has not been deposited with the CCDC. [d]The crystal remained in its high-spin state the first time it was cooled to 100 K on the diffractometer. [e]The same crystal transformed to its low-spin state when it was rewarmed, then cooled to 100 K a second time.

**Table S1** continued.

| | $\mathbf{2[BF_4]_2}\cdot 0.5MeNO_2$ | | $\mathbf{2[BF_4]_2}\cdot 0.5MeCN$ |
|---|---|---|---|
| molecular formula | $C_{24.50}H_{23.50}B_2F_8FeN_{10.50}OS_4$ | | $C_{25}H_{23.50}B_2F_8FeN_{10.50}S_4$ |
| $M_r$ | 838.75 | | 828.75 |
| crystal class | triclinic | | triclinic |
| space group | $P\bar{1}$ | | $P\bar{1}$ |
| $a$ / Å | 8.5708(2) | 8.6911(5) | 8.5781(2) |
| $b$ / Å | 11.7468(3) | 11.9097(9) | 11.7788(4) |
| $c$ / Å | 17.9514(4) | 18.0142(12) | 17.9031(6) |
| $\alpha$ / ° | 81.201(2) | 80.944(6) | 80.540(3) |
| $\beta$ / ° | 89.051(2) | 89.107(5) | 89.785(3) |
| $\gamma$ / ° | 70.218(3) | 70.287(6) | 70.987(3) |
| $V$ / Å$^3$ | 1679.46(8) | 1732.1(2) | 1684.57(10) |
| $Z$ | 2 | 2 | 2 |
| $T$ / K | 100(2) | 290(2) | 120(2) |
| $\mu\{\text{Cu-}K_\alpha\}$ / mm$^{-1}$ | 6.703 | 6.499 | 6.652 |
| $D_c$ / gcm$^{-3}$ | 1.659 | 1.608 | 1.634 |
| measured reflections | 13079 | 13367 | 11691 |
| independent reflections | 6358 | 6553 | 6352 |
| $R_{int}$ | 0.024 | 0.035 | 0.032 |
| parameters | 481 | 499 | 477 |
| restraints | 16 | 72 | 3 |
| $R_1$ [$F_0 > 4\sigma(F_0)$][a] | 0.042 | 0.068 | 0.045 |
| $wR_2$, all data[b] | 0.105 | 0.190 | 0.117 |
| goodness of fit | 1.049 | 1.056 | 1.041 |
| $\Delta\rho_{min/max}$ / $e$Å$^{-3}$ | –1.00/0.86 | –0.58/0.95 | –0.60/0.78 |
| CCDC | 2123770 | 2123771 | 2123772 |

[a]$R = \Sigma[|F_o| - |F_c|] / \Sigma|F_o|$ [b]$wR = [\Sigma w(F_o^2 - F_c^2) / \Sigma wF_o^4]^{1/2}$ [c]The 250 K dataset of this crystal had a high mosaicity, and a preliminary solution showed it to be highly disordered. A publishable refinement of that dataset was not achieved, and it has not been deposited with the CCDC. [d]The crystal remained in its high-spin state the first time it was cooled to 100 K on the diffractometer. [e]The same crystal transformed to its low-spin state when it was rewarmed, then cooled to 100 K a second time.

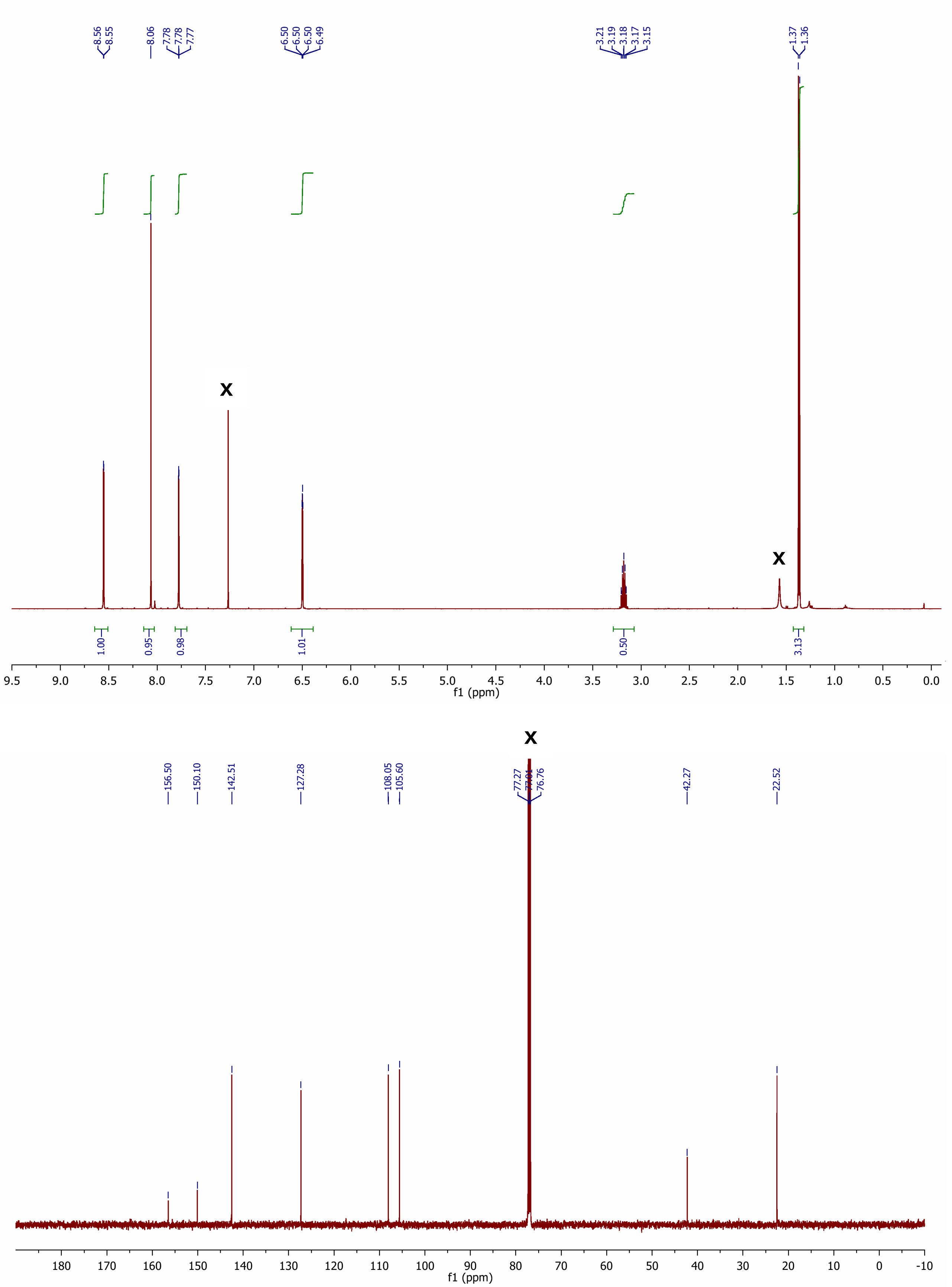


**Figure S1** $^{1}$H (top) and $^{13}$C (bottom) NMR spectra of the new ligand $L^1$ ($CDCl_3$).

Disulfide $L^1$ is most readily distinguished from its coproduct 4-(*iso*propylsulfanyl)-2,6-di(pyrazol-1-yl)-pyridine (Scheme S1) by the Py $H^{3/5}$ singlet proton resonance, which occurs at 8.06 ppm in $L^1$ and at 7.73 ppm in the monosulfide in $CDCl_3$.[3]

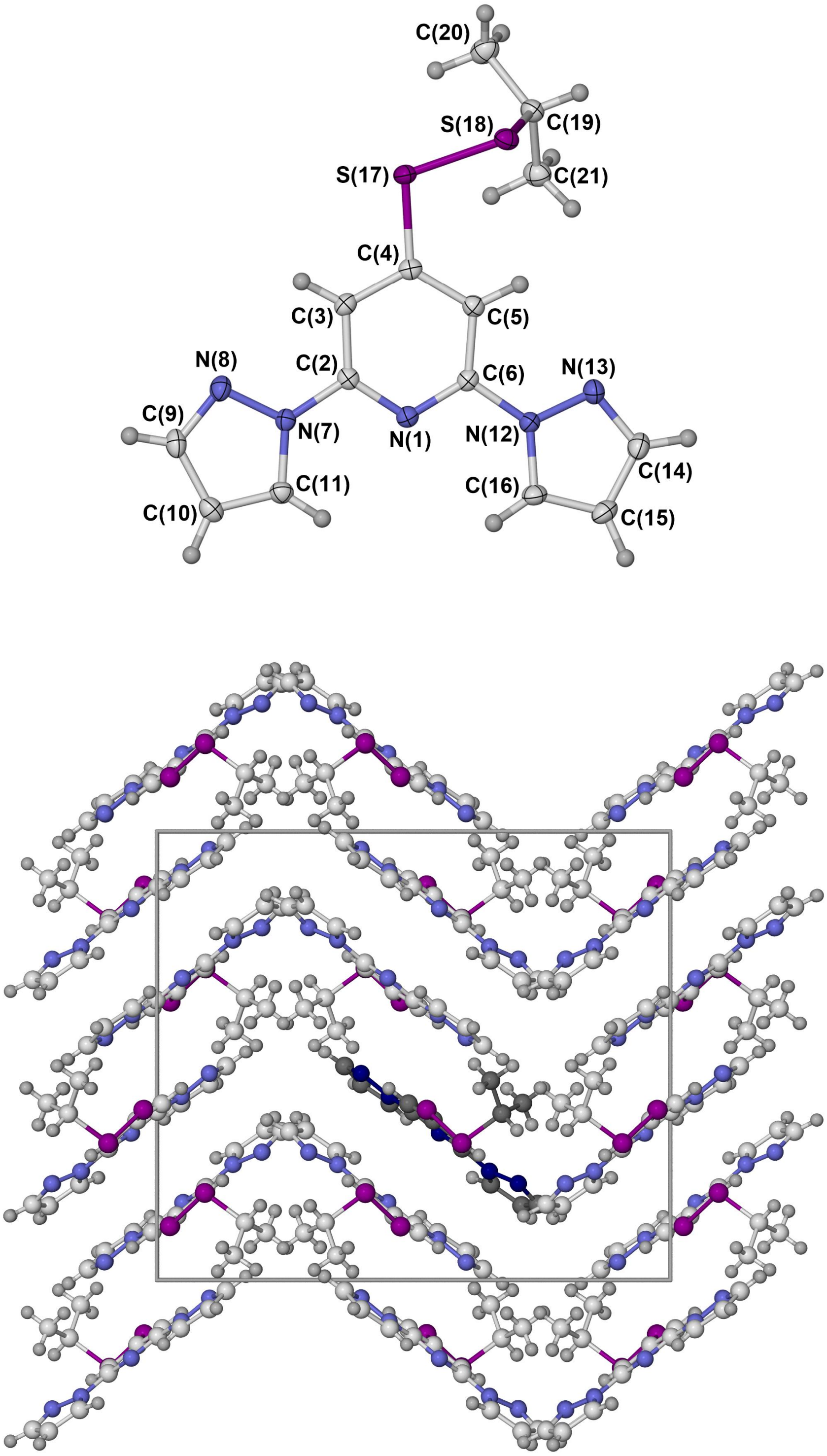


**Figure S2** Top: view of the symmetric unit in the crystal of $L^1$. Displacement ellipsoids are at the 50 % probability level, except H atoms which have arbitrary radii. Bottom: packing diagram of $L^1$ viewed perpendicular to the (100) plane. The unit cell is shown, and one molecule is highlighted with dark coloration.

Color code: C, white or dark gray; H, pale gray; N, pale or dark blue; S, purple.

Centrosymmetric pairs of molecules (related by $1-x$, $2-y$, $-z$) associate through a centrosymmetric face-to-face $\pi\cdots\pi$ contact, between the pyridyl and one pyrazolyl ring of each molecule. The interacting residues are coplanar by symmetry, and their least squares planes are separated by 3.326(2) Å.

## Definitions of the structural parameters in Tables S2, S6, S7, S11 and S14

$V_{Oh}$ is the volume (in Å$^3$) of the $FeN_6$ coordination octahedron in the complex,[7] which is typically <10 Å$^3$ in low-spin $[Fe(bpp)_2]^{2+}$ (bpp = 2,6-di{pyrazol-1-yl}pyridine) derivatives and ≥11.5 Å$^3$ in their high-spin form.[8]

$\Sigma$ and $\Theta$ are defined as follows:

$$\Sigma = \sum_{i=1}^{12} |90 - \beta_i| \qquad \Theta = \sum_{j=1}^{24} |60 - \gamma_j|$$

where $\beta_i$ are the twelve *cis*-N–Fe–N angles about the iron atom and $\gamma_i$ are the 24 unique N–Fe–N angles measured on the projection of two triangular faces of the octahedron along their common pseudo-threefold axis (Chart S1). $\Sigma$ is a general measure of the deviation of a metal ion from an ideal octahedral geometry, while $\Theta$ more specifically indicates its distortion towards a trigonal prismatic structure. A perfectly octahedral complex gives $\Sigma = \Theta = 0$.[7,9]

Because the high-spin state of a complex has a much more plastic structure than the low-spin, this is reflected in $\Sigma$ and $\Theta$ which are usually much larger in the high-spin state. The absolute values of these parameters depend on the metal/ligand combination in the compound under investigation, however. Typical values of these parameters for complexes related to $[Fe(bpp^R)_2]^{2+}$ are given in refs. 10 and 11.

**Chart S1** Angles used in the definitions of the coordination distortion parameters $\Sigma$ and $\Theta$.

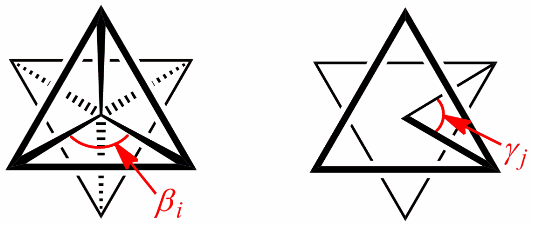


The parameters in Chart S2 define the magnitude of an angular Jahn-Teller distortion, that is often observed in high-spin $[Fe(bpp)_2]^{2+}$ derivatives like $[FeL_2]^{2+}$ ($\theta \leq 90°$, $\phi \leq 180$ °).[11,12] They are also a useful indicator of the molecular geometry, in defining the disposition of the two ligands around the metal ion. Spin-crossover can be inhibited if $\theta$ and $\phi$ deviate too strongly from their ideal values, because the associated rearrangement to a more regular low-spin coordination geometry ($\theta \approx 90°$, $\phi \approx 180°$) cannot be accommodated by a rigid solid lattice.[12-14] In less distorted examples, significant changes in $\theta$ and $\phi$ between the spin states can be associated with enhanced SCO cooperativity.[15-17]

**Chart S2** $\theta$ and $\phi$, used to discuss the structures of $[FeL_2]^{2+}$ (L = $L^1$ or $L^2$)

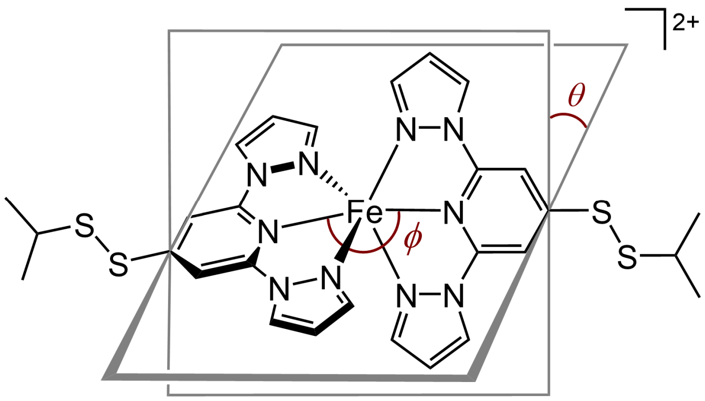

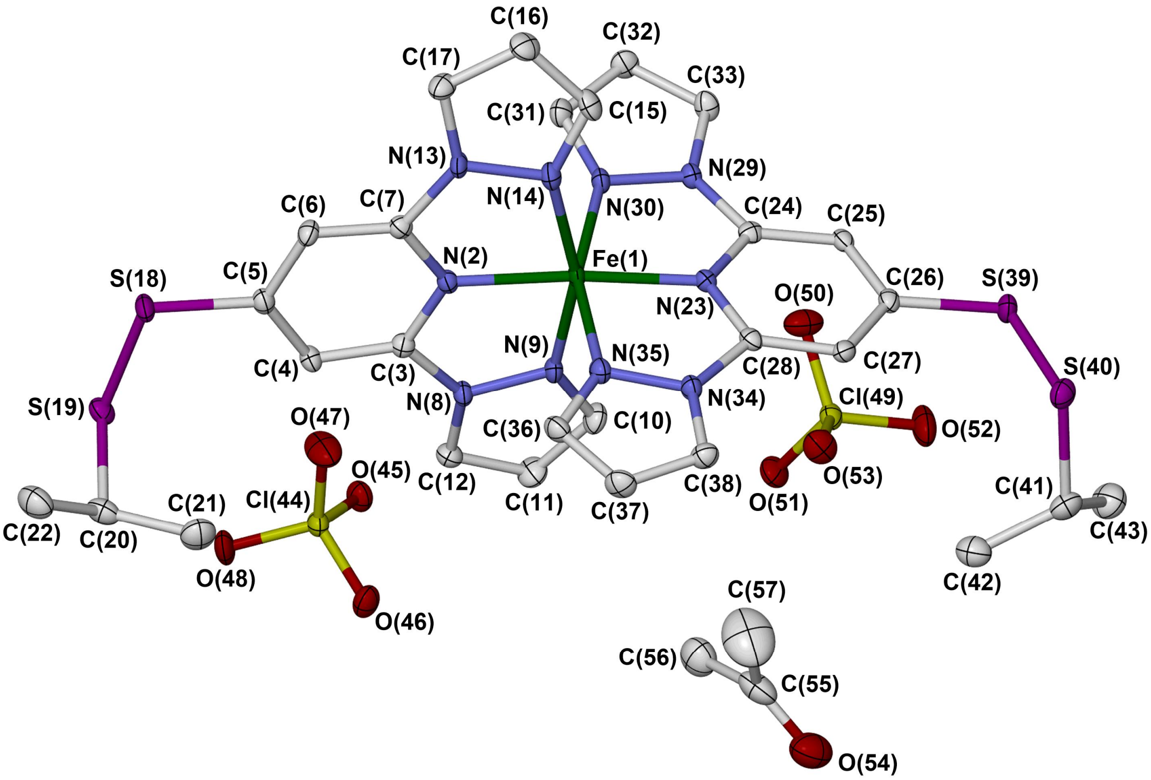


**Figure S3** The asymmetric unit of **1[ClO$_4$]$_2$**·Me$_2$CO at 100 K, with the full atom numbering scheme. Displacement ellipsoids are at the 50 % probability level, and H atoms are omitted for clarity. Color code: C, white; Cl, yellow; Fe, green; N, blue; O, red; S, purple.

The same atom numbering is used for all the **1[BF$_4$]$_2$** and **1[ClO$_4$]$_2$** structures in this study, with Cl(44)-O(53) replaced by B(44)-F(53) in the BF$_4^-$ salts. Cations in structures with two unique molecules per asymmetric unit follow the same atom numbering, and are distinguished with ‘A’ or ‘B’ suffixes.

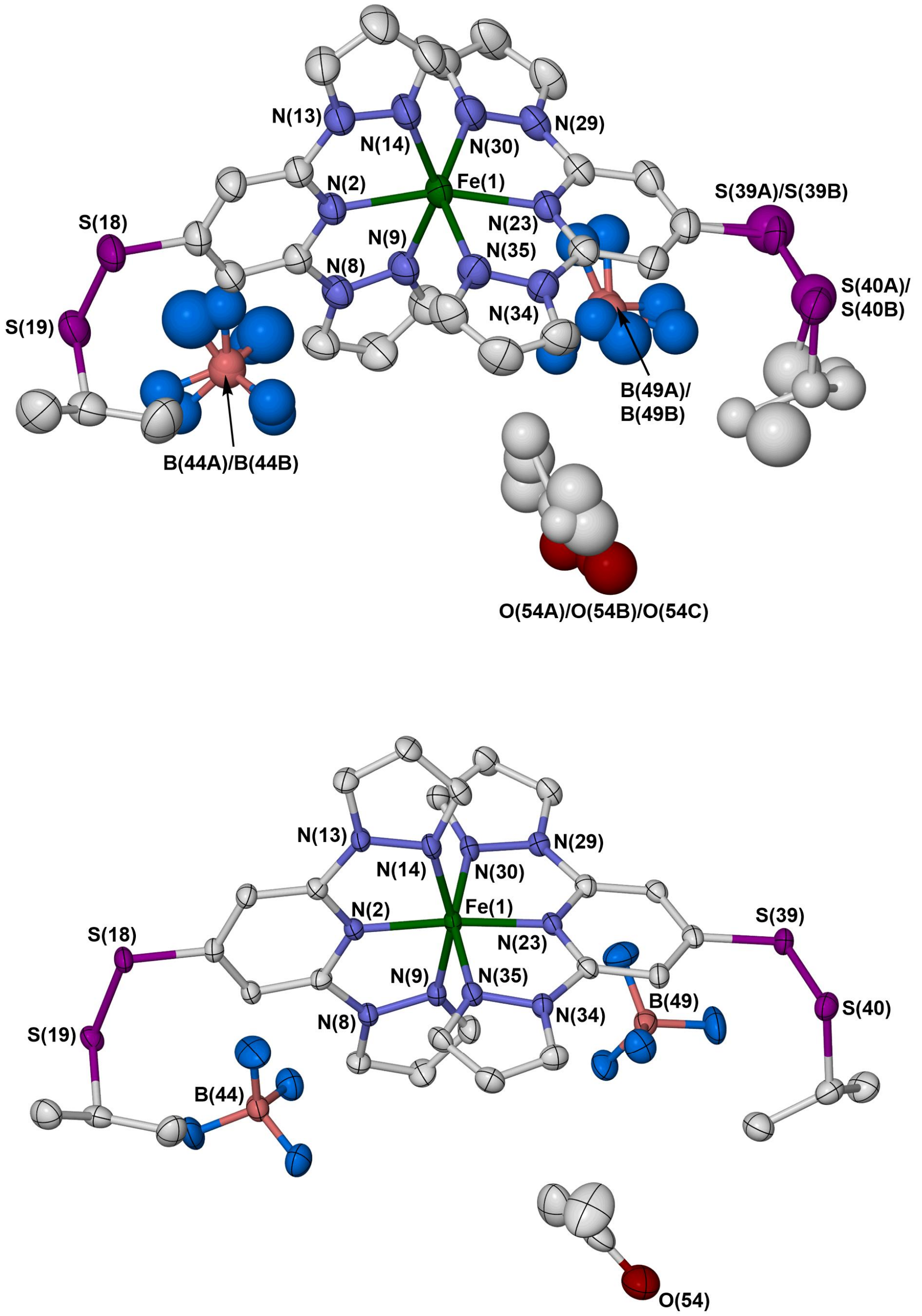


**Figure S4** The asymmetric unit of **1[BF$_4$]$_2$**·Me$_2$CO. Top, high-spin at 250 K; bottom, low-spin at 100 K. Other details as for Figure S3, which also shows the full atom numbering scheme for the structure. Color code: C, white; B, pink; F, cyan; Fe, green; N, blue; O, red; S, purple.

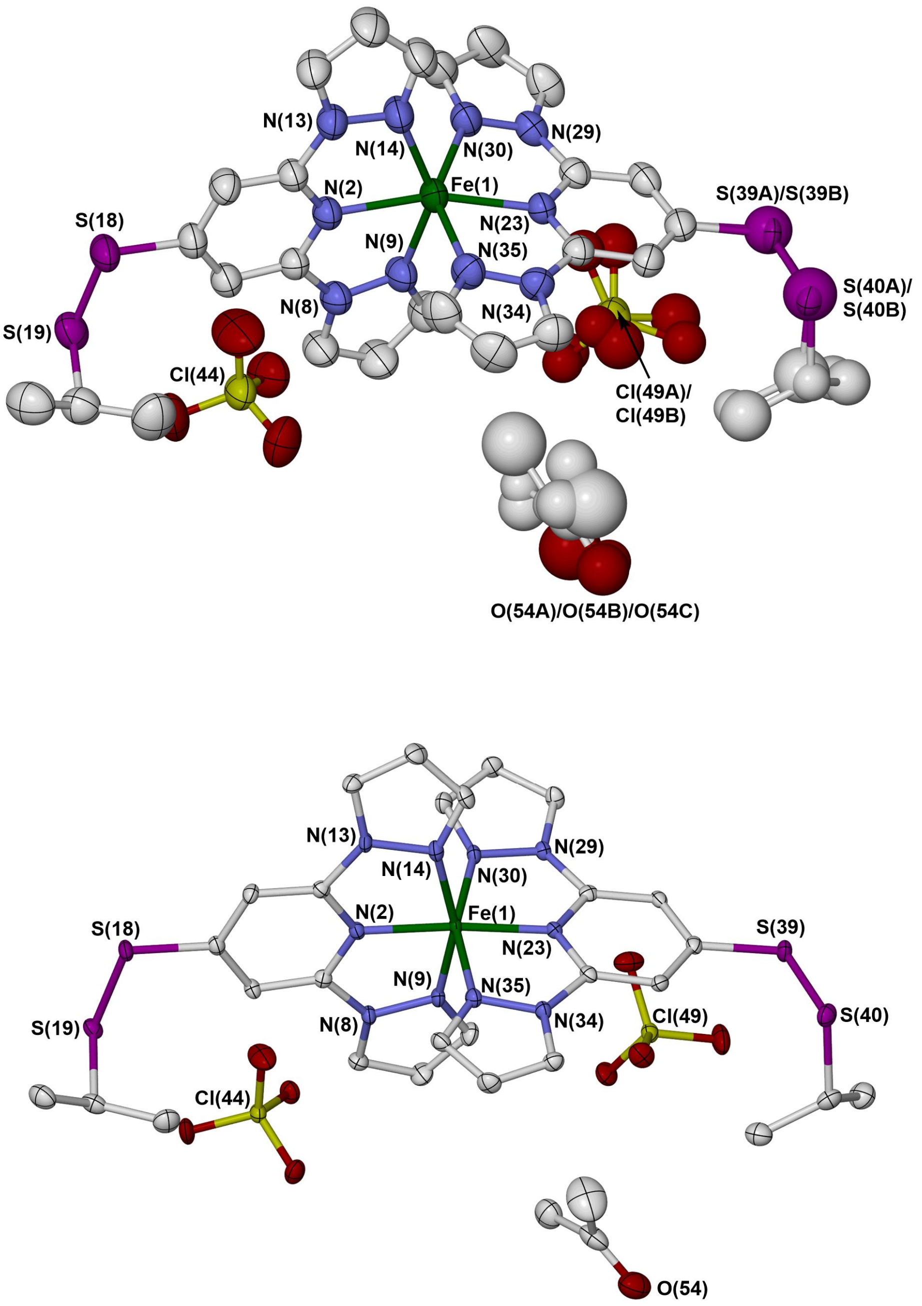


**Figure S5** The asymmetric unit of **1[ClO$_4$]$_2$**·Me$_2$CO. Top, high-spin at 250 K; bottom, low-spin at 100 K. Other details as for Figure S3, which also shows the full atom numbering scheme for the structure. Color code: C, white; Cl, yellow; Fe, green; N, blue; O, red; S, purple.

The low-temperature view is the same as in Figure S3.

**Table S2** Selected bond lengths, angles and other structural parameters (Å , °, Å$^3$) for **1X₂**·Me$_2$CO (X$^-$ = BF$_4^-$ or ClO$_4^-$). See Figures S3-S5 for the atom numbering scheme, while definitions of $V_{Oh}$, $\Sigma$, $\Theta$, $\varphi$ and $\theta$ are given on page S16.

| | **1[BF₄]₂**·Me₂CO | | **1[ClO₄]₂**·Me₂CO | |
|---|---|---|---|---|
| *T* / K | 143 | 250 | 100 | 250 |
| Fe(1)–N(2) | 1.903(2) | 2.127(3) | 1.903(2) | 2.132(3) |
| Fe(1)–N(9) | 1.979(2) | 2.193(4) | 1.976(2) | 2.196(3) |
| Fe(1)–N(14) | 1.997(3) | 2.175(4) | 1.997(2) | 2.181(3) |
| Fe(1)–N(23) | 1.906(2) | 2.131(3) | 1.907(2) | 2.131(3) |
| Fe(1)–N(30) | 1.967(2) | 2.163(4) | 1.974(2) | 2.166(3) |
| Fe(1)–N(35) | 1.974(3) | 2.197(4) | 1.979(2) | 2.195(3) |
| | | | | |
| N(2)–Fe(1)–N(9) | 79.62(9) | 72.75(12) | 79.84(8) | 72.95(11) |
| N(2)–Fe(1)–N(14) | 79.72(9) | 73.49(12) | 79.67(9) | 73.34(11) |
| N(2)–Fe(1)–N(23) | 174.33(10) | 163.01(13) | 175.18(9) | 163.71(12) |
| N(2)–Fe(1)–N(30) | 104.29(10) | 119.93(13) | 103.81(8) | 120.07(12) |
| N(2)–Fe(1)–N(35) | 96.14(10) | 94.50(13) | 96.83(9) | 94.54(12) |
| N(9)–Fe(1)–N(14) | 158.95(9) | 144.73(13) | 159.09(9) | 144.75(11) |
| N(9)–Fe(1)–N(23) | 96.46(9) | 96.31(12) | 96.84(9) | 97.09(11) |
| N(9)–Fe(1)–N(30) | 92.85(10) | 97.26(14) | 92.63(9) | 97.80(13) |
| N(9)–Fe(1)–N(35) | 90.92(10) | 92.38(14) | 91.27(9) | 92.63(13) |
| N(14)–Fe(1)–N(23) | 104.43(9) | 118.86(12) | 103.86(9) | 118.06(11) |
| N(14)–Fe(1)–N(30) | 88.38(10) | 90.82(14) | 88.27(9) | 90.25(13) |
| N(14)–Fe(1)–N(35) | 95.22(10) | 100.12(15) | 95.22(9) | 99.98(14) |
| N(23)–Fe(1)–N(30) | 79.88(10) | 73.55(13) | 79.73(8) | 73.34(11) |
| N(23)–Fe(1)–N(35) | 79.75(10) | 72.56(13) | 79.68(8) | 72.67(11) |
| N(30)–Fe(1)–N(35) | 159.57(10) | 145.56(13) | 159.36(9) | 145.39(12) |
| | | | | |
| $V_{Oh}$ | 9.591(7) | 12.010(13) | 9.622(7) | 12.061(12) |
| $\Sigma$ | 93.0(3) | 157.8(5) | 93.3(3) | 158.1(4) |
| $\Theta$ | 296 | 526 | 296 | 527 |
| $\phi$ | 174.33(10) | 163.01(13) | 175.18(9) | 163.71(12) |
| $\theta$ | 83.88(3) | 82.59(5) | 84.07(2) | 82.77(4) |

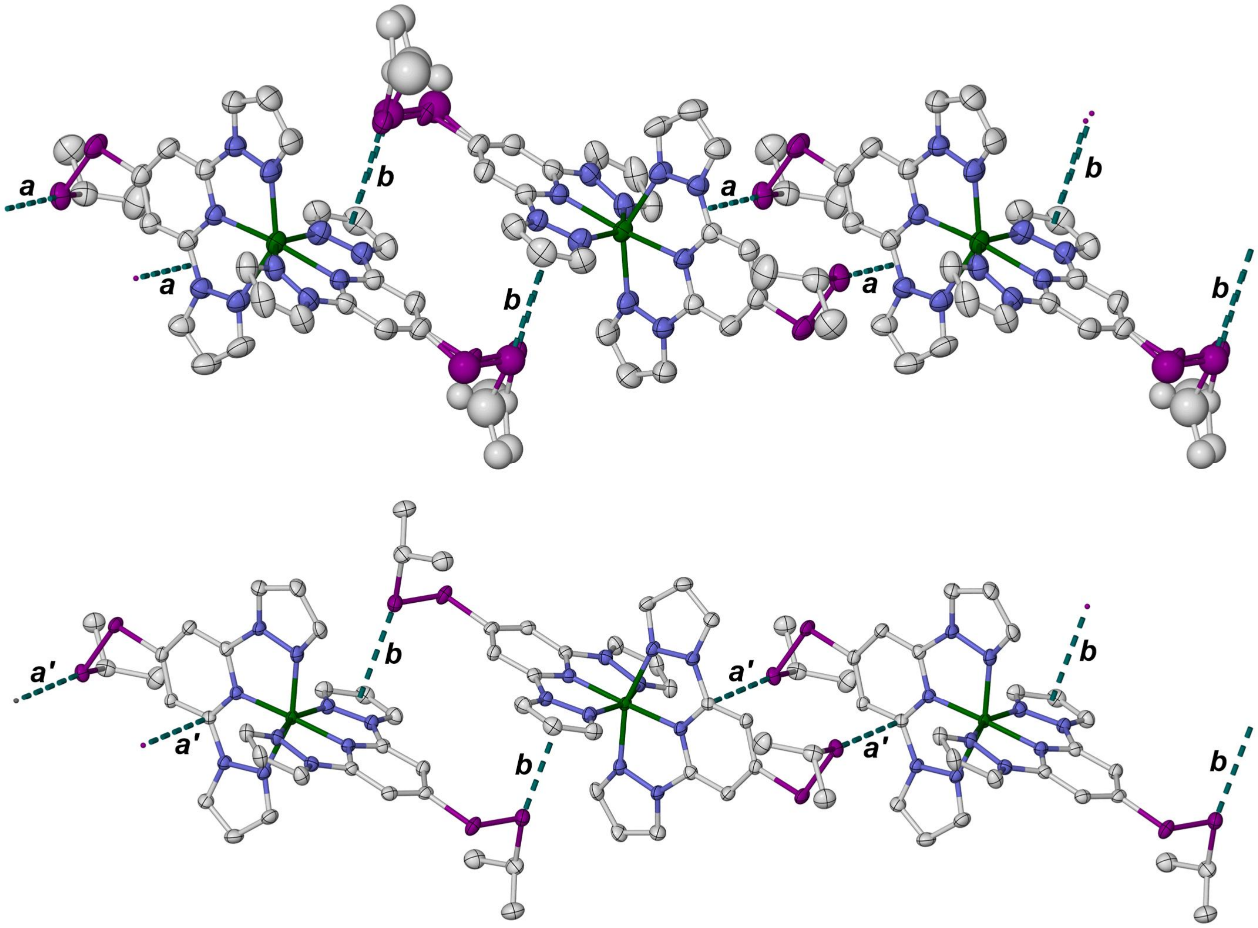


**Figure S6** Intermolecular $n\cdots\pi$ contacts in **1[BF$_4$]$_2$**·Me$_2$CO, showing the association of the cations into 1D chains: top, high-spin at 250 K; bottom, low-spin at 143 K. Color code: C, white; Fe, green; N, blue; S, purple.

The $\beta$-S atom from each *iso*propyldisulfanyl substituent is involved in one of these interactions. S(19) overlies the centroid of a C{pyridyl}−N{pyrazolyl} bond at 250 K, but is closer to the pyridyl C atom at 120 K. S(40) is closest to the centroid of a pyrazolyl ring in its nearest neighbor. The interactions are:

***a***: S(19)···[C(3$^{i}$)−N(8$^{i}$)] or ***a'***: S(19)···C(3$^{i}$) – symmetry code (i) 1−*x*, 1−*y*, 1−z.

***b***: S(40)···[C(3$^{ii}$)−N(8$^{ii}$)] – symmetry code (ii) −*x*, 1−*y*, −z.

The distances of these weak interactions are listed in the following Table.

**Table S3** Intermolecular $n\cdots\pi$ contacts for **1X$_2$**·Me$_2$CO (X$^-$ = BF$_4^-$ or ClO$_4^-$; Figure S6)

| Distance | ***a/a'*** | ***b*** |
|---|---|---|
| **1[BF$_4$]$_2$**·Me$_2$CO, 143 K | 3.266(3) (***a'***) | 3.396 |
| **1[BF$_4$]$_2$**·Me$_2$CO, 250 K | 3.447 (***a***) | 3.489/3.679[a] |
| | | |
| **1[ClO$_4$]$_2$**·Me$_2$CO, 100 K | 3.259(2) (***a'***) | 3.437 |
| **1[ClO$_4$]$_2$**·Me$_2$CO, 250 K | 3.423 (***a***) | 3.517/3.637[a] |

[a]The S atom of this *iso*propyldisulfanyl group is disordered at this temperature.

The sum of the Pauling van der Waals radii of an S atom and an aromatic ring is 3.55 Å.[18] Hence, contact ***a*** corresponds to a strongly attractive intermolecular interaction in all these structures, but ***b*** is longer and may just corresponds to a van der Waals contact in the 250 K refinements.

**Table S4** Variable temperature unit cell parameters for **1[BF$_4$]$_2$**·Me$_2$CO.

| *T* (K) | *a* (Å) | *b* (Å) | *c* (Å) | *β* (°) | *V* (Å$^3$) |
|---|---|---|---|---|---|
| Cooling | | | | | |
| 230 | 12.6972(8) | 17.0281(4) | 19.4613(6) | 105.101(4) | 4062.4(3) |
| 220 | 12.6929(9) | 17.0222(5) | 19.4786(7) | 105.118(5) | 4062.9(3) |
| 210 | 12.6682(9) | 17.0026(4) | 19.4673(7) | 105.122(5) | 4047.9(3) |
| 200 | 12.6392(8) | 16.9756(4) | 19.4563(6) | 105.161(4) | 4029.2(3) |
| 190 | 12.6191(8) | 16.9591(4) | 19.4534(6) | 105.181(4) | 4017.9(3) |
| 180 | 12.6006(9) | 16.9503(4) | 19.4556(7) | 105.205(5) | 4010.0(3) |
| 170 | 12.5747(8) | 16.9316(4) | 19.4488(6) | 105.244(4) | 3995.2(3) |
| 160 | 12.2924(11) | 16.8870(5) | 19.7058(9) | 104.558(6) | 3959.3(4) |
| 150 | 12.2405(9) | 16.8699(4) | 19.6871(8) | 104.533(5) | 3935.2(4) |
| 140 | 12.2104(9) | 16.8659(4) | 19.6900(7) | 104.555(5) | 3924.8(3) |
| 130 | 12.1912(10) | 16.8672(5) | 19.6939(8) | 104.559(5) | 3919.6(4) |
| 120 | 12.1695(13) | 16.8632(6) | 19.6995(10) | 104.561(7) | 3912.8(5) |
| | | | | | |
| Warming | | | | | |
| 120 | 12.1682(11) | 16.8662(5) | 19.7063(9) | 104.563(6) | 3914.4(4) |
| 130 | 12.1918(10) | 16.8773(5) | 19.7103(8) | 104.545(5) | 3925.7(4) |
| 140 | 12.2132(10) | 16.8741(5) | 19.7020(8) | 104.534(5) | 3930.4(4) |
| 150 | 12.2416(10) | 16.8735(5) | 19.6988(8) | 104.524(5) | 3938.9(4) |
| 160 | 12.2708(10) | 16.8681(5) | 19.6873(8) | 104.503(5) | 3945.1(4) |
| 170 | 12.5639(8) | 16.9267(4) | 19.4553(6) | 105.249(4) | 3991.8(3) |
| 180 | 12.5989(9) | 16.9554(4) | 19.4636(7) | 105.204(5) | 4012.3(3) |
| 190 | 12.6129(9) | 16.9557(4) | 19.4532(7) | 105.190(5) | 4014.9(3) |
| 200 | 12.6364(9) | 16.9774(4) | 19.4591(7) | 105.157(5) | 4029.4(3) |
| 210 | 12.6571(9) | 16.9920(4) | 19.4630(7) | 105.143(5) | 4040.5(3) |
| 220 | 12.6783(8) | 17.0107(4) | 19.4656(7) | 105.123(5) | 4052.7(3) |

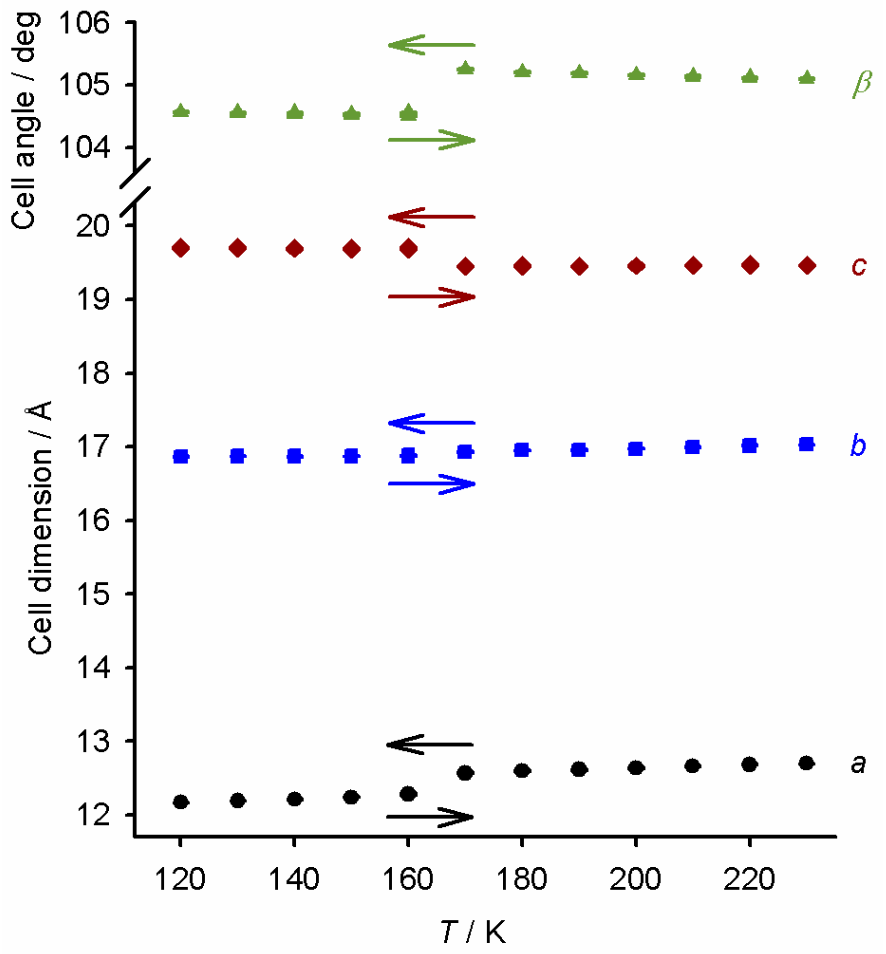


**Figure S7** Variable temperature unit cell dimensions for **1[BF$_4$]$_2$**·Me$_2$CO (Table S4). Data were collected in cooling and warming temperature ramps. No thermal hysteresis in the spin-transition is evident in these data. Error bars are shown, but are mostly smaller than the symbols on the graph.

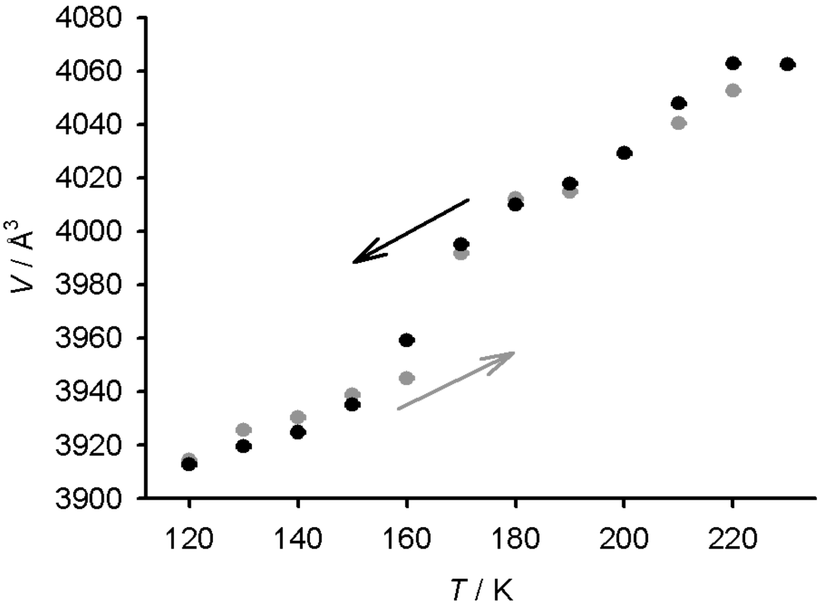


**Figure S8** Variable temperature unit cell volumes for **1[BF$_4$]$_2$**·Me$_2$CO, measured with cooling (black) and warming (gray) temperature ramps. Error bars are mostly smaller than the symbols on the graph.

**Table S5** Variable temperature unit cell parameters for **1[ClO$_4$]$_2$**·Me$_2$CO.

| *T* (K) | *a* (Å) | *b* (Å) | *c* (Å) | *β* (°) | *V* (Å$^3$) |
|---|---|---|---|---|---|
| Cooling | | | | | |
| 250 | 12.4965(6) | 17.2660(5) | 19.8580(13) | 105.488(6) | 4129.1(4) |
| 240 | 12.4506(5) | 17.2574(5) | 19.8830(12) | 105.495(5) | 4116.9(3) |
| 230 | 12.4039(5) | 17.2444(5) | 19.8979(12) | 105.526(5) | 4100.8(3) |
| 220 | 12.3542(5) | 17.2352(5) | 19.9211(11) | 105.553(5) | 4086.4(3) |
| 210 | 12.3142(4) | 17.2375(4) | 19.9511(10) | 105.563(5) | 4079.7(3) |
| 200 | 12.2731(5) | 17.2332(4) | 19.9723(11) | 105.568(5) | 4069.2(3) |
| 190 | 12.2376(4) | 17.2227(4) | 19.9722(10) | 105.571(5) | 4055.0(3) |
| 180 | 12.2168(4) | 17.2222(4) | 19.9891(9) | 105.592(5) | 4050.9(3) |
| 170 | 12.1897(4) | 17.2140(4) | 19.9869(9) | 105.591(4) | 4039.6(2) |
| 160 | 12.1719(5) | 17.2119(4) | 19.9931(11) | 105.590(5) | 4034.5(3) |
| 150 | 12.034(2) | 17.1124(18) | 20.027(4) | 104.33(2) | 3996(1) |
| 140 | 12.0158(4) | 17.0400(3) | 20.0009(9) | 104.077(4) | 3972.2(2) |
| 130 | 12.0109(4) | 17.0410(3) | 20.0046(9) | 104.076(4) | 3971.5(2) |
| 120 | 11.9943(4) | 17.0239(3) | 19.9910(9) | 104.070(4) | 3959.5(2) |
| | | | | | |
| Warming | | | | | |
| 130 | 12.0101(4) | 17.0406(3) | 20.0071(8) | 104.080(4) | 3971.6(2) |
| 140 | 12.0160(4) | 17.0405(4) | 20.0013(9) | 104.079(5) | 3972.4(2) |
| 150 | 12.0326(4) | 17.0552(3) | 20.0104(8) | 104.081(4) | 3983.1(2) |
| 160 | 12.0408(4) | 17.0559(3) | 20.0077(9) | 104.084(4) | 3985.4(2) |
| 170 | 12.1848(5) | 17.2110(4) | 19.9978(11) | 105.565(5) | 4040.0(3) |
| 180 | 12.2142(5) | 17.2204(4) | 19.9964(10) | 105.569(5) | 4051.6(3) |
| 190 | 12.2416(5) | 17.2214(4) | 19.9794(10) | 105.557(5) | 4057.7(3) |
| 200 | 12.2776(5) | 17.2260(5) | 19.9682(11) | 105.559(5) | 4068.4(3) |
| 210 | 12.3209(5) | 17.2336(4) | 19.9472(11) | 105.562(5) | 4080.2(3) |
| 220 | 12.3597(5) | 17.2303(5) | 19.9158(12) | 105.540(5) | 4086.3(3) |
| 230 | 12.4075(5) | 17.2381(4) | 19.8997(11) | 105.517(5) | 4101.1(3) |
| 240 | 12.4529(6) | 17.2494(5) | 19.8800(12) | 105.500(6) | 4115.0(3) |
| 250 | 12.4969(5) | 17.2642(5) | 19.8632(11) | 105.492(5) | 4129.8(3) |

SCO in **1[BF$_4$]$_2$**·Me$_2$CO leads to significant changes in *a* and *c*, as well as *β* (Table S4, Figure S7). That is not evident in this salt, however, where the spin transition is only clearly evident in the unit cell angle *β*.

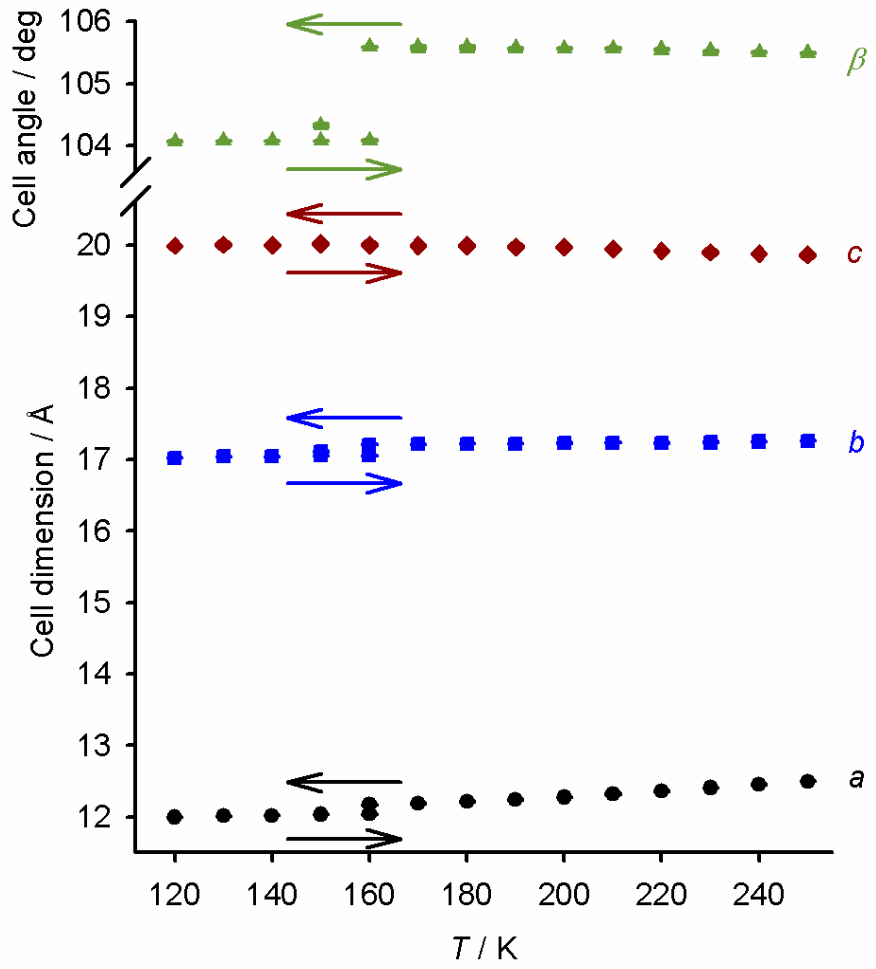


**Figure S9** Variable temperature unit cell dimensions for **1[ClO$_4$]$_2$**·Me$_2$CO (Table S5). Data were collected in cooling and warming temperature ramps. Thermal hysteresis in the spin-transition is visible in *a*, *b* and *β*. Error bars are shown, but are mostly smaller than the symbols on the graph.

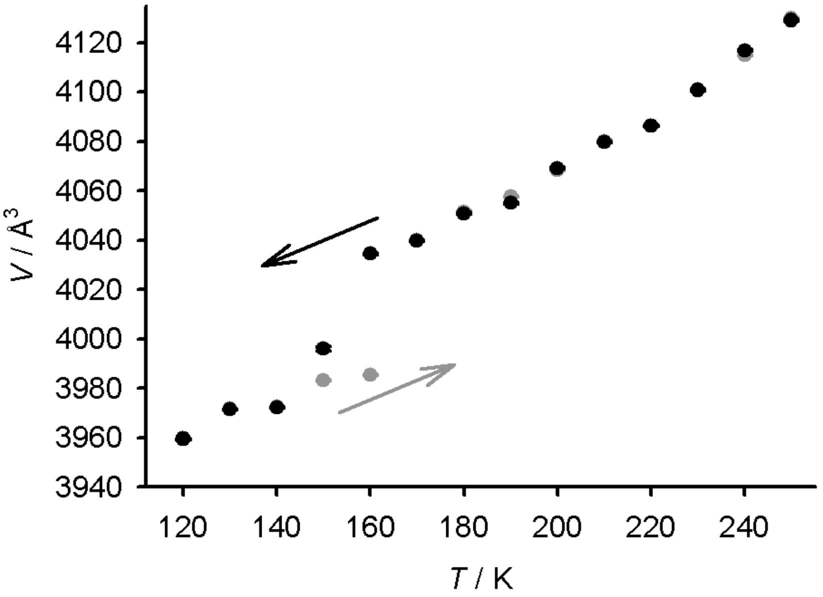


**Figure S10** Variable temperature unit cell volumes for **1[ClO$_4$]$_2$**·Me$_2$CO, measured with cooling (black) and warming (gray) temperature ramps. Error bars are mostly smaller than the symbols on the graph.

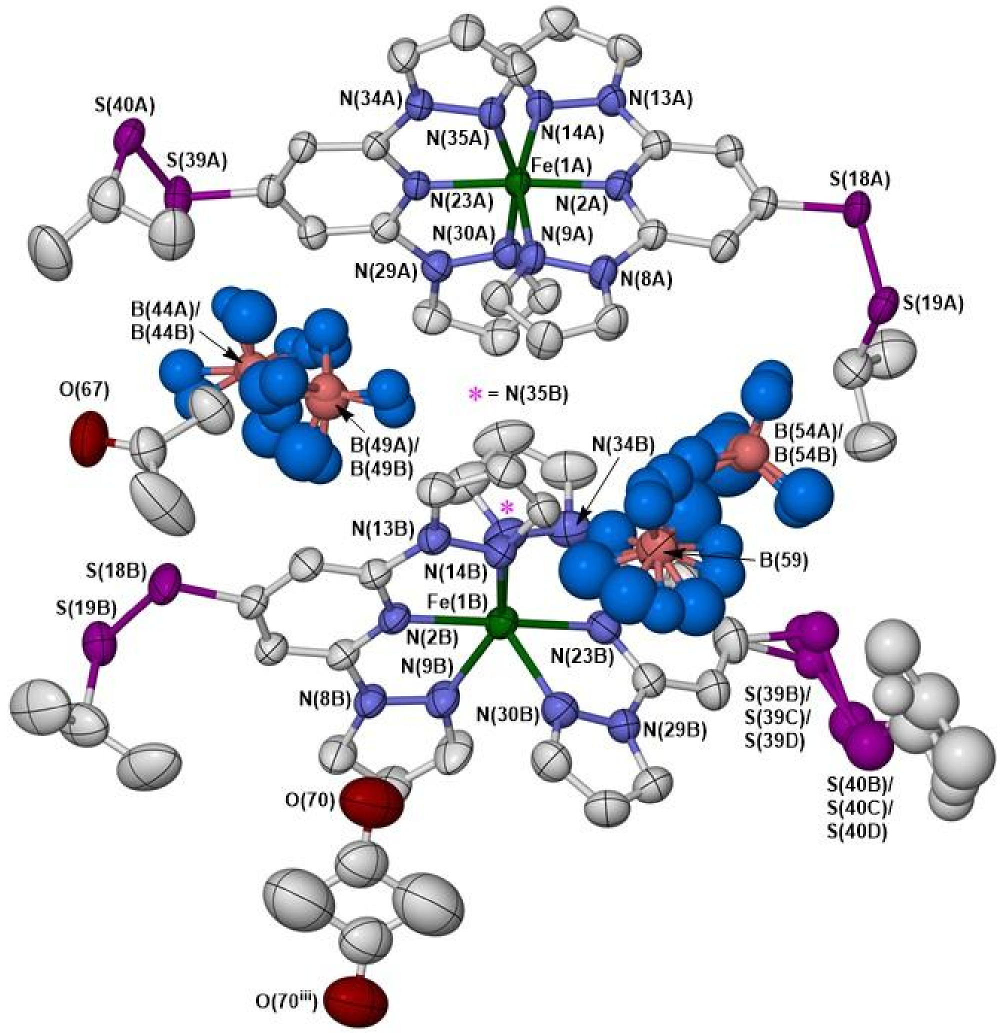


**Figure S11** The asymmetric unit of **1[BF$_4$]$_2$**·0.75Me$_2$CO. This page, at 250 K; next page, at 120 K. The full atom numbering in the structure corresponds to that in Figure S3, with suffixes to distinguish between cations A and B in the model. Other details as for Figure S3. Color code: C, white; B, pink; F, cyan; Fe, green; N, blue; O, red; S, purple.

Symmetry code: (iii) 1−*x*, 2−*y*, −*z*.

Acetone half-molecule C(68)-O(70) spans a crystallographic inversion center. See the following page for additional discussion of this crystal.

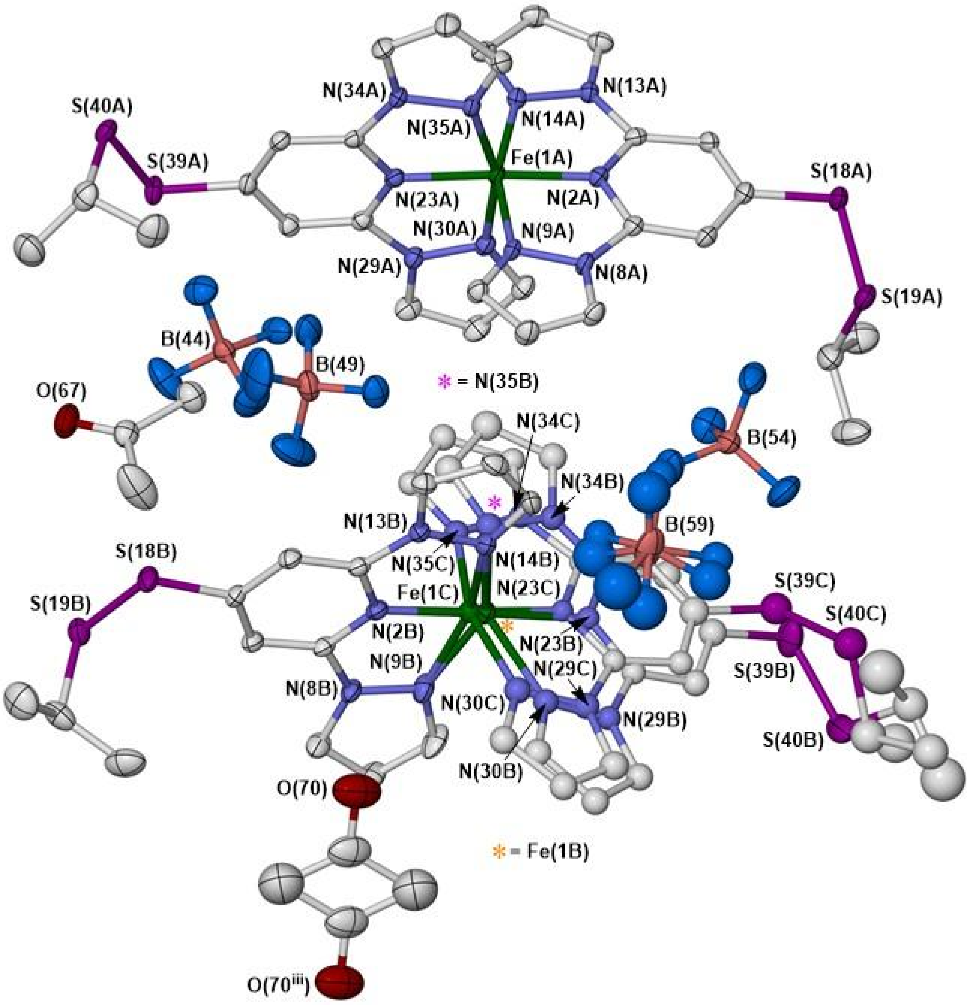


**Figure S11** continued.

Symmetry code: (iii) 1−*x*, 2−*y*, −*z*.

Atoms Fe(1B), N(23B)-C(43B) and Fe(1C), N(23C)-C(43C) in the low temperature refinement correspond to the high-spin and low-spin populations of that lattice site, respectively. The 'B' and 'C' disorder sites had a refined occupancy ratio of 0.74 (high-spin):0.26 (low-spin). The disordered heterocycle core was modelled without restraints, but fixed S–S, S–C, C–C and 1,3-C···C distance restraints were applied to its peripheral *iso*propyldisulfanyl substituents.

The heterocyclic core of molecule B is not disordered in the 250 K structure of the same crystal, where that molecule is fully high-spin (previous page).

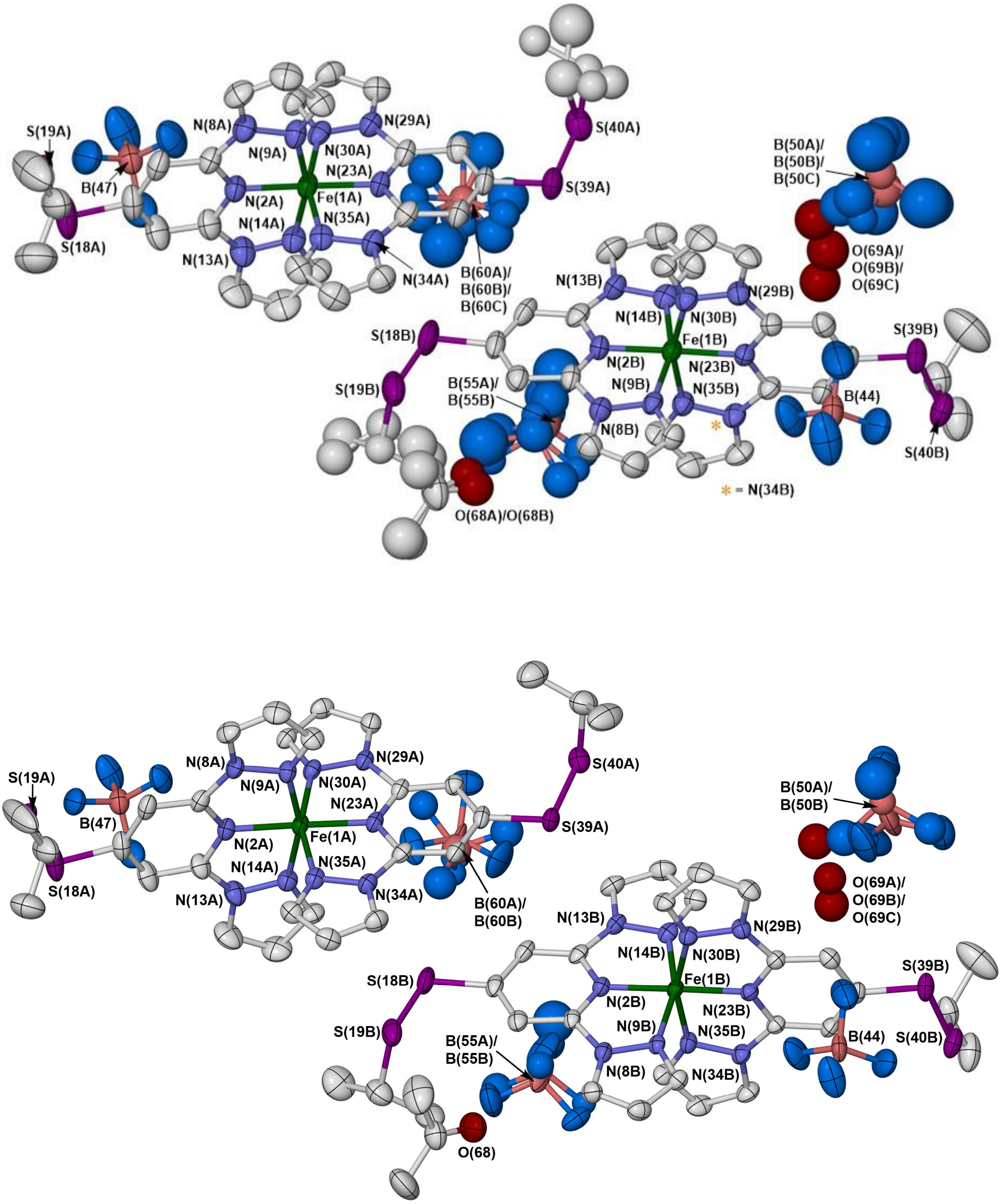


**Figure S12** The asymmetric unit of **1[BF$_4$]$_2$**·0.5Me$_2$CO·0.5H$_2$O. Top, at 250 K; bottom, at 120 K (the crystal is low-spin at both temperatures). The full atom numbering in the structure corresponds to that in Figure S3, with suffixes to distinguish between cations A and B in the model. Other details as for Figure S3. Color code: C, white; B, pink; F, cyan; Fe, green; N, blue; O, red; S, purple.

Atoms B(44) and B(47) lie on crystallographic $C_2$ axes, so those anions are only half occupied by symmetry. The other three disordered anions are fully occupied, on general crystallographic sites.

The acetone molecule C(65)-O(68) is fully occupied, while O(69A)-O(69C) are three partial water sites whose occupancies sum to 1. Those water sites lie within hydrogen bonding distance of the disordered anion B(50)-F(54), and its symmetry equivalent related by *x*, 1+*y*, *z*.

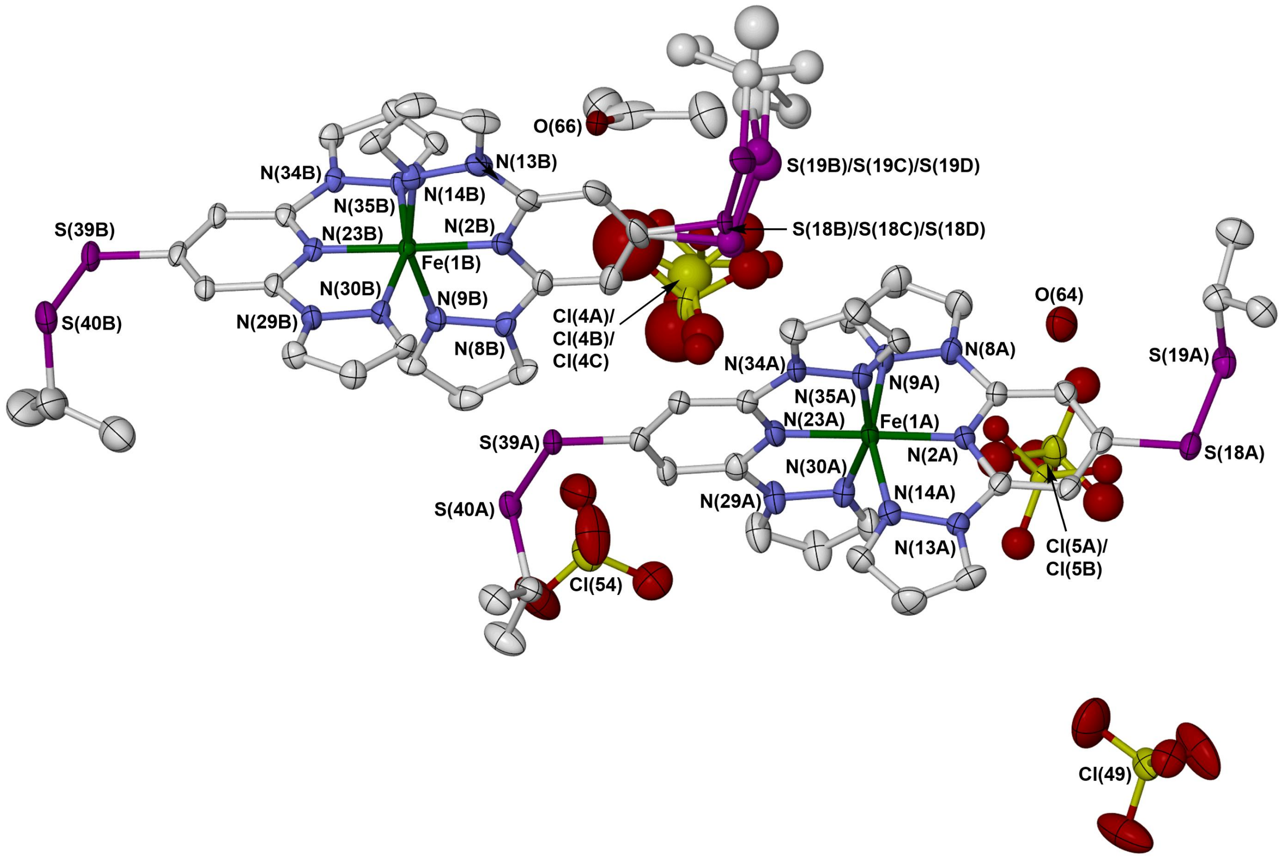


**Figure S13** The asymmetric unit of **1[ClO$_4$]$_2$**·*m*Me$_2$CO·0.5H$_2$O ($m \approx 0.34$) at 120 K. The full atom numbering in the structure corresponds to that in Figure S3, with suffixes to distinguish between cations A and B in the model. Other details as for Figure S3. Color code: C, white; Cl, yellow; Fe, green; N, blue; O, red; S, purple.

Acetone molecule C(65)-C(68) has an occupancy of 0.67 in the model, while water molecule O(68) is fully occupied. The H atoms bound to O(68) were not located in the Fourier map, but this water molecule is positioned to hydrogen bond to the perchlorate ion O atoms O(52$^{iii}$) [O(68)···O(56$^{iv}$) = 2.89 Å] and O(56$^{v}$) [O(68)···O(56$^{iv}$) = 2.87 Å]. Symmetry codes: (iv) *x*, ½−*y*, ½+*z*; (v) 1−*x*, −½+*y*, ½−*z*.

**Table S2** Selected bond lengths, angles and other structural parameters (Å , °, $Å^3$) for the other acetone solvates of $\mathbf{1X_2}$ ($X^-$ = $BF_4^-$ or $ClO_4^-$). See Figures S11-S13 for the atom numbering scheme, while definitions of $V_{Oh}$, $\Sigma$, $\Theta$, $\varphi$ and $\theta$ are given on page S16.

| | $\mathbf{1[BF_4]_2}\cdot 0.75Me_2CO$ | | | |
|---|---|---|---|---|
| *T* / K | 120 | | 250 | |
| | Molecule A | Molecule B[a] | Molecule A | Molecule B |
| Fe(1)–N(2) | 1.894(2) | 2.147(3)/1.859(4) | 1.891(3) | 2.116(3) |
| Fe(1)–N(9) | 1.962(2) | 2.160(3)/2.050(5) | 1.968(3) | 2.166(4) |
| Fe(1)–N(14) | 1.968(2) | 2.142(3)/2.126(5) | 1.978(3) | 2.180(3) |
| Fe(1)–N(23) | 1.893(2) | 2.135(4)/1.893(12) | 1.893(3) | 2.123(3) |
| Fe(1)–N(30) | 1.964(2) | 2.188(4)/1.964(12) | 1.967(3) | 2.181(4) |
| Fe(1)–N(35) | 1.968(2) | 2.160(4)/1.958(10) | 1.971(3) | 2.164(4) |
| | | | | |
| N(2)–Fe(1)–N(9) | 80.46(9) | 72.41(9)/81.05(19) | 80.30(13) | 73.29(13) |
| N(2)–Fe(1)–N(14) | 80.05(9) | 73.15(9)/79.46(18) | 79.91(12) | 73.34(12) |
| N(2)–Fe(1)–N(23) | 175.76(9) | 172.50(12)/177.0(4) | 175.47(13) | 172.71(13) |
| N(2)–Fe(1)–N(30) | 95.38(9) | 110.84(15)/103.9(4) | 95.12(13) | 111.08(13) |
| N(2)–Fe(1)–N(35) | 104.24(9) | 102.90(15)/96.7(4) | 104.56(13) | 102.60(14) |
| N(9)–Fe(1)–N(14) | 160.45(9) | 145.13(11)/158.8(2) | 160.15(13) | 146.04(13) |
| N(9)–Fe(1)–N(23) | 99.13(9) | 101.47(11)/98.2(3) | 99.15(12) | 101.13(13) |
| N(9)–Fe(1)–N(30) | 92.15(9) | 89.94(12)/87.0(3) | 91.81(13) | 89.58(15) |
| N(9)–Fe(1)–N(35) | 90.57(9) | 99.24(12)/96.2(3) | 90.77(13) | 98.69(15) |
| N(14)–Fe(1)–N(23) | 100.42(9) | 113.28(12)/101.7(3) | 100.70(12) | 112.63(13) |
| N(14)–Fe(1)–N(30) | 91.08(9) | 97.23(12)/89.6(3) | 91.51(13) | 96.58(14) |
| N(14)–Fe(1)–N(35) | 92.82(9) | 93.52(12)/94.4(3) | 92.66(13) | 94.48(14) |
| N(23)–Fe(1)–N(30) | 80.41(9) | 72.92(16)/78.9(4) | 80.39(13) | 73.06(14) |
| N(23)–Fe(1)–N(35) | 79.97(9) | 73.43(16)/80.5(5) | 79.93(12) | 73.28(15) |
| N(30)–Fe(1)–N(35) | 160.38(9) | 146.24(19)/159.4(5) | 160.31(13) | 146.28(15) |
| | | | | |
| $V_{Oh}$ | 9.455(7) | 12.115(12)/9.89(3) | 9.491(9) | 12.148(13) |
| $\Sigma$ | 84.9(3) | 156.6(4)/94.6(12) | 85.6(4) | 154.6(4) |
| $\Theta$ | 280 | 514/290 | 283 | 507 |
| $\phi$ | 175.76(9) | 172.50(12)/177.0(4) | 175.47(13) | 172.71(13) |
| $\theta$ | 88.19(2) | 84.18(3)/86.25(8) | 88.72(4) | 84.59(5) |

[a]Iron atom Fe(1B) and one of its *L* ligands are disordered at this temperature (Figure S11). While the Fe–N distances are ambiguous, the bond angles and structural parameters show this disorder corresponds to separately resolved high-spin (74 % occupancy) and low-spin (26 %) forms of this cation site.

**Table S2** continued.

| | **1[BF$_4$]$_2$**·0.5Me$_2$CO·0.5H$_2$O | | | | **1[ClO$_4$]$_2$**·$m$Me$_2$CO·0.5H$_2$O ($m \approx 0.34$) | |
|---|---|---|---|---|---|---|
| $T$ / K | 120 | | 250 | | 120 | |
| | Molecule A | Molecule B | Molecule A | Molecule B | Molecule A | Molecule B |
| Fe(1)–N(2) | 1.893(4) | 1.893(4) | 1.887(5) | 1.897(5) | 1.894(5) | 1.897(4) |
| Fe(1)–N(9) | 1.962(4) | 1.963(4) | 1.962(6) | 1.967(5) | 1.978(5) | 1.986(5) |
| Fe(1)–N(14) | 1.975(4) | 1.972(4) | 1.976(6) | 1.969(6) | 1.970(5) | 1.981(5) |
| Fe(1)–N(23) | 1.896(4) | 1.894(4) | 1.892(5) | 1.898(5) | 1.896(5) | 1.896(4) |
| Fe(1)–N(30) | 1.971(4) | 1.974(4) | 1.967(6) | 1.980(5) | 1.968(5) | 1.983(5) |
| Fe(1)–N(35) | 1.953(4) | 1.966(4) | 1.947(6) | 1.968(5) | 1.981(5) | 1.968(5) |
| | | | | | | |
| N(2)–Fe(1)–N(9) | 80.25(17) | 80.01(16) | 80.0(2) | 79.6(2) | 79.7(2) | 79.98(19) |
| N(2)–Fe(1)–N(14) | 80.27(17) | 80.56(17) | 80.3(2) | 80.8(2) | 80.1(2) | 80.0(2) |
| N(2)–Fe(1)–N(23) | 176.49(18) | 178.20(17) | 176.6(2) | 177.9(2) | 178.3(2) | 178.1(2) |
| N(2)–Fe(1)–N(30) | 103.49(17) | 101.49(16) | 103.3(2) | 102.1(2) | 100.1(2) | 102.10(19) |
| N(2)–Fe(1)–N(35) | 96.24(17) | 98.06(16) | 96.5(2) | 97.6(2) | 99.7(2) | 98.08(19) |
| N(9)–Fe(1)–N(14) | 160.50(17) | 160.56(16) | 160.3(2) | 160.3(2) | 159.7(2) | 159.92(19) |
| N(9)–Fe(1)–N(23) | 98.34(17) | 100.54(16) | 98.6(2) | 100.8(2) | 102.0(2) | 100.18(18) |
| N(9)–Fe(1)–N(30) | 92.16(17) | 93.01(16) | 92.1(2) | 92.9(2) | 89.6(2) | 94.59(18) |
| N(9)–Fe(1)–N(35) | 90.58(17) | 90.48(16) | 90.8(2) | 90.3(2) | 94.4(2) | 89.84(19) |
| N(14)–Fe(1)–N(23) | 101.06(17) | 98.88(16) | 101.0(2) | 98.9(2) | 98.3(2) | 99.90(19) |
| N(14)–Fe(1)–N(30) | 93.17(17) | 91.00(16) | 93.1(2) | 91.0(2) | 92.6(2) | 88.55(19) |
| N(14)–Fe(1)–N(35) | 90.69(17) | 92.07(16) | 90.7(2) | 92.4(2) | 90.4(2) | 94.02(19) |
| N(23)–Fe(1)–N(30) | 79.73(16) | 80.21(16) | 79.8(2) | 79.9(2) | 80.5(2) | 79.83(18) |
| N(23)–Fe(1)–N(35) | 80.53(17) | 80.24(16) | 80.3(2) | 80.3(2) | 79.7(2) | 79.99(18) |
| N(30)–Fe(1)–N(35) | 160.26(16) | 160.44(16) | 160.1(2) | 160.2(2) | 160.2(2) | 159.79(18) |
| | | | | | | |
| $V_{Oh}$ | 9.463(12) | 9.502(12) | 9.417(16) | 9.538(16) | 9.549(15) | 9.591(14) |
| $\Sigma$ | 85.0(6) | 84.5(6) | 85.7(7) | 85.4(7) | 87.9(7) | 90.7(7) |
| $\Theta$ | 280 | 278 | 282 | 282 | 286 | 288 |
| $\phi$ | 176.49(18) | 178.20(17) | 176.6(2) | 177.9(2) | 178.3(2) | 178.1(2) |
| $\theta$ | 89.98(4) | 87.96(4) | 89.78(6) | 88.14(5) | 84.97(5) | 83.33(4) |

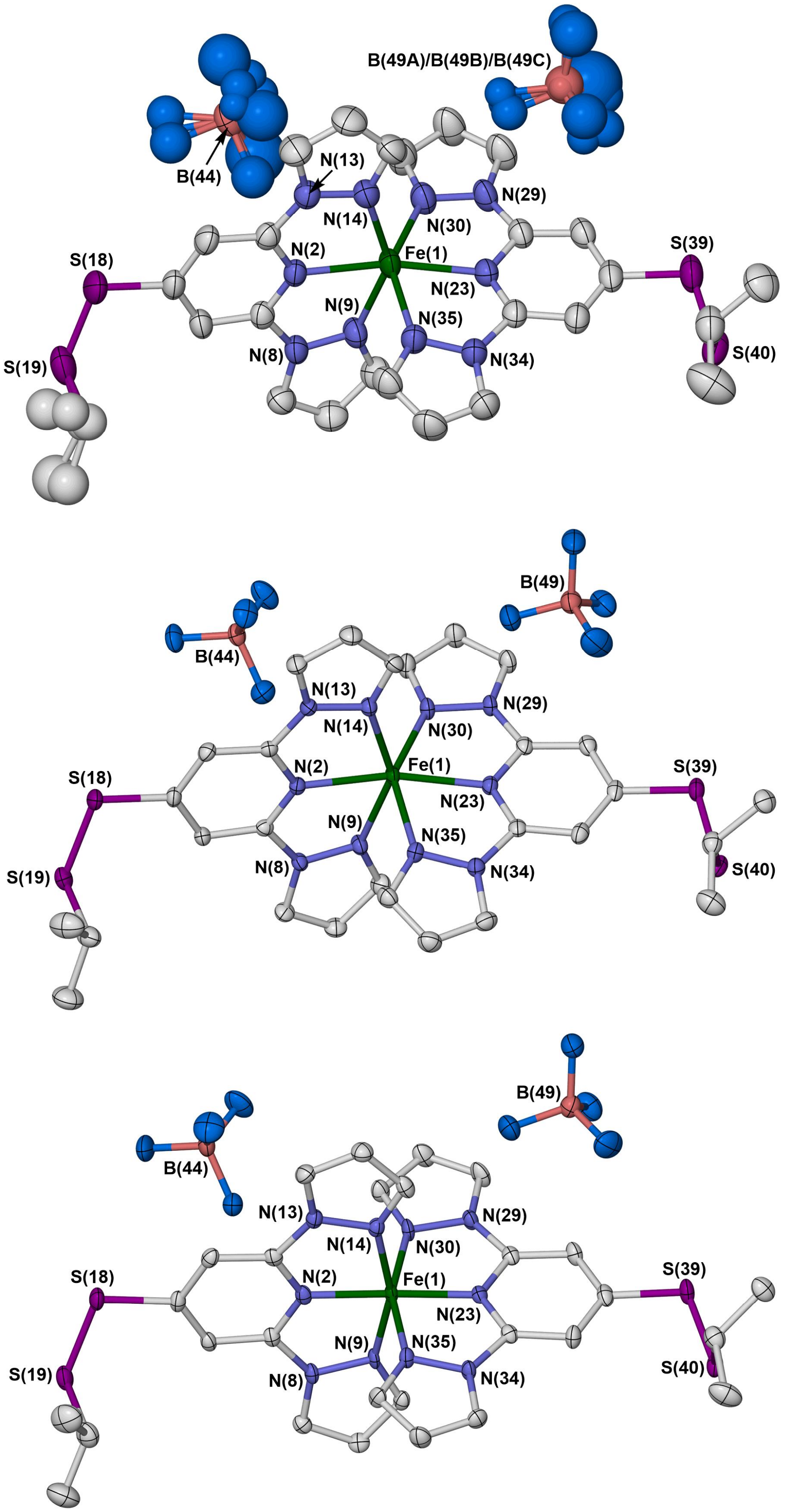


**Figure S14** The asymmetric unit of **1[BF$_4$]$_2$**·sf. Top, high-spin at 250 K; center, high-spin at 100 K; bottom, low-spin at 100 K. Other details as for Figure S3, which also shows the full atom numbering scheme for the structure. Color code: C, white; B, pink; F, cyan; Fe, green; N, blue; S, purple.

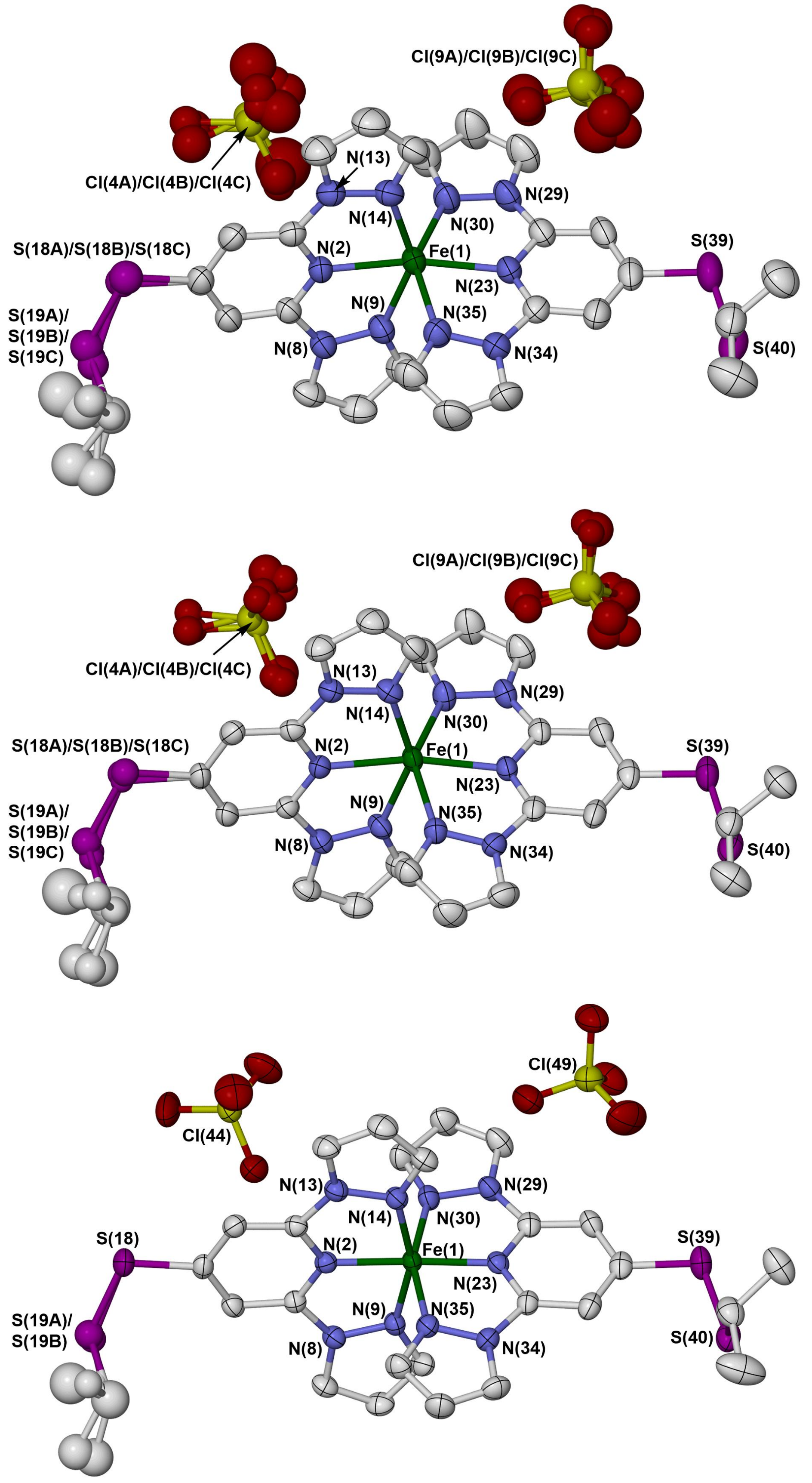


**Figure S15** The asymmetric unit of **1**$[ClO_4]_2$·sf. Top, high-spin at 250 K; center, high-spin at 170 K; bottom, low-spin at 170 K. Other details as for Figure S3, which also shows the full atom numbering scheme for the structure. Color code: C, white; Cl, yellow; Fe, green; N, blue; O, red; S, purple.

The Figure continues on the next page.

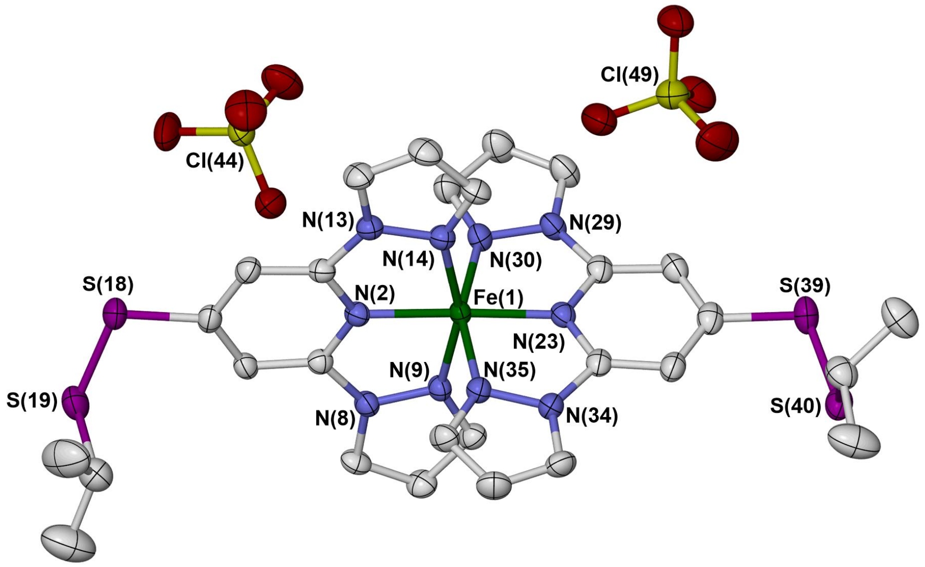


**Figure S15 continued.** This page; low-spin at 110 K.

Color code: C, white; Cl, yellow; Fe, green; N, blue; O, red; S, purple.

**Table S7** Selected bond lengths, angles and other structural parameters (Å , °, Å$^3$) for the solvent-free crystals **1X$_2$**·sf (X$^-$ = $BF_4^-$ or $ClO_4^-$). See Figures S1-S3 for the atom numbering scheme, while definitions of $V_{Oh}$, $\Sigma$, $\Theta$, $\varphi$ and $\theta$ are given on page S16.

| | **1[BF$_4$]$_2$**·sf,, experiment 1[a] | | **1[BF$_4$]$_2$**·sf,, experiment 2[a] | | **1[ClO$_4$]$_2$**·sf[b] | | | |
|---|---|---|---|---|---|---|---|---|
| *T* / K | 100 | 250 | 100 | 250 | 110 | 170, warming | 170, cooling | 250 |
| Fe(1)–N(2) | 2.1261(19) | 2.131(3) | 1.905(3) | 2.133(3) | 1.911(3) | 1.912(3) | 2.123(4) | 2.126(3) |
| Fe(1)–N(9) | 2.184(2) | 2.187(3) | 1.976(3) | 2.191(4) | 1.979(3) | 1.983(3) | 2.181(4) | 2.187(3) |
| Fe(1)–N(14) | 2.183(2) | 2.187(3) | 1.988(4) | 2.189(4) | 1.996(3) | 2.000(3) | 2.187(4) | 2.185(3) |
| Fe(1)–N(23) | 2.140(2) | 2.137(3) | 1.910(3) | 2.136(3) | 1.902(3) | 1.907(3) | 2.133(4) | 2.142(3) |
| Fe(1)–N(30) | 2.160(2) | 2.168(3) | 1.986(4) | 2.170(4) | 1.979(3) | 1.983(3) | 2.159(4) | 2.169(3) |
| Fe(1)–N(35) | 2.209(2) | 2.211(3) | 1.984(3) | 2.212(4) | 1.985(3) | 1.989(3) | 2.195(4) | 2.202(3) |
| | | | | | | | | |
| N(2)–Fe(1)–N(9) | 73.44(7) | 72.94(10) | 79.95(14) | 73.09(12) | 79.64(11) | 79.47(12) | 73.47(14) | 73.07(12) |
| N(2)–Fe(1)–N(14) | 73.58(7) | 73.26(10) | 79.60(14) | 73.18(13) | 79.60(11) | 79.49(12) | 73.34(14) | 73.11(12) |
| N(2)–Fe(1)–N(23) | 164.11(8) | 165.07(10) | 177.14(14) | 165.15(13) | 177.76(12) | 177.61(13) | 166.37(14) | 166.37(12) |
| N(2)–Fe(1)–N(30) | 122.66(8) | 121.74(11) | 103.05(14) | 121.73(13) | 102.51(11) | 102.78(13) | 119.70(14) | 120.19(13) |
| N(2)–Fe(1)–N(35) | 91.55(7) | 92.50(10) | 97.35(14) | 92.53(12) | 98.14(11) | 98.09(13) | 93.70(14) | 93.79(12) |
| N(9)–Fe(1)–N(14) | 146.76(8) | 145.78(10) | 159.55(14) | 145.85(12) | 159.24(11) | 158.96(13) | 146.58(15) | 145.89(13) |
| N(9)–Fe(1)–N(23) | 104.58(7) | 105.75(10) | 100.31(14) | 105.47(13) | 99.78(11) | 99.95(13) | 104.42(14) | 104.84(12) |
| N(9)–Fe(1)–N(30) | 99.87(8) | 99.08(11) | 94.36(14) | 99.18(14) | 94.20(11) | 94.23(13) | 98.30(15) | 98.22(14) |
| N(9)–Fe(1)–N(35) | 85.97(8) | 87.09(11) | 88.27(14) | 86.87(14) | 88.96(11) | 88.97(13) | 87.73(14) | 87.89(13) |
| N(14)–Fe(1)–N(23) | 108.26(7) | 108.27(10) | 100.11(14) | 108.50(13) | 100.96(11) | 101.08(13) | 108.74(14) | 109.08(12) |
| N(14)–Fe(1)–N(30) | 94.33(8) | 94.07(11) | 90.15(14) | 93.95(14) | 90.36(12) | 90.36(13) | 94.69(15) | 94.81(14) |
| N(14)–Fe(1)–N(35) | 98.96(7) | 99.57(11) | 94.43(14) | 99.79(14) | 93.88(11) | 94.03(13) | 98.13(14) | 98.66(13) |
| N(23)–Fe(1)–N(30) | 73.20(8) | 73.18(11) | 79.79(14) | 73.10(13) | 79.68(12) | 79.55(13) | 73.84(15) | 73.35(13) |
| N(23)–Fe(1)–N(35) | 72.57(8) | 72.58(10) | 79.82(14) | 72.62(13) | 79.67(12) | 79.57(13) | 72.70(14) | 72.61(13) |
| N(30)–Fe(1)–N(35) | 145.64(8) | 145.60(11) | 159.58(14) | 145.57(14) | 159.35(12) | 159.12(13) | 146.45(15) | 145.85(13) |
| | | | | | | | | |
| $V_{Oh}$ | 12.146(7) | 12.199(10) | 9.670(11) | 12.225(13) | 9.683(9) | 9.725(10) | 12.183(14) | 12.231(12) |
| $\Sigma$ | 161.5(3) | 161.9(4) | 92.3(5) | 162.3(5) | 92.3(4) | 93.5(4) | 156.6(5) | 159.6(4) |
| $\Theta$ | 508 | 517 | 292 | 517 | 296 | 300 | 502 | 514 |
| $\phi$ | 164.11(8) | 165.07(10) | 177.14(14) | 165.15(13) | 177.76(12) | 177.61(13) | 166.37(14) | 166.37(12) |
| $\theta$ | 81.65(2) | 82.05(4) | 83.99(4) | 82.11(4) | 84.15(3) | 84.13(3) | 82.98(4) | 83.09(4) |

[a]This compound is high-spin at 250 K. The crystal retained its high-spin form the first time it was cooled to 100 K, but transformed to its low-spin state when it was rewarmed and cooled a second time. That reflects a kinetic barrier to spin-crossover when the crystal is cooled too rapidly – see the main article for more details.
[b]This crystal undergoes an abrupt spin-transition at $T_{½}$ = 175 K with a thermal hysteresis width $\Delta T_{½}$ = 20 K. The crystal was measured in both its high-spin and low-spin forms at the same temperature inside the hysteresis loop, 170 K.

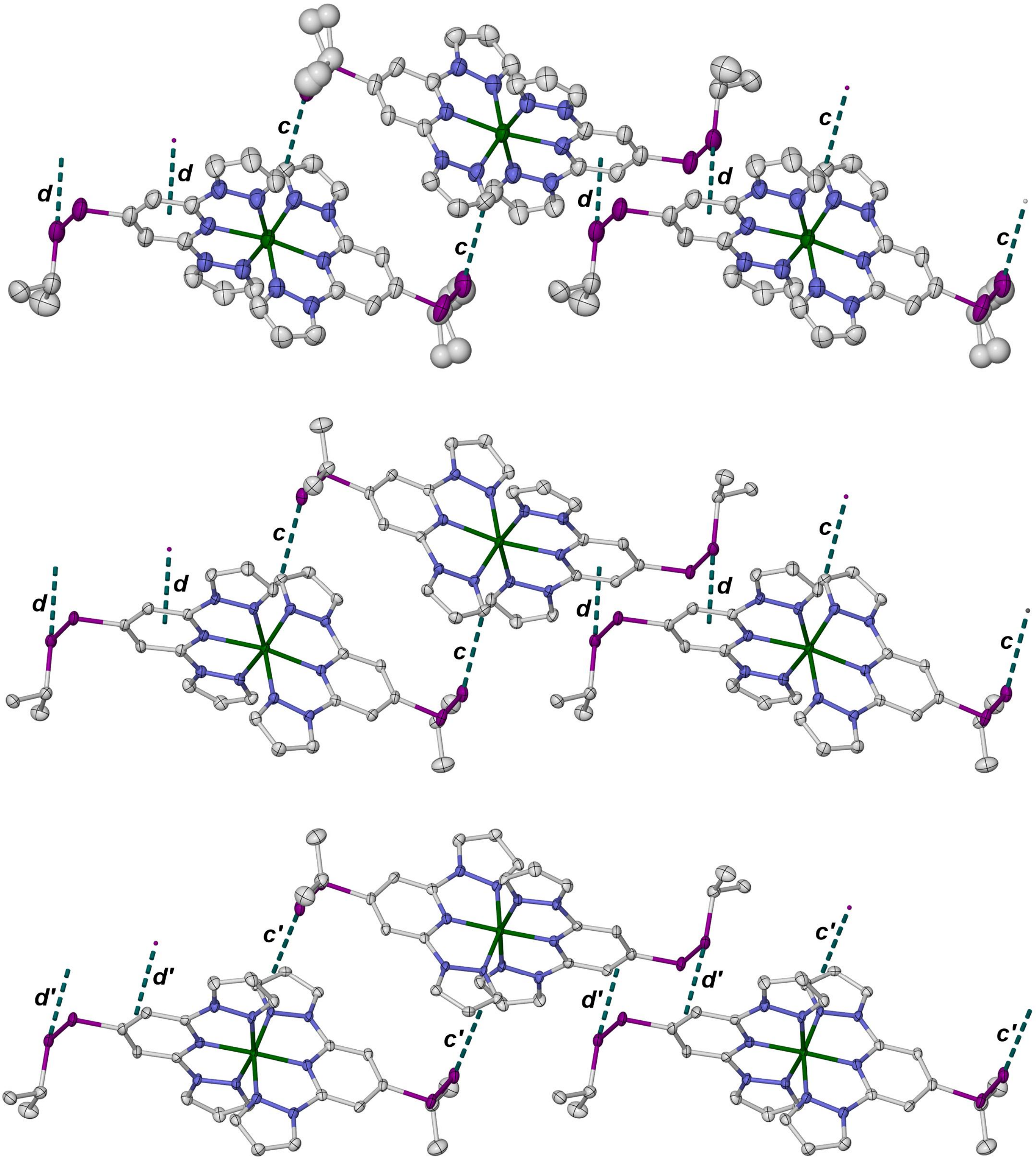


**Figure S16** Intermolecular $n\cdots\pi$ contacts in **1[BF$_4$]$_2$**·sf, showing the association of the cations into 1D chains: top, high-spin at 250 K; middle, high-spin at 100 K; bottom, low-spin at 100 K. Color code: C, white; Fe, green; N, blue; S, purple.

The $\beta$-S atom from each *iso*propyldisulfanyl substituent is involved in one of these interactions. S(19) overlies the centroid of a C{pyridyl}−N{pyrazolyl} bond at 250 K, but is closer to the pyridyl C atom at 120 K. S(40) overlies a pyrazolyl ring in its nearest neighbor, but is oriented above the ring centroid; a C−C bond in the ring; or a specific C atom in different structures. The interactions are:

***c***: S(19)···C($10^{v}$) or ***c'***: S(19)···[C($10^{vi}$)−C($11^{vi}$)] – symmetry code (vi) 1−*x*, −*y*, 1−*z*.

***d***: S(40)···[N($23^{i}$)−C($28^{i}$)]; ***d'***: S(39)···[C($25^{i}$)−C($26^{i}$)] or ***d''***: S(40)···C($25^{i}$) – symmetry code (i) 1−*x*, 1−*y*, 1−z.

The distances of these weak interactions are listed in the following Table.

**Table S8** Intermolecular $n \cdots \pi$ contacts for **1X$_2$**·sf ($X^-$ = $BF_4^-$ or $ClO_4^-$; Figure S16). Errors are not given for distances to centroids of bonds or aromatic rings.

| Distance | ***c/c'*** | ***d/d'*** |
|---|---|---|
| **1[BF$_4$]$_2$**·sf 100 K, low-spin | 3.456 (***c'***) | 3.313 (***d'***) |
| **1[BF$_4$]$_2$**·sf 100 K, high-spin | 3.499(2) (***c***) | 3.329 (***d***) |
| **1[BF$_4$]$_2$**·sf, 250 K | 3.525(4) (***c***) | 3.359 (***d***) |
| | | |
| **1[ClO$_4$]$_2$**·sf 110 K | 3.436 (***c'***) | 3.323(3) (***d''***) |
| **1[ClO$_4$]$_2$**·sf 170 K, low-spin | 3.384/3.519 (***c'***)[a] | 3.323(4) (***d''***) |
| **1[ClO$_4$]$_2$**·sf 170 K, high-spin | 3.253(7)/3.542(6)/3.638(6) (***c***)[a] | 3.397 (***d***) |
| **1[ClO$_4$]$_2$**·sf, 250 K | 3.264(6)/3.595(6)/3.676(5) (***c***)[a] | 3.409 (***d***) |

[a]The S atom of this *iso*propyldisulfanyl group is disordered at this temperature.

The sum of the Pauling van der Waals radii of an S atom and an aromatic ring is 3.55 Å.[18] Hence, contact ***d*** corresponds to a strongly attractive intermolecular interaction in all these structures, but ***c*** is longer and may just corresponds to a van der Waals contact.

**Table S9** Variable temperature unit cell parameters for **1[BF$_4$]$_2$**·sf. The unit cell dimensions are plotted in the main article, Figure 3.

| *T* (K) | *a* (Å) | *b* (Å) | *c* (Å) | *β* (°) | *V* (Å$^3$) |
|---|---|---|---|---|---|
| Cooling | | | | | |
| 250 | 12.7166(14) | 18.8646(17) | 16.0244(16) | 101.122(9) | 3772.0(6) |
| 220 | 12.6792(18) | 18.8347(18) | 15.9778(18) | 101.055(11) | 3744.8(8) |
| 200 | 12.6571(16) | 18.8160(15) | 15.9579(16) | 100.992(9) | 3730.8(7) |
| 190 | 12.6452(19) | 18.8107(17) | 15.9436(18) | 100.931(10) | 3723.6(8) |
| 180 | 12.6387(16) | 18.805(2) | 15.9287(16) | 100.948(9) | 3717.0(7) |
| 170 | 12.625(2) | 18.810(2) | 15.910(2) | 100.866(12) | 3710.5(9) |
| 160 | 12.624(2) | 18.788(2) | 15.898(2) | 100.920(12) | 3702.2(9) |
| 150 | 12.619(3) | 18.781(3) | 15.884(2) | 100.890(13) | 3696.8(11) |
| 140 | 12.621(3) | 18.706(3) | 15.966(4) | 101.118(15) | 3698.4(12) |
| 130 | 12.626(14) | 18.774(17) | 15.877(13) | 101.26(10) | 3691(6) |
| 125 | 12.296(4) | 19.123(5) | 15.894(3) | 103.58(2) | 3632.6(16) |
| 120 | 12.309(3) | 19.107(3) | 15.906(2) | 103.651(14) | 3635.0(11) |
| 115 | 12.265(4) | 19.117(6) | 15.897(4) | 103.53(2) | 3623.8(19) |
| 110 | 12.294(3) | 19.091(3) | 15.890(2) | 103.666(14) | 3623.9(11) |
| 100 | 12.289(3) | 19.092(4) | 15.876(2) | 103.643(14) | 3619.7(12) |
| Warming | | | | | |
| 110 | 12.288(2) | 19.090(2) | 15.8845(17) | 103.615(10) | 3621.56(8) |
| 115 | 12.293(2) | 19.101(3) | 15.899(17) | 103.653(12) | 3627.7(9) |
| 120 | 12.2898(19) | 19.106(3) | 15.8837(15) | 103.561(10) | 3625.6(8) |
| 125 | 12.284(3) | 19.098(4) | 15.904(2) | 103.561(15) | 3627.1(12) |
| 130 | 12.2965(19) | 19.119(3) | 15.9171(18) | 103.615(11) | 3636.8(9) |
| 140 | 12.315(2) | 19.091(2) | 15.938(18) | 103.700(10) | 3640.4(8) |
| 150 | 12.3160(18) | 19.1191(19) | 15.9418(16) | 103.728(10) | 3646.6(7) |
| 160 | 12.296(9) | 19.129(10) | 15.925(8) | 103.39(6) | 3644(4) |
| 170 | 12.6293(16) | 18.796(2) | 15.9174(15) | 100.944(8) | 3709.8(7) |
| 180 | 12.6433(18) | 18.8011(19) | 15.9301(15) | 100.951(10) | 3717.8(7) |
| 190 | 12.6516(18) | 18.806(2) | 15.9458(19) | 100.986(11) | 3724.4(8) |
| 200 | 12.664(2) | 18.818(3) | 15.961(2) | 101.011(12) | 3733.7(10) |
| 220 | 12.677(2) | 18.828(3) | 15.987(2) | 101.064(12) | 3744.9(9) |
| 250 | 12.718(3) | 18.852(4) | 16.025(3) | 101.141(16) | 3769.5(14) |

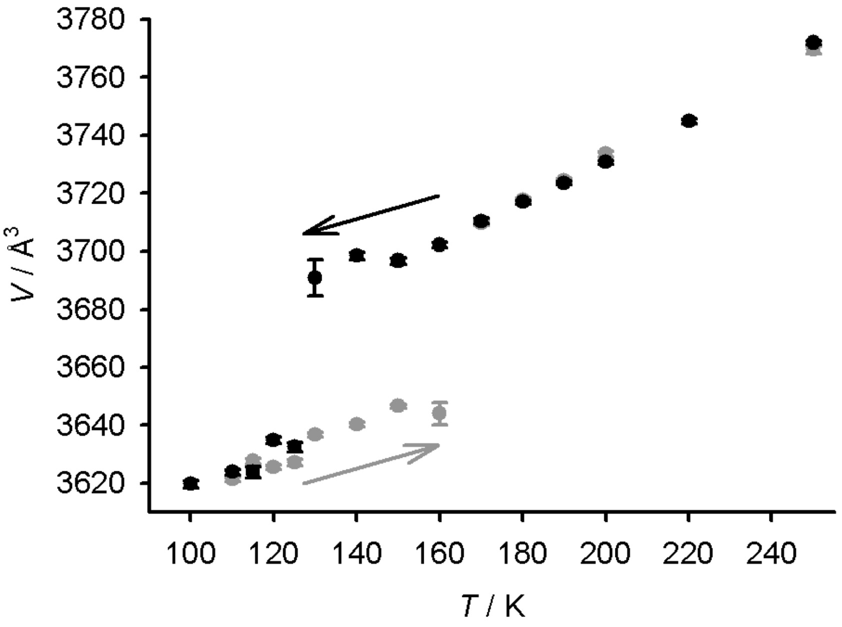


**Figure S17** Variable temperature unit cell volumes for **1[BF$_4$]$_2$**·sf, measured with cooling (black) and warming (gray) temperature ramps. Error bars are mostly smaller than the symbols on the graph.

**Table S10** Variable temperature unit cell parameters for **1[ClO$_4$]$_2$**·sf.

| *T* (K) | *a* (Å) | *b* (Å) | *c* (Å) | *β* (°) | *V* (Å$^3$) |
|---|---|---|---|---|---|
| Cooling | | | | | |
| 250 | 12.8000(3) | 19.0750(4) | 16.1822(4) | 102.365(3) | 3859.40(16) |
| 220 | 12.765(2) | 19.068(5) | 16.136(3) | 102.36(2) | 3836.4(15) |
| 200 | 12.7443(7) | 19.0693(7) | 16.1032(7) | 102.178(5) | 3825.4(3) |
| 190 | 12.7254(6) | 19.0634(6) | 16.0951(9) | 102.139(5) | 3817.2(3) |
| 180 | 12.7182(6) | 19.0696(10) | 16.0822(13) | 102.125(6) | 3813.4(4) |
| 170 | 12.6914(12) | 19.0134(7) | 16.0551(9) | 102.114(7) | 3787.9(4) |
| 160 | 12.4066(5) | 19.2888(5) | 16.0439(6) | 103.812(4) | 3728.4(2) |
| 150 | 12.4054(5) | 19.2998(11) | 16.0355(4) | 103.850(4) | 3727.6(3) |
| 130 | 12.3916(7) | 19.2819(11) | 16.0127(5) | 103.858(5) | 3714.6(3) |
| 120 | 12.420(2) | 19.304(7) | 16.016(3) | 103.96(2) | 3726.5(18) |
| Warming | | | | | |
| 130 | 12.389(11) | 19.2976(9) | 16.0162(4) | 103.849(4) | 3717.9(4) |
| 150 | 12.4088(7) | 19.3182(10) | 16.0446(7) | 103.866(6) | 3734.0(3) |
| 160 | 12.4030(5) | 19.2988(4) | 16.0416(5) | 103.857(3) | 3728.0(2) |
| 170 | 12.4194(3) | 19.3417(13) | 16.0733(9) | 103.838(4) | 3748.9(3) |
| 180 | 12.4254(6) | 19.3009(18) | 16.0516(11) | 103.728(6) | 3739.6(5) |
| 190 | 12.7199(7) | 19.0537(9) | 16.0954(4) | 102.188(4) | 3813.0(3) |
| 200 | 12.7450(4) | 19.0716(11) | 16.1195(13) | 102.192(5) | 3829.8(4) |
| 220 | 12.7604(8) | 19.0850(8) | 16.1314(14) | 102.212(7) | 3839.6(4) |

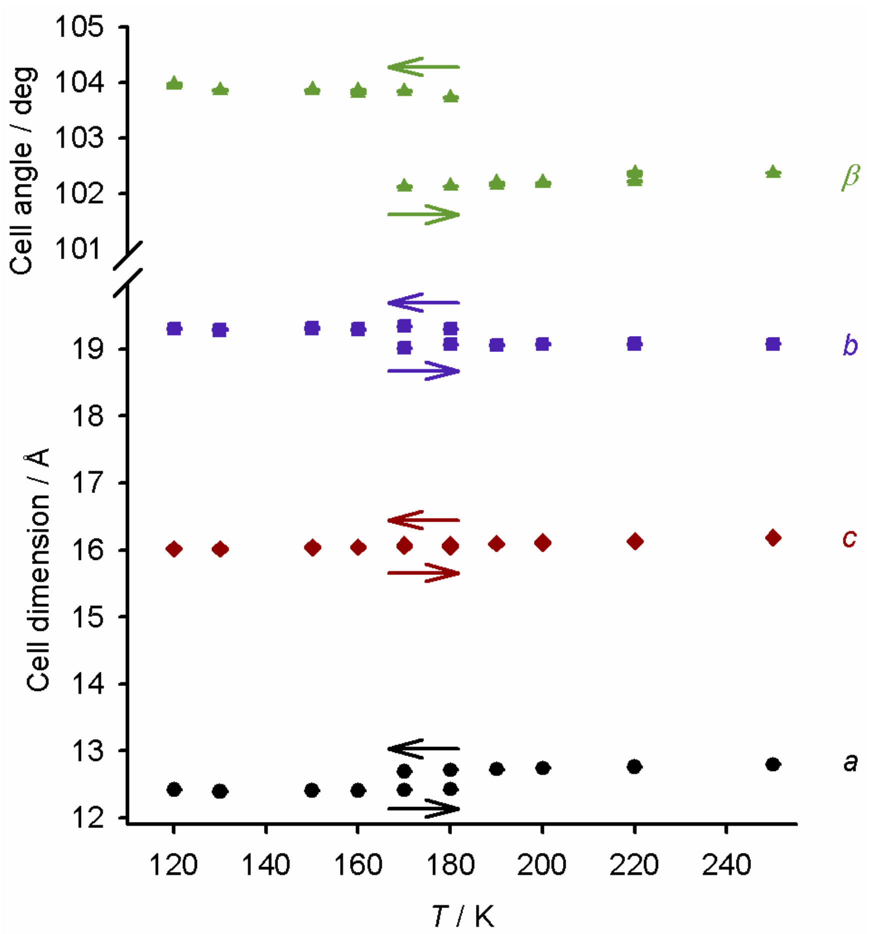


**Figure S18** Variable temperature unit cell dimensions for **1[ClO$_4$]$_2$**·sf (Table S10). Data were collected in cooling and warming temperature ramps. Thermal hysteresis in the spin-transition is visible in *a*, *b* and *β*. Error bars are shown, but are mostly smaller than the symbols on the graph.

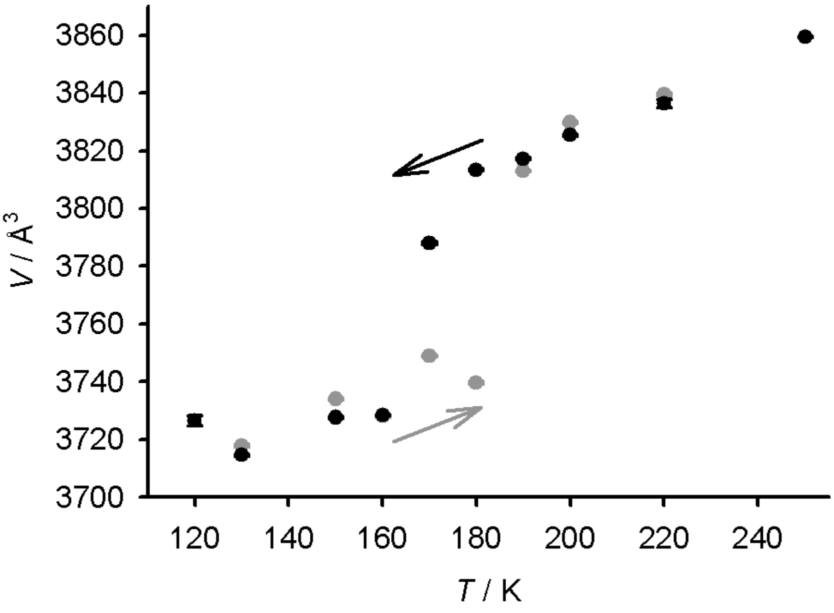


**Figure S19** Variable temperature unit cell volumes for **1[ClO$_4$]$_2$**·sf, measured with cooling (black) and warming (gray) temperature ramps.

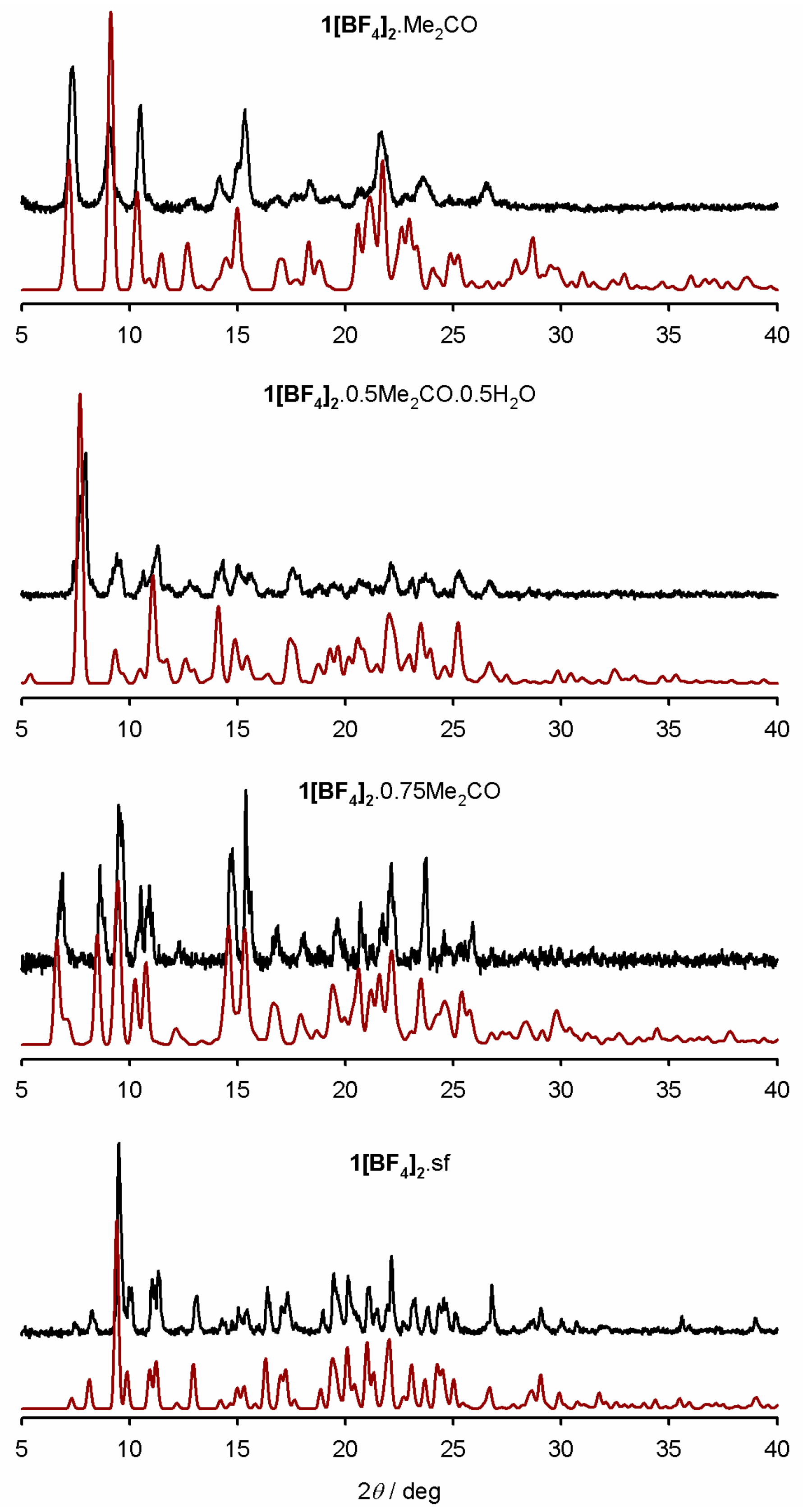


**Figure S20** Measured (black) and simulated (red) X-ray powder diffraction patterns for acetone solvates of **1[BF$_4$]$_2$**, and the annealed solvent-free material.

These data are discussed on the following page.

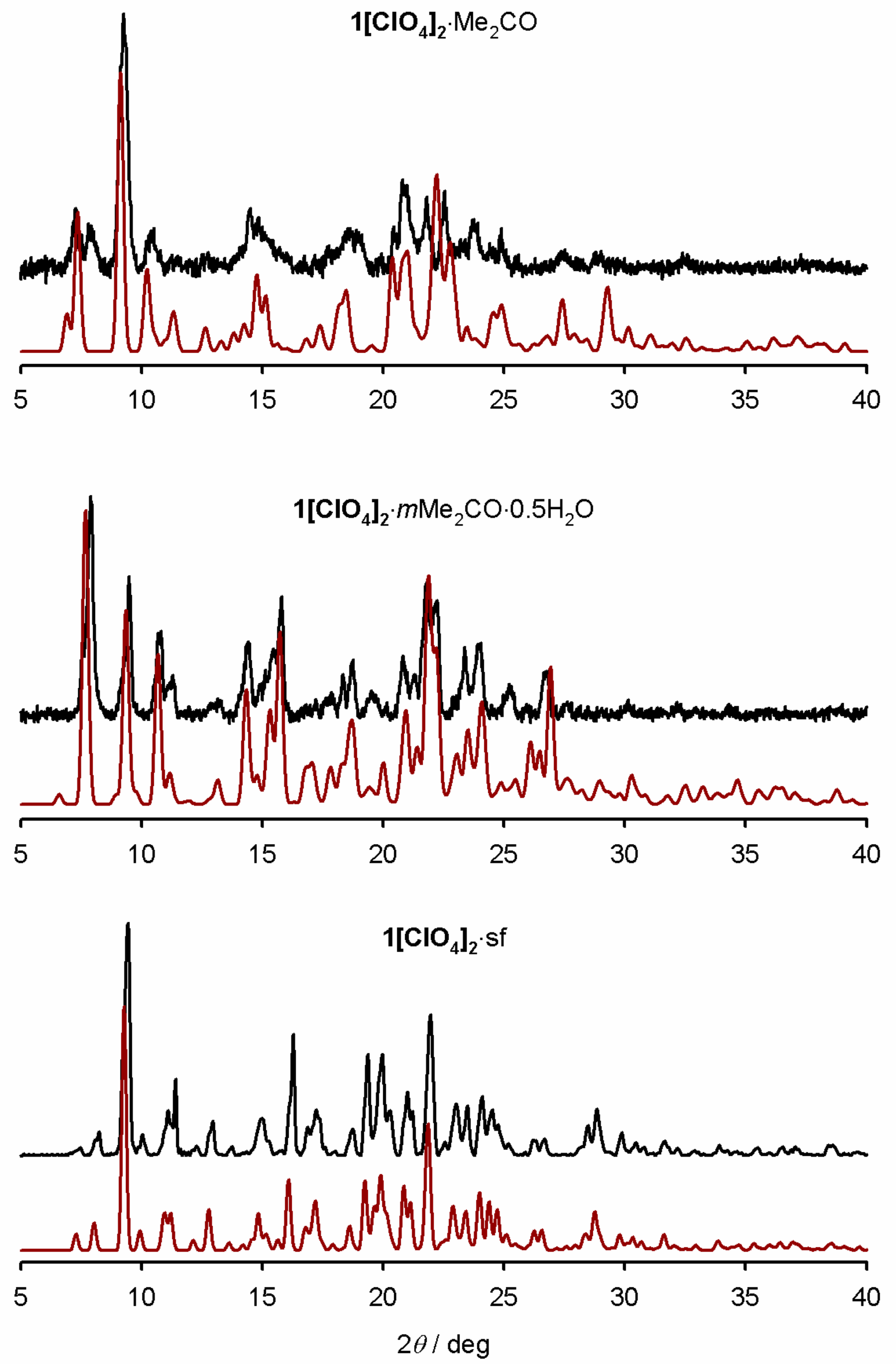


**Figure S21** Measured (black) and simulated (red) X-ray powder diffraction patterns for acetone solvates of **1[ClO$_4$]$_2$**, and the annealed solvent-free material.

The acetone solvates of each salt in Figures S20 and S21 crystallize together, and were isolated by Pasteur separation for these measurements. Those samples had to be coated in nujol before measurement, to protect against solvent loss, which will contribute towards the lower signal-to-noise ratio in those powder patterns.

The samples of **1[BF$_4$]$_2$**·sf and **1[ClO$_4$]$_2$**·sf were prepared by annealing mixtures of the acetone solvate phases at 370 K for 1 hr. Those samples were exposed to air during the measurement.

The powder patterns of **1[BF$_4$]$_2$**·Me$_2$CO and **1[ClO$_4$]$_2$**·Me$_2$CO generally resemble their simulations but are both broadened, which may indicate partial desolvation of the materials during sample preparation. Facile loss of lattice solvent was also observed during magnetic measurements of these compounds.

The agreement between experiment and simulation for the other phases in the Figures is excellent.

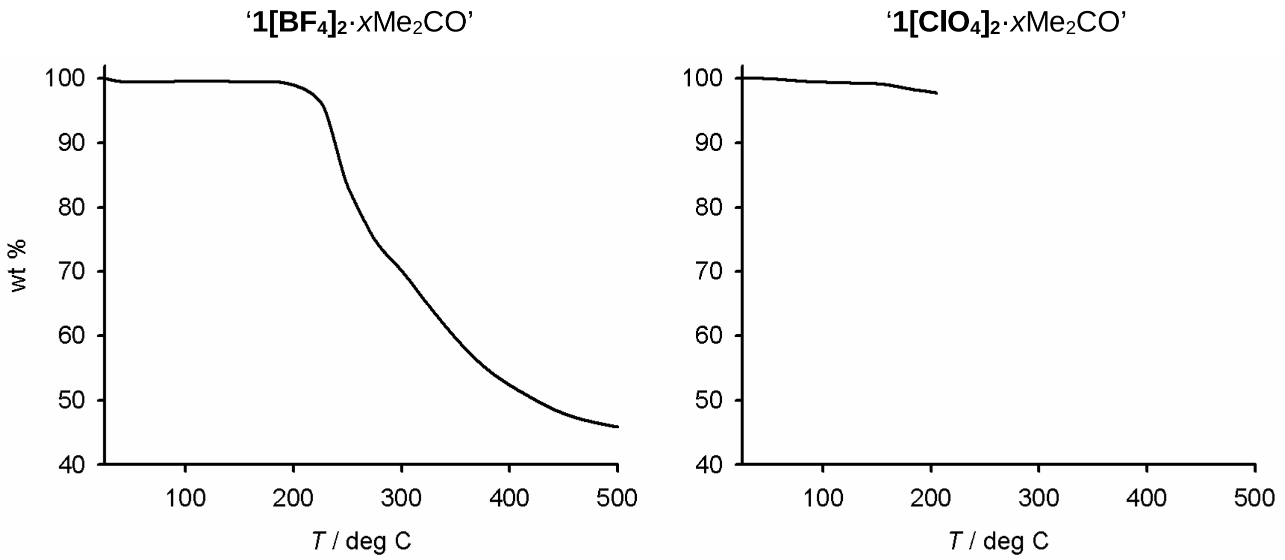


**Figure S22** Thermogravimetric analyses for the '**1[BF$_4$]$_2$**·*x*Me$_2$CO' and '**1[ClO$_4$]$_2$**·*x*Me$_2$CO' mixed acetone solvate powders. The perchlorate salt was only heated to 200 °C, because of its risk of detonation at higher temperatures.

Unlike the powder diffraction measurements, it was not possible to protect TGA samples against solvent loss. The absence of a significant mass loss below 200 °C implies these samples had lost their lattice solvent by the time the measurement was started. Hence, these are essentially TGAs of the **1X$_2$**·sf phases.

The other solvates of **1[BF$_4$]$_2$** and **1[ClO$_4$]$_2$** are more stable to solvent loss by TGA (Figure S31).

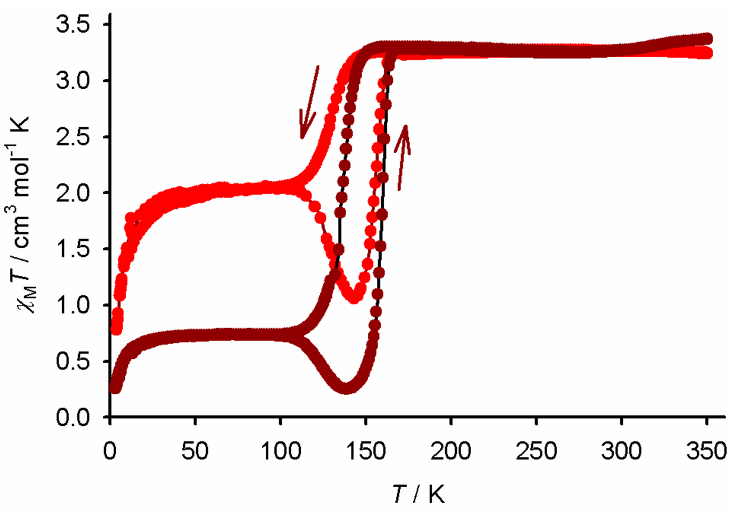


**Figure S23** Magnetic susceptibility data for **1[BF$_4$]$_2$**·sf, at a scan rate of 5 K min$^{-1}$ (dark red) and 10 K min$^{-1}$ (pale red). The 5 K min$^{-1}$ data are the same as in Figure 1 of the main article.

The SCO becomes less complete, and the relaxation step is more pronounced, at the faster scan rate. That is characteristic of kinetic trapping of a fraction of the sample in their metastable high-spin form upon cooling. That has also been observed in some other [Fe(bpp$^R$)$_2$]$^{2+}$ derivatives showing abrupt spin transitions close to 100 K.[15,19]

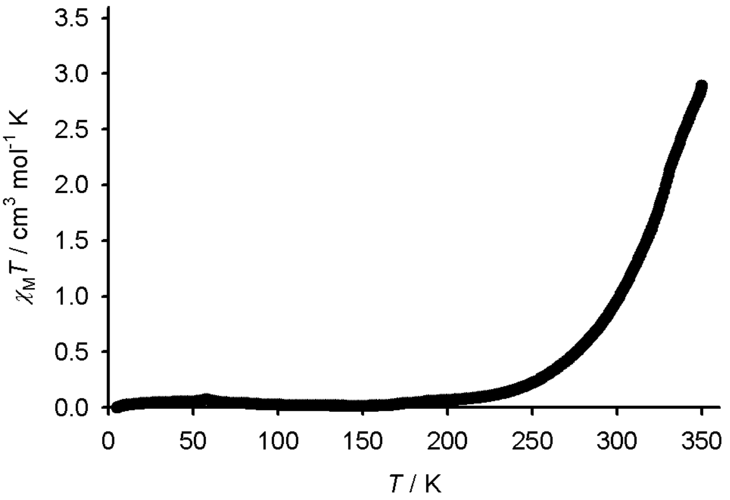


**Figure S24** Magnetic susceptibility data for a phase-pure sample of **1[ClO$_4$]$_2$**·$m$Me$_2$CO·0.5H$_2$O. The data were measured in warming mode, at scan rate 5 K min$^{-1}$.

This sample was purified from a recrystallized sample of '**1[ClO$_4$]$_2$**·$x$Me$_2$CO' by a Pasteur separation. The lack of any abrupt features between 150-170 K implies the material is phase-pure (Figure S24). This is the only magnetic measurement we obtained from a purified acetone solvate phase, which is consistent with the spin state of its crystal structure.

The reversibility of the SCO was not examined in this measurement, so solvent loss might be contributing to the high temperature behavior. Consistent with that, the sample had decomposed when its powder pattern was measured after this experiment.

Be that as it may, the gradual SCO above 200 K is clear in these data, which is also consistent with the higher temperature behavior of the mixed-phase '**1[ClO$_4$]$_2$**·$x$Me$_2$CO' material in Figure S25.

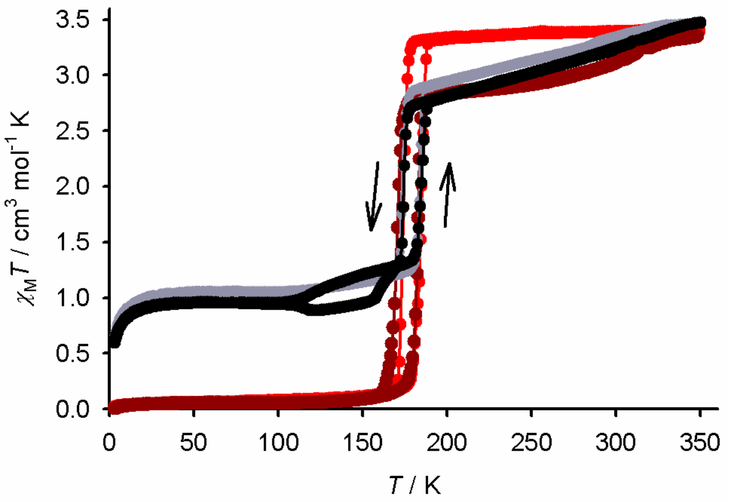


**Figure S25** Magnetic susceptibility measurement for a typical mixed-phase sample of '**1[ClO$_4$]$_2$**·$x$Me$_2$CO': (i) first cycle, 300→3→350 K (black); (ii) second cycle, 350→3→350 K (gray); (iii) third cycle, 350→3→350 K (dark red); (iv) fourth cycle, 350→3→300 K (pale red). Data points are connected by spline curves for clarity. Scan rate 5 K min$^{-1}$.

The abrupt hysteretic SCO at 170 K in the first scan corresponds to **1[ClO$_4$]$_2$**·sf; there is no **1[ClO$_4$]$_2$**·Me$_2$CO visible in these data (Figure 2, main article). We propose conversion of **1[ClO$_4$]$_2$**·Me$_2$CO to **1[ClO$_4$]$_2$**·sf took place during preparation of the SQUID sample. The weak, hysteretic features between 120-160 K have vanished in the second scan, and may represent a residue of partly desolvated **1[ClO$_4$]$_2$**·Me$_2$CO (*cf* Figure 1, main article).

The rapid loss of solvent before the measurement was a common problem with the acetone solvates in this work. Figure 2 shows the only time in four attempts that the clean conversion of **1[ClO$_4$]$_2$**·Me$_2$CO to **1[ClO$_4$]$_2$**·sf was observed inside the magnetometer. We never did achieve this for **1[BF$_4$]$_2$**·Me$_2$CO.

The gradual SCO feature above 250 K in scans (i)-(iii) is consistent with the **1[ClO$_4$]$_2$**·$m$Me$_2$CO·0.5H$_2$O phase.

No single crystals that are high-spin below 150 K were isolated from this mixture. Hence, the residual low-temperature magnetic moment in scans (i) and (ii) implies freshly prepared '**1[ClO$_4$]$_2$**·$x$Me$_2$CO' might also contain a third phase. That could be related to **1[BF$_4$]$_2$**·0.75Me$_2$CO, which has a mixed spin-state population at low temperature.

Conversion of the whole sample to **1[ClO$_4$]$_2$**·sf is complete in the fourth scan. The temperature of the hysteretic spin-transition in the final material is consistent with the phase-pure sample in Figure 2, and with the single crystal unit cell data from **1[ClO$_4$]$_2$**·sf.

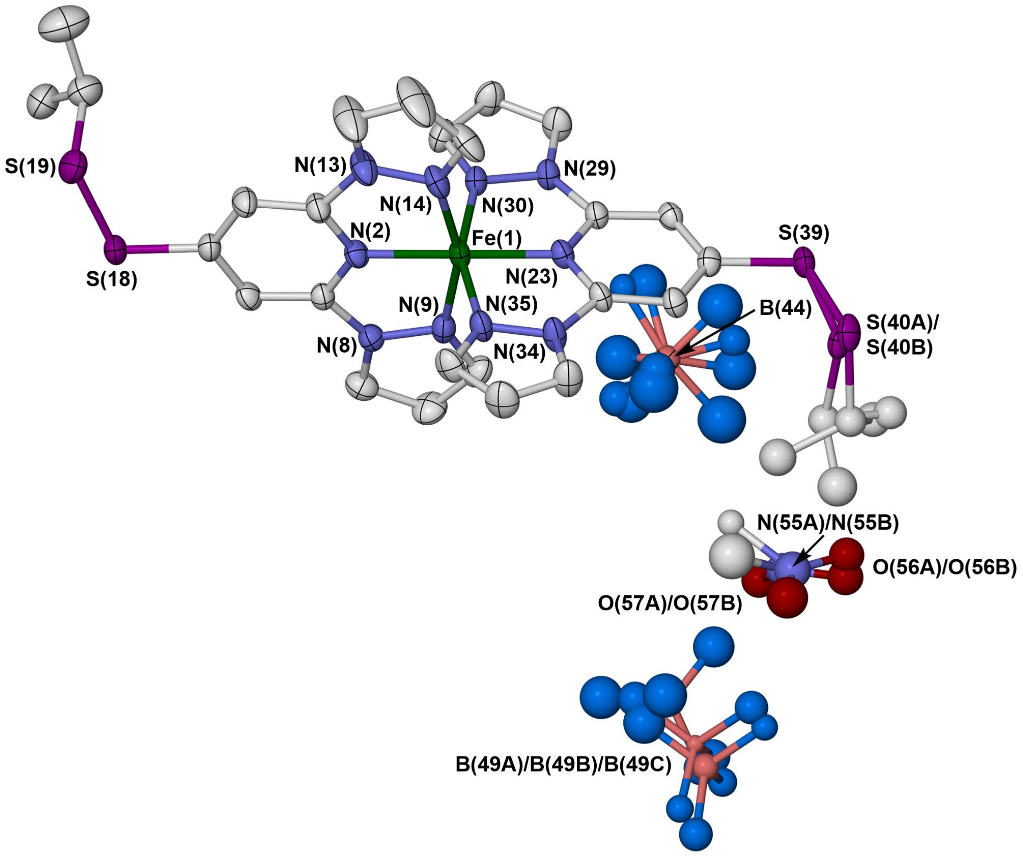


**Figure S26** The asymmetric unit of **1[BF$_4$]$_2$**·MeNO$_2$ at 120 K. Other details as for Figure S3, which also shows the full atom numbering scheme for the structure. Color code: C, white; B, pink; F, cyan; Fe, green; N, blue; O, red; S, purple.

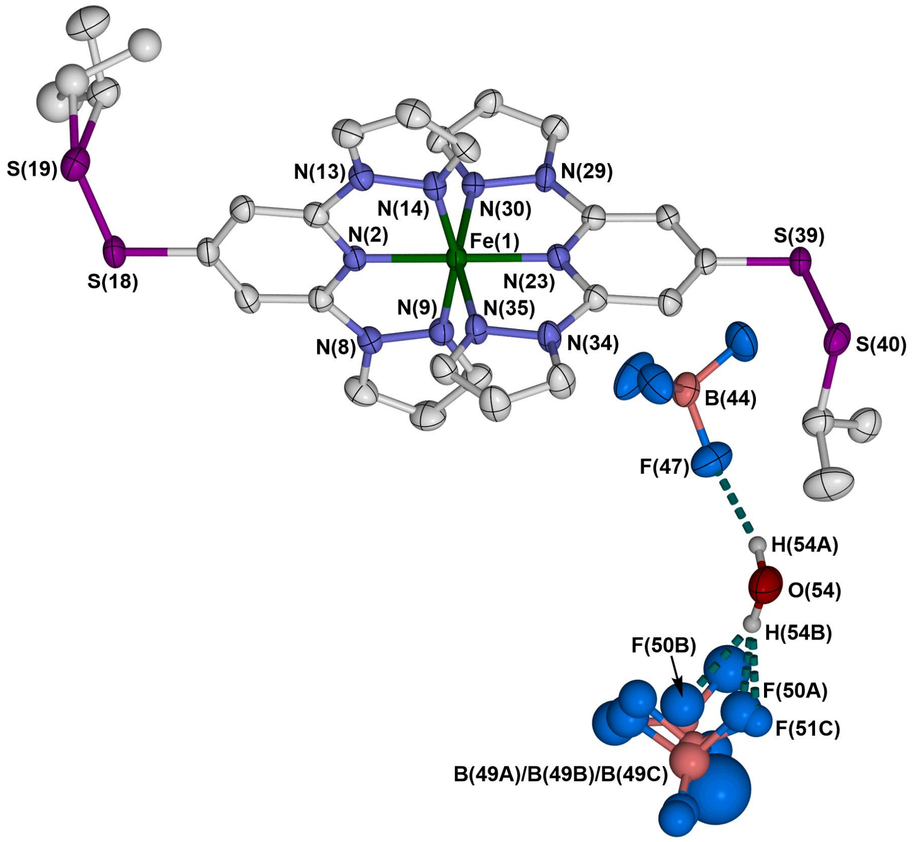


**Figure S27** The asymmetric unit of **1[BF$_4$]$_2$**·$H_2O$ at 120K. The full atom numbering in the structure corresponds to that in Figure S3. The H atoms on the lattice water molecule are included in the figure; other details as for Figure S3. Color code: C, white; B, pink; F, cyan; Fe, green; N, blue; O, red; S, purple.

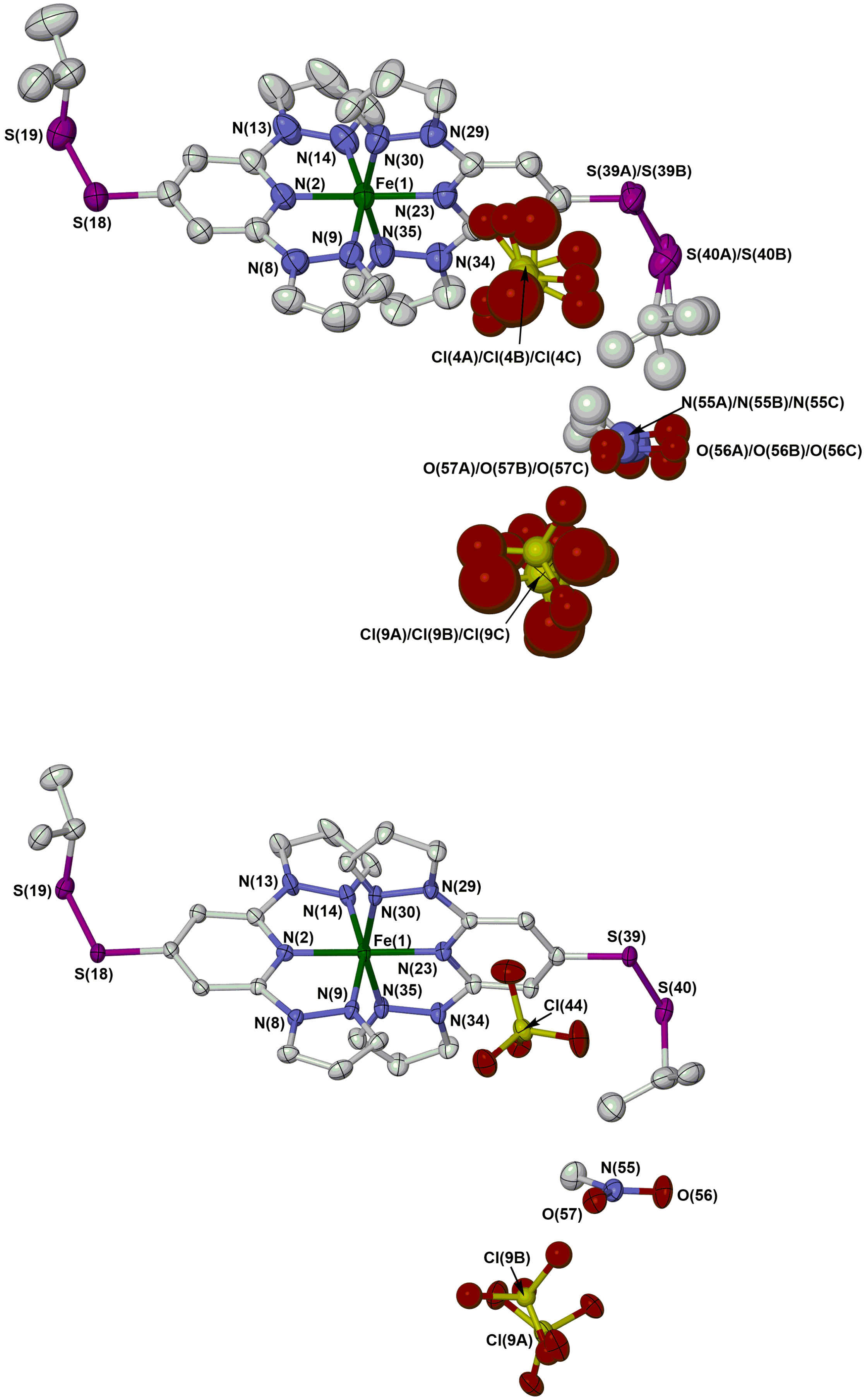


**Figure S28** The asymmetric unit of **1[ClO$_4$]$_2$**·*n*MeNO$_2$. Top, mostly low-spin at 250 K; bottom, fully low-spin at 120 K. Other details as for Figure S3, which also shows the full atom numbering scheme for the structure. Color code: C, white; Cl, yellow; Fe, green; N, blue; O, red; S, purple.

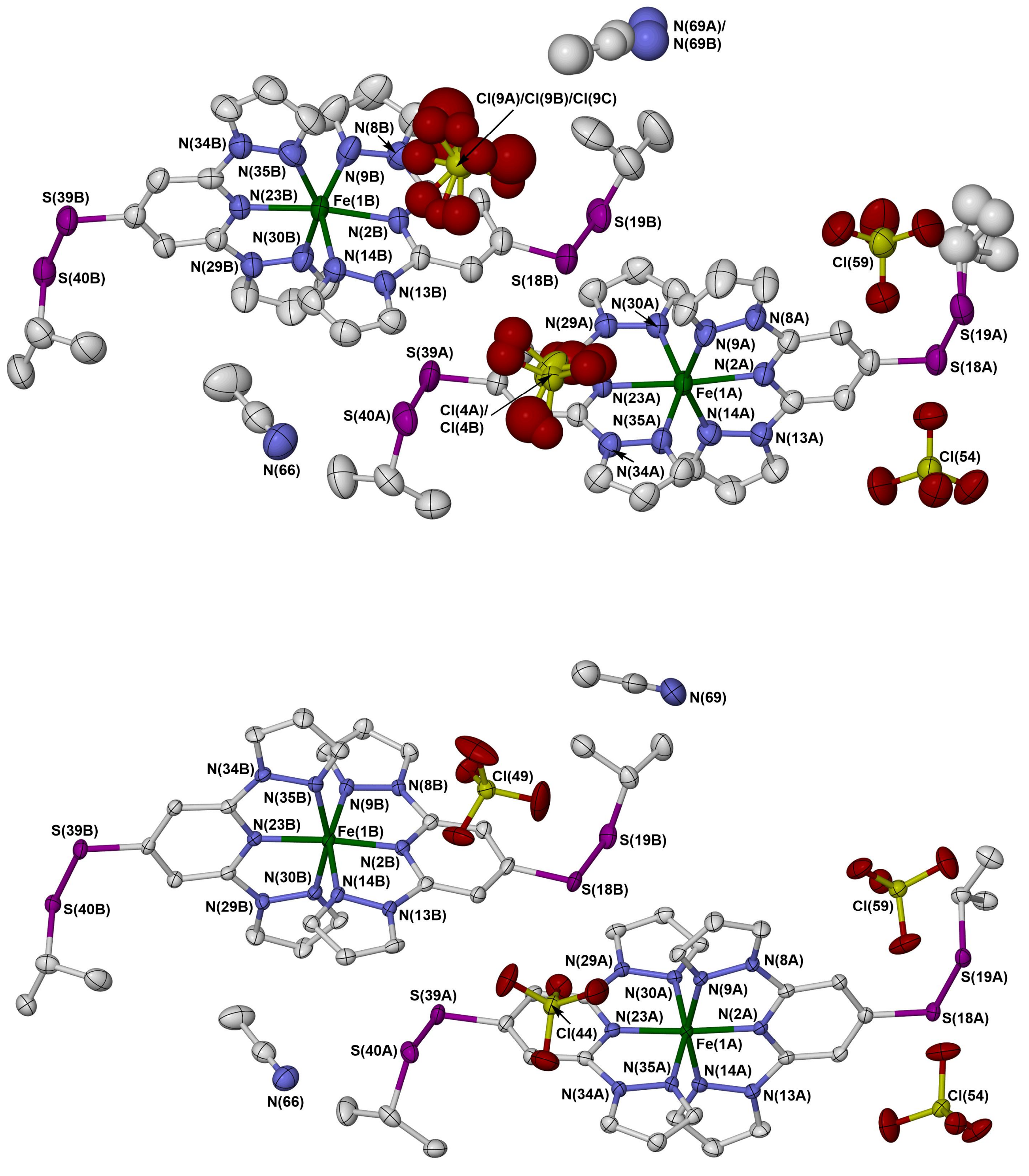


**Figure S29** The asymmetric unit of **1[ClO$_4$]$_2$**·MeCN. Top, mostly high-spin at 250 K; bottom, low-spin at 120 K. The full atom numbering in the structures corresponds to that in Figure S3, with suffixes to distinguish between cations A and B in the model. Other details as for Figure S3. Color code: C, white; Cl, yellow; Fe, green; N, blue; O, red; S, purple.

**Table S11** Selected bond lengths, angles and other structural parameters (Å , °, Å$^3$) for the other solvate crystals of **1X₂** → $\mathbf{1X_2}$ ($X^-$ = $BF_4^-$ or $ClO_4^-$). See Figures S26-S29 for the atom numbering scheme, while definitions of $V_{Oh}$, $\Sigma$, $\Theta$, $\varphi$ and $\theta$ are given on page S16.

| | $\mathbf{1[BF_4]_2}$·$MeNO_2$ | $\mathbf{1[BF_4]_2}$·$H_2O$ | $\mathbf{1[ClO_4]_2}$·$n$$MeNO_2$ ($n \approx 0.90$) | | $\mathbf{1[ClO_4]_2}$·MeCN | | | |
|---|---|---|---|---|---|---|---|---|
| $T$ / K | 120 | 120 | 120 | 250 | 120 | | 250 | |
| | | | | | Molecule A | Molecule B | Molecule A | Molecule B |
| Fe(1)–N(2) | 1.909(4) | 1.891(3) | 1.894(3) | 1.925(4) | 1.898(3) | 1.899(3) | 2.119(4) | 2.072(4) |
| Fe(1)–N(9) | 1.993(4) | 1.977(3) | 1.984(3) | 2.006(4) | 1.977(2) | 1.974(3) | 2.172(4) | 2.146(4) |
| Fe(1)–N(14) | 1.987(4) | 1.980(3) | 1.987(3) | 1.998(4) | 1.977(3) | 1.979(2) | 2.165(4) | 2.151(4) |
| Fe(1)–N(23) | 1.906(4) | 1.892(3) | 1.897(3) | 1.937(3) | 1.897(3) | 1.901(2) | 2.102(3) | 2.092(4) |
| Fe(1)–N(30) | 1.982(4) | 1.969(3) | 1.965(3) | 1.993(4) | 1.966(2) | 1.959(2) | 2.192(4) | 2.143(4) |
| Fe(1)–N(35) | 1.982(4) | 1.972(3) | 1.979(3) | 2.004(4) | 1.990(2) | 1.988(2) | 2.189(4) | 2.138(5) |
| | | | | | | | | |
| N(2)–Fe(1)–N(9) | 79.87(15) | 80.18(11) | 80.16(11) | 79.04(14) | 79.51(10) | 79.82(10) | 73.12(14) | 74.60(15) |
| N(2)–Fe(1)–N(14) | 79.38(15) | 79.71(11) | 79.44(11) | 78.75(15) | 80.27(10) | 79.92(10) | 73.45(14) | 74.57(14) |
| N(2)–Fe(1)–N(23) | 175.72(15) | 177.28(12) | 176.56(11) | 175.45(14) | 175.36(10) | 174.87(11) | 174.65(13) | 168.83(15) |
| N(2)–Fe(1)–N(30) | 97.08(15) | 97.71(11) | 97.36(11) | 97.42(14) | 95.33(10) | 95.46(10) | 101.12(14) | 94.89(15) |
| N(2)–Fe(1)–N(35) | 103.50(15) | 102.17(11) | 102.90(11) | 104.74(14) | 105.01(10) | 104.80(10) | 111.49(13) | 117.13(16) |
| N(9)–Fe(1)–N(14) | 159.24(15) | 159.82(11) | 159.59(11) | 157.77(15) | 159.63(10) | 159.74(10) | 146.15(15) | 149.04(15) |
| N(9)–Fe(1)–N(23) | 97.27(15) | 98.04(12) | 97.72(11) | 98.13(14) | 100.05(10) | 102.55(10) | 106.74(14) | 105.99(15) |
| N(9)–Fe(1)–N(30) | 89.99(16) | 90.11(11) | 89.86(11) | 90.03(15) | 92.89(10) | 90.34(10) | 99.53(15) | 90.84(18) |
| N(9)–Fe(1)–N(35) | 94.42(16) | 93.61(11) | 94.29(11) | 94.38(16) | 89.99(10) | 92.07(10) | 88.93(16) | 94.29(17) |
| N(14)–Fe(1)–N(23) | 103.48(16) | 102.12(11) | 102.68(12) | 104.09(15) | 100.30(11) | 97.69(10) | 107.04(15) | 104.79(14) |
| N(14)–Fe(1)–N(30) | 93.33(16) | 94.15(11) | 93.27(12) | 93.70(16) | 91.36(10) | 92.22(10) | 91.86(15) | 94.48(15) |
| N(14)–Fe(1)–N(35) | 89.65(17) | 89.07(12) | 89.73(12) | 90.38(17) | 92.91(10) | 92.46(10) | 98.46(15) | 97.20(16) |
| N(23)–Fe(1)–N(30) | 79.68(15) | 80.19(11) | 79.88(11) | 78.95(14) | 80.07(10) | 80.04(10) | 73.57(13) | 73.98(14) |
| N(23)–Fe(1)–N(35) | 79.81(15) | 79.95(11) | 79.90(11) | 78.93(14) | 79.58(10) | 79.77(10) | 73.80(13) | 74.04(15) |
| N(30)–Fe(1)–N(35) | 159.41(15) | 160.11(11) | 159.73(11) | 157.84(15) | 159.64(11) | 159.71(10) | 147.36(14) | 147.79(16) |
| | | | | | | | | |
| $V_{Oh}$ | 9.690(12) | 9.520(8) | 9.571(9) | 9.897(11) | 9.565(8) | 9.554(8) | 12.206(14) | 11.715(14) |
| $\Sigma$ | 90.7(5) | 88.8(4) | 89.2(4) | 97.2(5) | 88.4(3) | 88.0(3) | 153.4(5) | 142.4(5) |
| $\Theta$ | 296 | 286 | 291 | 320 | 292 | 290 | 497 | 470 |
| $\phi$ | 175.72(15) | 177.28(12) | 176.56(11) | 175.45(14) | 175.36(10) | 174.87(11) | 174.65(13) | 168.83(15) |
| $\theta$ | 85.12(4) | 84.10(3) | 85.32(3) | 85.04(4) | 88.71(3) | 89.62(2) | 85.33(5) | 88.52(5) |

**Table S12** Hydrogen bond parameters for **1[BF$_4$]$_2$**·$H_2O$ (Å , °). See Figure S27 for the atom numbering scheme.

| | D−H | H···A | D···A | D−H···A |
|---|---|---|---|---|
| O(54)−H(54A)···F(47) | 0.923(19) | 2.02(2) | 2.919(4) | 163(5) |
| O(54)−H(54A)···F(50A)/F(50B)/F(51C) | 0.911(19) | 1.86(2)/2.06(3)/1.97(2) | 2.757(9)/2.910(14)/2.838(8) | 168(5)/154(5)/159(5) |

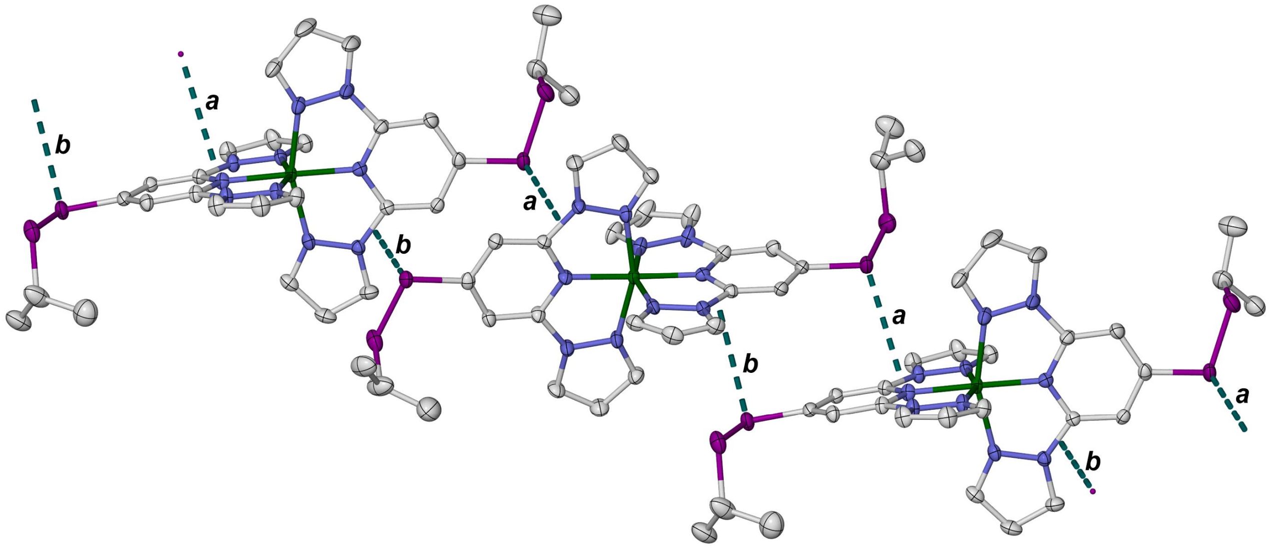


**Figure S30** Intermolecular $n\cdots\pi$ contacts in **1[ClO$_4$]$_2$**·*n*MeNO$_2$ at 120 K, showing the association of the cations into 1D chains. Color code: C, white; Cl, yellow; Fe, green; N, blue; O, red; S, purple.

The $\alpha$-S atom from each *iso*propyldisulfanyl substituent overlies the centroid of a C−N bond, linking pyridyl and pyrazolyl heterocycle in its neighbor molecule. The distances are:

***a***: S(18)···[C(24$^{vii}$)−N(29$^{vii}$)] – symmetry code (vii) −½+*x*, ½−*y*, −½+z]

***b***: S(39)···[C(3$^{viii}$)−N(8$^{viii}$)] – symmetry code (viii) ½+*x*, ½−*y*, ½+*z*]

The distances of the weak interactions for the structures exhibiting this mode of crystal packing are listed in the following Table.

**Table S13** Intermolecular $n\cdots\pi$ contacts in crystal structures isomorphous with Figure S30.

| Distance | ***a*** | ***b*** |
|---|---|---|
| **1[BF$_4$]$_2$**·H$_2$O, 120 K | 3.357 | 3.536 |
| **1[BF$_4$]$_2$**·MeNO$_2$, 120 K | 3.387 | 3.580 |
| **1[ClO$_4$]$_2$**·*n*MeNO$_2$, 120 K | 3.382 | 3.600 |
| **1[ClO$_4$]$_2$**·*n*MeNO$_2$, 250 K | 3.436 | 3.564/3.641[a] |

[a]This S atom of this *iso*propyldisulfanyl group is disordered at this temperature.

The sum of the Pauling van der Waals radii of an S atom and an aromatic ring is 3.55 Å.[18] Hence, contact ***a*** corresponds to a significant attractive intermolecular interaction in all these structures, but ***b*** is longer and probably just corresponds to a van der Waals contact.

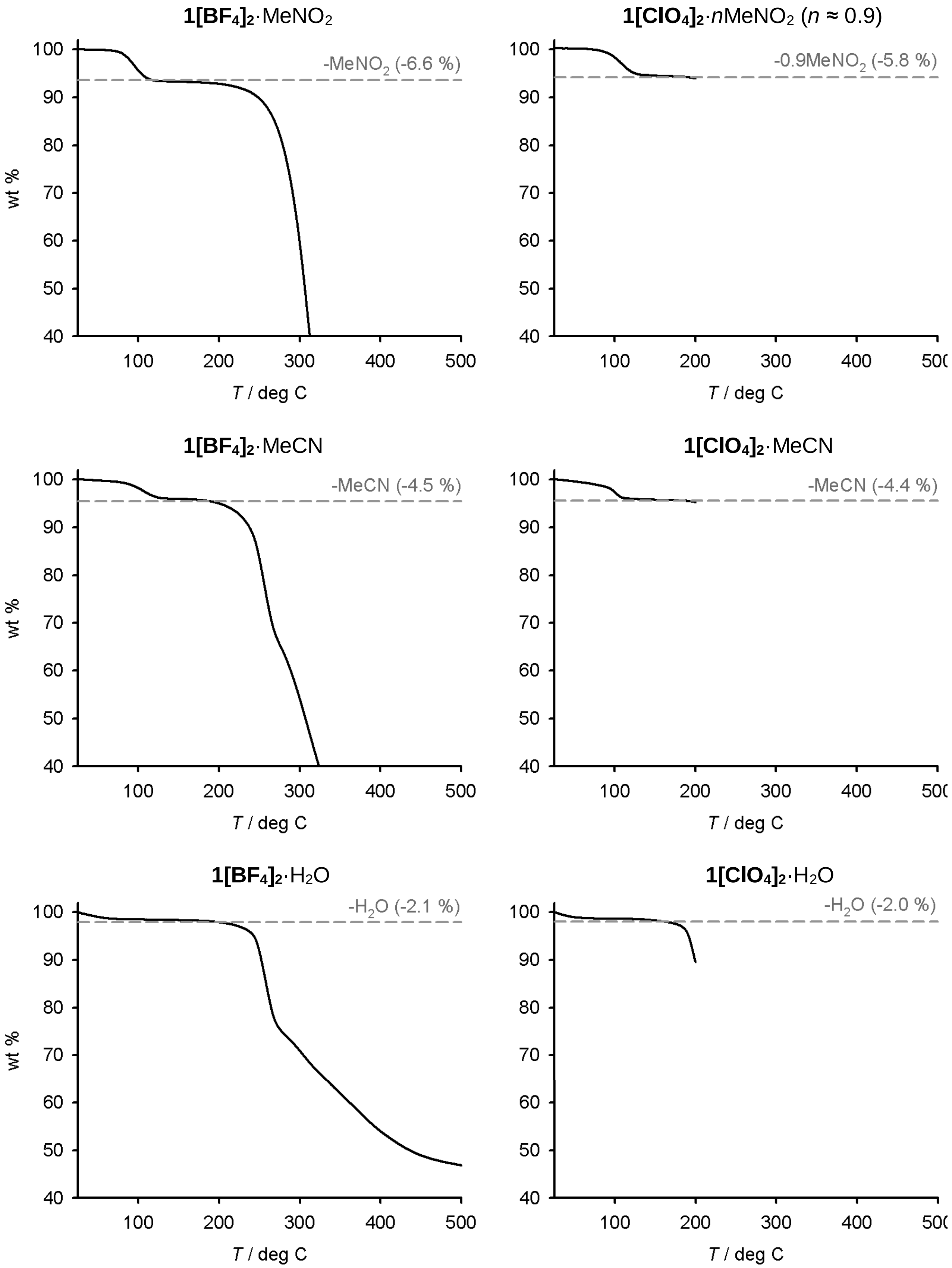


**Figure S31** Thermogravimetric analyses for the nitromethane, acetonitrile solvate and hydrate $\mathbf{1X_2}$ materials. The perchlorate salts were only heated to 200 °C, because of their risk of detonation at higher temperatures.

The measured mass loss for $\mathbf{1[ClO_4]_2}\cdot n$MeNO$_2$ is a good fit for its crystallographic composition with $n = 0.9$ (a whole equivalent of MeNO$_2$ would give a mass loss of 6.4 %). The hydrate materials lose their water easily. However, like all the dried solvates, they also reabsorb that moisture quickly when exposed to air.

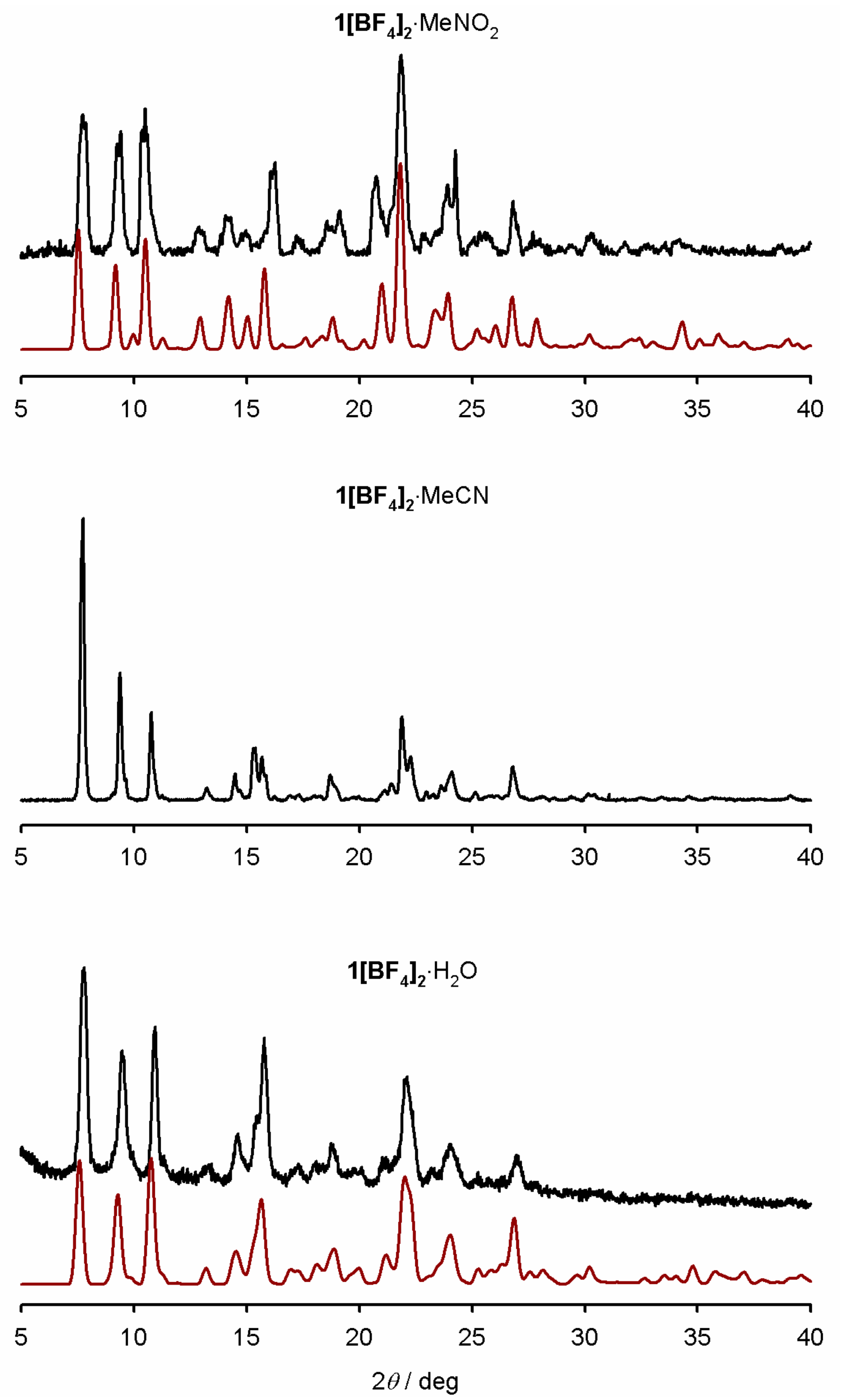


**Figure S32** Measured (black) and simulated (red) X-ray powder diffraction patterns for the other solvates of **1[BF$_4$]$_2$**.

**1[BF$_4$]$_2$**·MeNO$_2$ and **1[BF$_4$]$_2$**·H$_2$O are isomorphous crystallographically, and their powder patterns are a good match for simulation. No crystal structure is available for **1[BF$_4$]$_2$**·MeCN (its crystals were consistently twinned), but its powder pattern looks isomorphous with these other sovates.

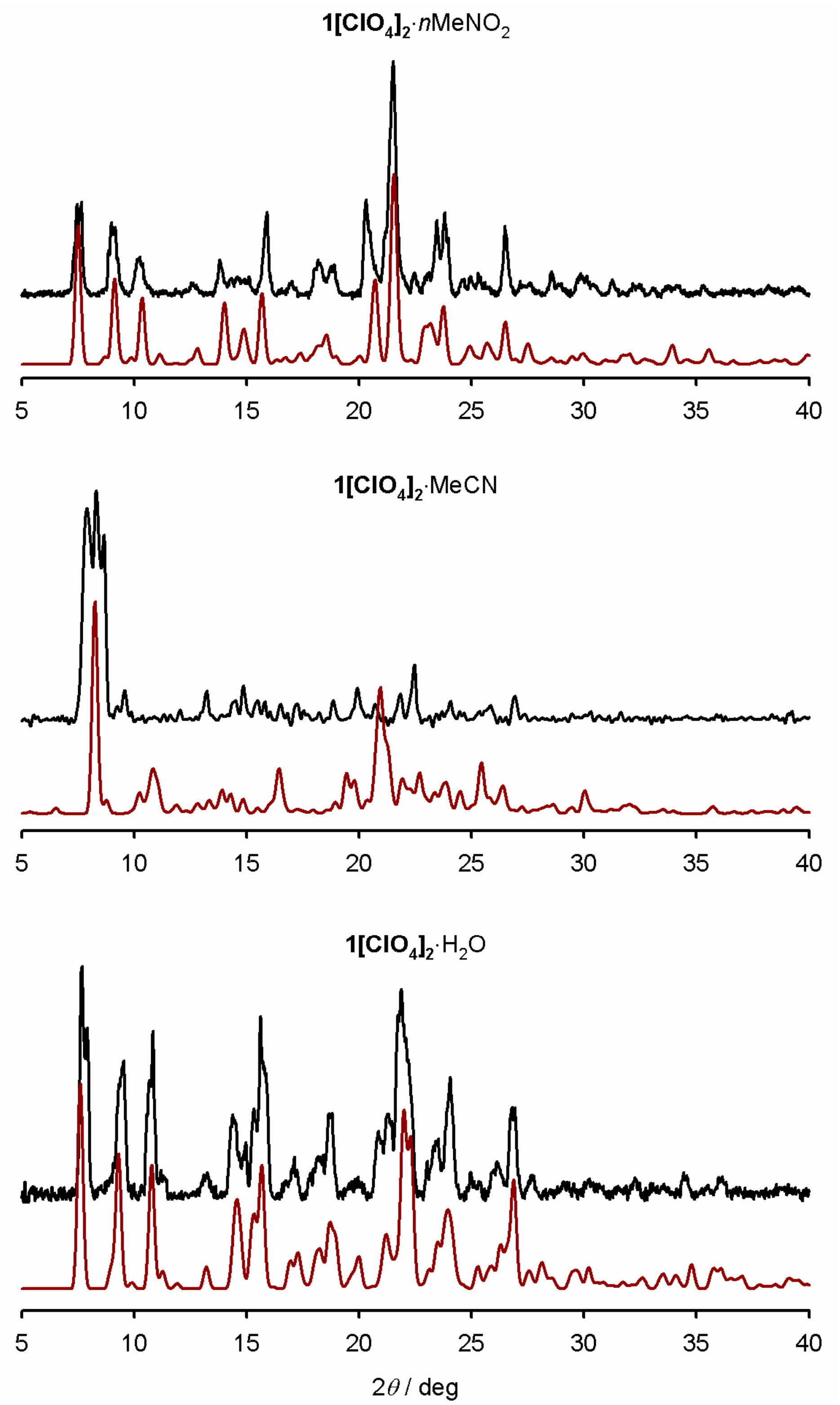


**Figure S33** Measured (black) and simulated (red) X-ray powder diffraction patterns for the other solvates of **1[ClO$_4$]$_2$**. The simulation of **1[ClO$_4$]$_2$**·H$_2$O is derived from the crystal structure of **1[BF$_4$]$_2$**·H$_2$O, with the BF$_4^-$ ions replaced by ClO$_4^-$.

The powder patterns of **1[ClO$_4$]$_2$**·*n*MeNO$_2$ and **1[ClO$_4$]$_2$**·H$_2$O are isomorphous with each other, and with the corresponding BF$_4^-$ solvate salts (Figure S32). However, the powder pattern of **1[ClO$_4$]$_2$**·MeCN does not match its single crystal phase, or the other solvates in this work. It may have undergone a phase change following solvent loss during sample preparation; or, the single crystal phase may simply be a minor contaminent of the bulk sample, which adopts another structure type. In any case, the crystal structures of **1[ClO$_4$]$_2$**·MeCN clearly do not correspond to the structure of that bulk material.

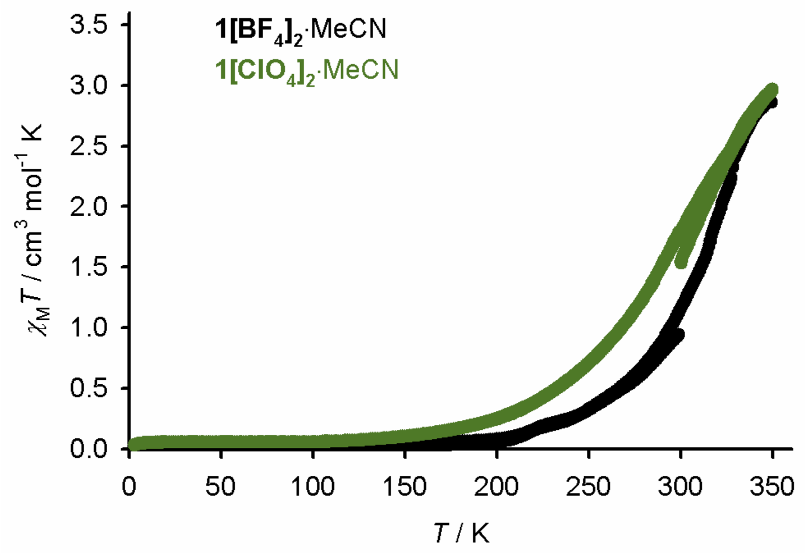


**Figure S34** Variable temperature magnetic data for the acetonitrile solvate materials. Data were collected on a 300⟶350⟶5⟶300 K temperature cycle, at a scan rate of 5 K $min^{-1}$.

Data for the nitromethane solvate and hydrate compounds, which are isomorphous with **1[BF$_4$]$_2$**·MeCN, are in Figure 8 of the main article.

All the **1X$_2$**·MeCN, **1X$_2$**·MeCN and **1X$_2$**·$H_2O$ compounds are isomorphous by powder diffraction, except for **1[ClO$_4$]$_2$**·MeCN (Figures S31-S32). The data for all these compounds show good reversibility after warming to 350 K, implying they are not affected by *in situ* solvent loss during the measurement. That contrasts with the acetone solvates, which are more sensitive to solvent loss under the same conditions.

**Discussion of the Hirshfeld surface analyses**

A Hirshfeld surface is the boundary surrounding a molecule in a crystal, where the electron density from the enclosed molecule is equal to that from its nearest neighbors.[20] The surface can be plotted in various ways, including interaction (or fingerprint) maps which show intermolecular distances from each atom inside the surface ($d_i$, i = internal) and its nearest neighbours in the lattice ($d_e$, e = external). These are scaled according to their distance from the Hirshfeld surface about the residue of interest.[21] Intermolecular contacts between different elements are plotted separately, chosen to highlight relevant C–H...X (X = O or F), C–H...$\pi$, anion...$\pi$, or O–H...X hydrogen bonding intermolecular interactions.[22]

Each graph is marked with the Pauling Van der Waals radii of the elements plotted.[18] Only data points with $d_i$ and $d_e$ less than the relevant Van der Waals radius are significant intermolecular contacts. Strong interactions like O–H...X hydrogen bonds afford characteristic sharp lines on the donor X...H and acceptor X...H maps, projecting well below the Van der Waals radii of each element. Weaker interactions like C–H...X or anion...$\pi$ appear broader in the maps, and extend only slightly below the Van der Waals radii limits.

A limitation is that the technique does not account for disorder. When one of the two interacting molecules is disordered, it leads to a broadening in the fingerprint map of the interaction, which may also appear artificially shortened. Such disorder is highlighted on the following Figures, where relevant.

Interaction maps are plotted for the isothermal high-spin and low-spin structures in **1[BF$_4$]$_2$**·sf and **1[ClO$_4$]$_2$**·sf, and for the other available crystal structures in this work. Three maps are plotted for each compound, which respectively highlight weak intermolecular C–H···Y (Y = F or O) contacts between the cations and anion in the lattice; weak anion···$\pi$ interactions; and, the pairwise $n$···$\pi$ contacts between cations involving their disulfanyl groups.

Data for two compounds allow the influence of temperature on these maps to be probed. Firstly, isothermal low- and high-spin data for **1[BF$_4$]$_2$**·sf are plotted at 100 K, together with the 250 K high-spin structure. Secondly, the low- and high-spin structures of **1[ClO$_4$]$_2$**·sf are both available at two temperatures, including isothermal analyses at 170 K.

While disorder at the higher temperatures complicates the comparison, the Hirshfeld fingerprints near the van der Waals radii for each spin state at different temperatures are essentially the same (Figures S36-S37). This is especially clear for **1[ClO$_4$]$_2$**·sf, where the two high-spin structures exhibit essentially the same disorder regime (Figure S37). Evidently, SCO has a greater effect on the Hirshfeld interaction fingerprints in these compounds, than the background contraction or expansion on the lattice between temperatures.[23] That is consistent with another of our recent studies, which drew the same conclusion.[24]

More detailed interpretations of the intermolecular interactions in each structure are given beside the relevant Figure. The most important conclusion is that there are short, directional intermolecular interactions in **1X$_2$**·Me$_2$CO and **1X$_2$**·sf that can explain their hysteretic SCO, in isolation. Rather, we attribute this SCO cooperativity to two factors:

(i) The large angular structural rearrangements undergone by the cations during SCO, and;

(ii) The large surface contact area between neighbor cations, which is a consequence of the pairwise $n$···$\pi$ interactions linking them into chains. That will efficiently transmit the molecular structure changes through the lattice as the transition proceeds.

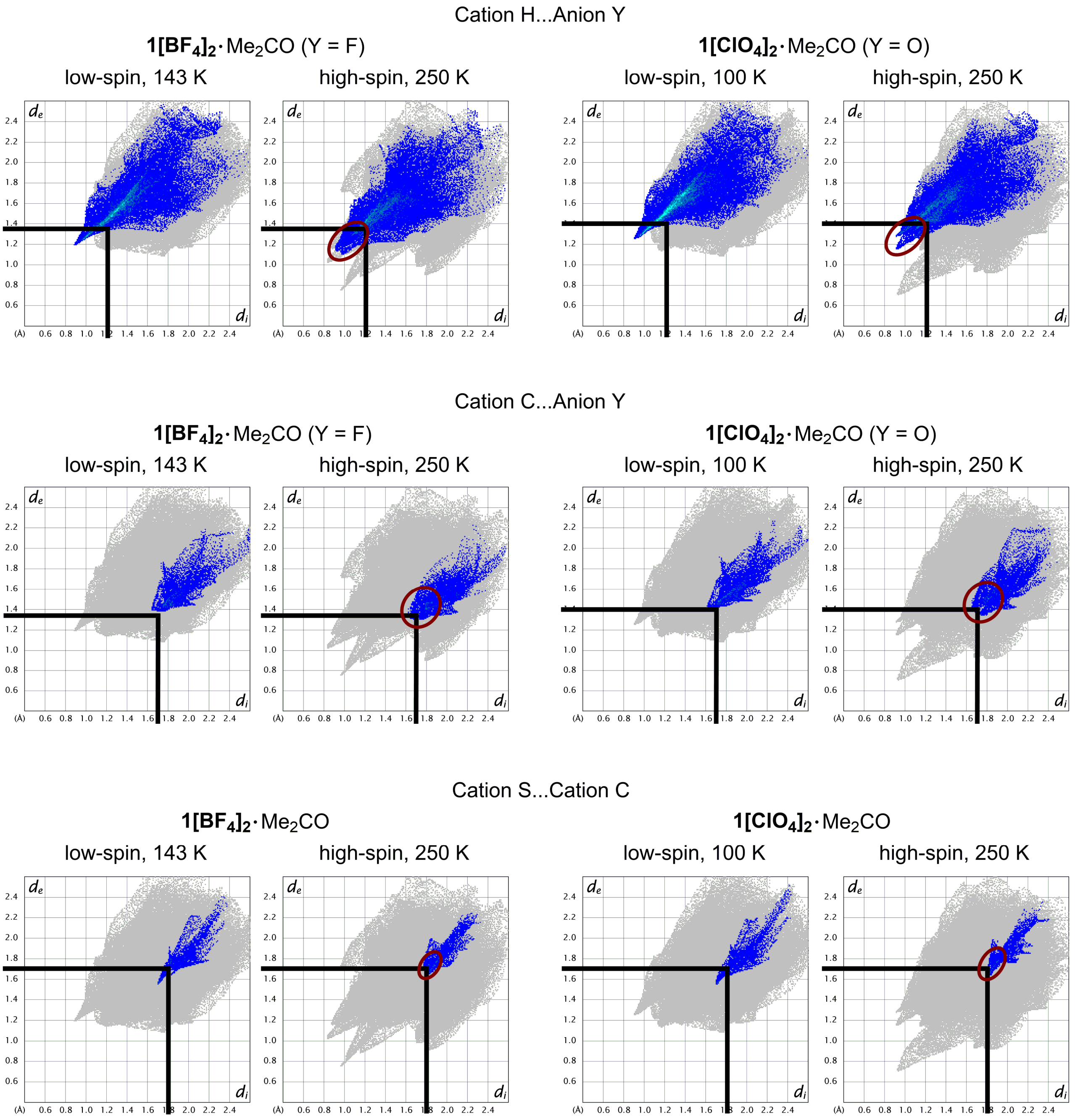


**Figure S35** Hirshfeld fingerprint maps of the two spin states of **1[BF$_4$]$_2$**·Me$_2$CO and **1[ClO$_4$]$_2$**·Me$_2$CO, showing intermolecular contacts surrounding the complex cation. See page S58 for more details.

The circled data points for the high-spin structures involve contacts to disordered $ClO_4^-$ ions or disulfanyl groups (Figures S4-S5), which should be treated with caution.

The plots show short, directional C–H···Y (Y = F or O) interactions in the lattice, which extend significantly below the covalent radii of an H and F/O atom. Two contacts contribute to this:

- C(33)–H(33)···F(46$^{ix}$) [symmetry code (ix): *x*, $^3$/2–*y*, –½+*z*], where H(33)···F(46$^{ix}$) = 2.26 Å for low-spin **1[BF$_4$]$_2$**·Me$_2$CO. This contact is longer at H(33)···O(46$^{ix}$) = 2.34 Å in **1[ClO$_4$]$_2$**·Me$_2$CO at 110 K.
- C(38)–H(38)···Y(45$^{x}$) [symmetry code (x): –1+*x*, $^3$/2–*y*, –½+*z*], where H(38)···F(45$^{x}$) = 2.21 Å for **1[BF$_4$]$_2$**·Me$_2$CO and H(38)···O(45$^{x}$) = 2.28 Å for **1[ClO$_4$]$_2$**·Me$_2$CO in their low-spin crystal structures.

The pairwise intermolecular $n\cdots\pi$ interactions in Figure S30 are also clearly visible in the low-spin S···C fingerprint maps. These contacts are up to 0.18 Å shorter in the low-spin structures, while the high-spin S···C maps are also broadened by disorder in some S atoms.

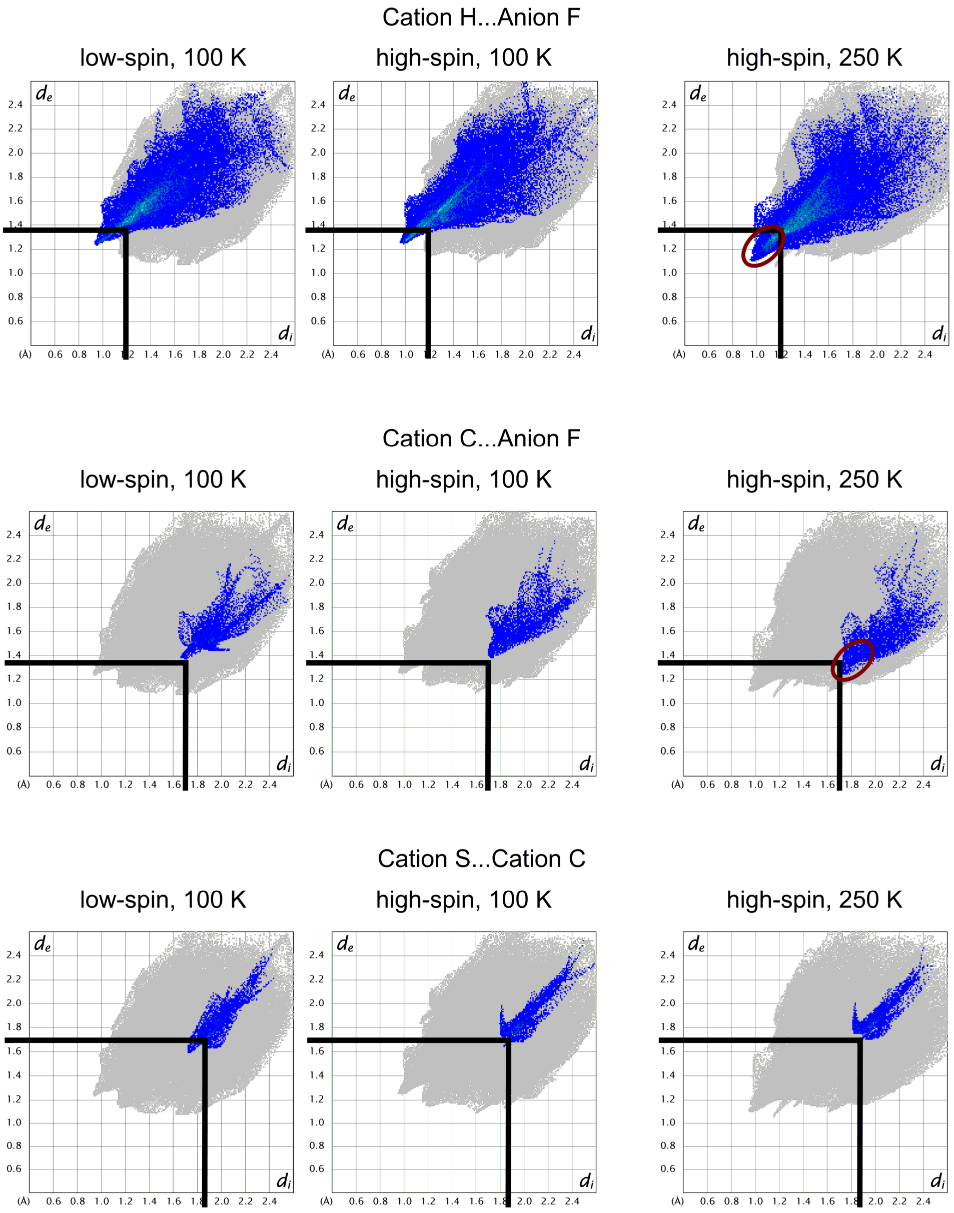


**Figure S36** Hirshfeld fingerprint maps of **1[BF$_4$]$_2$**·sf in its isothermal high- and low-spin states, showing intermolecular contacts surrounding the complex cation. Data from a 250 K high-spin structure are also included for comparison. See page S58 for more details.

The circled data points for the 250 K structure involve contacts to disordered $BF_4^-$ ions (Figure S14), which should be treated with caution.

These data are discussed on the following page.

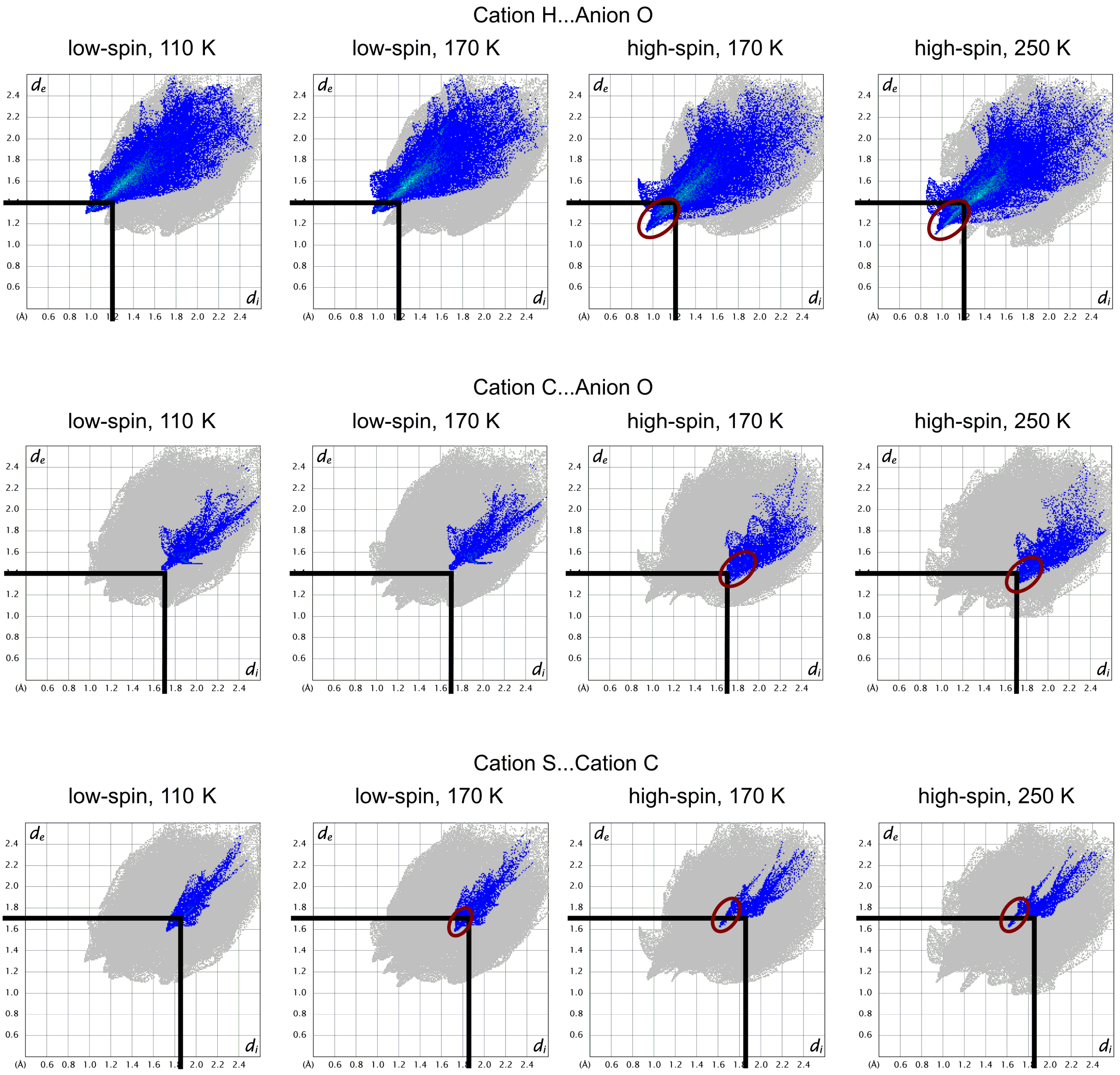


**Figure S37** Hirshfeld fingerprint maps of **1[ClO$_4$]$_2$**·sf in its isothermal high- and low-spin states, showing intermolecular contacts surrounding the complex cation. Data from the 110 K low-spin and 250 K high-spin structures are also included for comparison. See page S58 for more details.

The circled data points for the structures at $T$ > 110 K involve contacts to disordered $ClO_4^-$ ions or disulfanyl groups (Figure S15), which should be treated with caution.

Intermolecular C–H···Y (Y = F or O) interactions are less pronounced in the fingerprint maps for **1[BF$_4$]$_2$**·sf and **1[ClO$_4$]$_2$**·sf, than for the acetone solvates. Consistent with that, there are no intermolecular H···Y distances of <2.35 Å in those lattices.

The long, thin tail on the low-spin C···Y interaction maps implies a weak but directional anion···$\pi$ contact, which is not evident for the acetone solvate crystals. One anion 'Y' atom, F(46)/O(46), is indeed oriented towards the centroid of a pyridyl ring in **1[BF$_4$]$_2$**·sf and **1[ClO$_4$]$_2$**·sf. The Y···centroid distances of 2.9-3.0 Å imply this is simply a Van der Waals contact, however, which is why the interaction does not extend below the covalent radii in the plots.

The pairwise $n$···$\pi$ interactions in Figure S16 are less clear in these plots. The closest intermolecular S···C contacts in **1[BF$_4$]$_2$**·sf and **1[ClO$_4$]$_2$**·sf are *ca* 0.2 Å longer than in the acetone solvates, when those S atoms are crystallographically ordered (Table S8). That should not impact the ability of these contacts to mediate mechanical coupling between the cations during the cooperative spin-transitions, however.

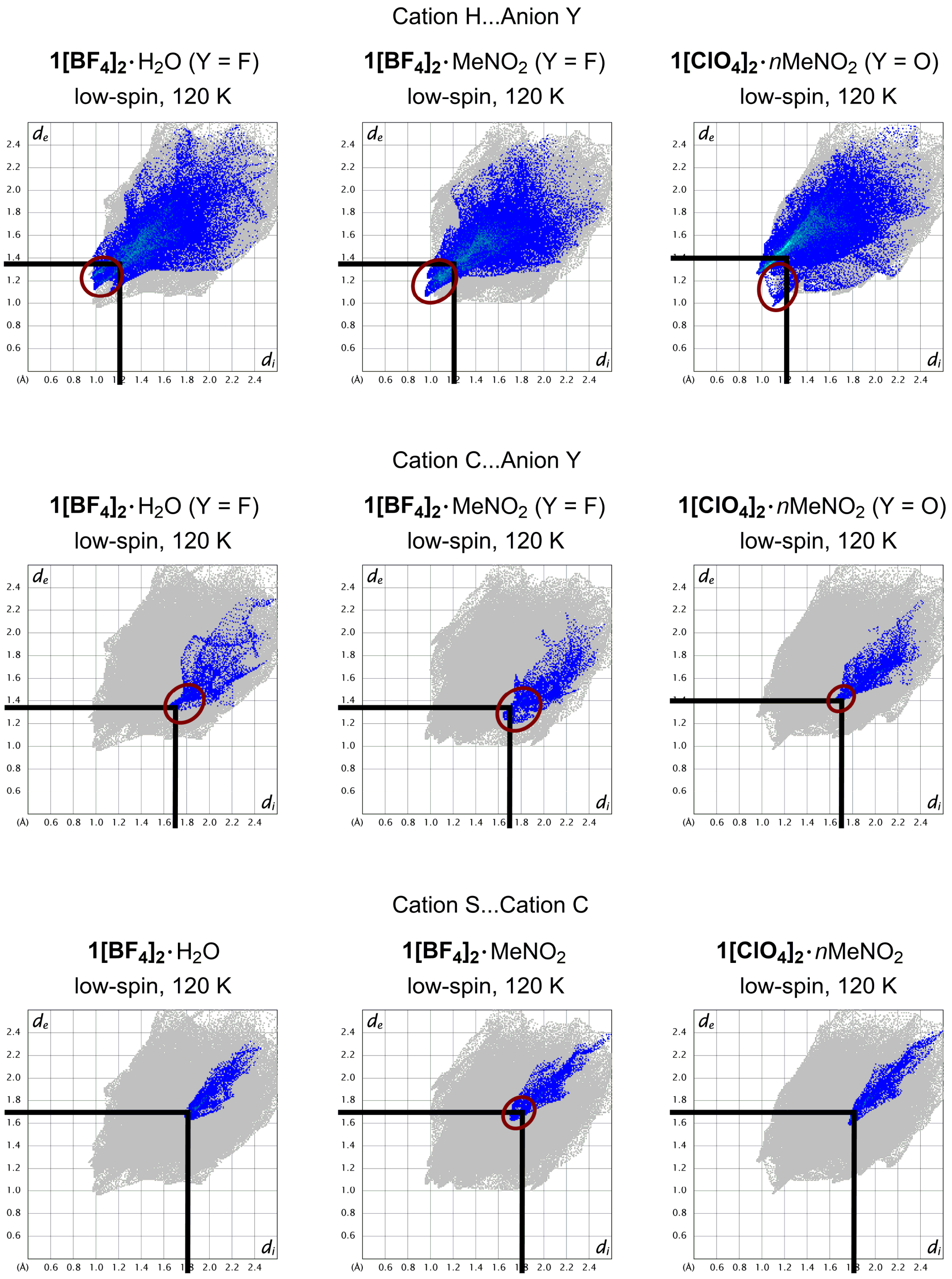


**Figure S38** Hirshfeld fingerprint maps for the low temperature crystal structures of the other, isomorphous solvates of **1[BF$_4$]$_2$** and **1[ClO$_4$]$_2$** exhibiting gradual thermal SCO. See page S58 for more details.

The circled data points involve contacts to disordered anions or disulfanyl groups (Figures S26-S28), which should be treated with caution.

There are no notably short, directional intermolecular contacts, which are not contaminated by disorder, in any of these fingerprint maps.

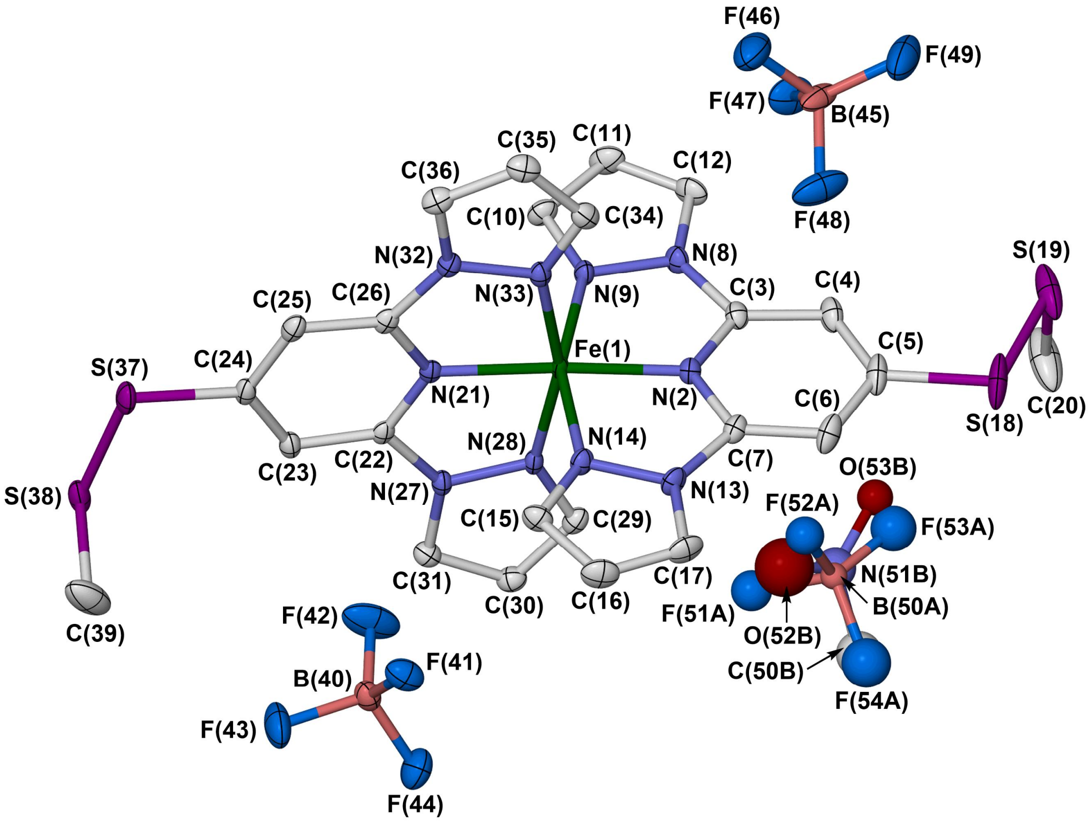


**Figure S39** The asymmetric unit of **2[BF$_4$]$_2$**·0.5MeNO$_2$ at 100 K, with the full atom numbering scheme. Displacement ellipsoids are at the 50 % probability level, and H atoms are omitted for clarity. Color code: C, white; B, pink; F, cyan; Fe, green; N, blue; O, red; S, purple.

The same atom numbering is used for all the **2[BF$_4$]$_2$** structures in this study. Atoms B(50A)-F(54A) and C(50B)-O(53B) comprise a region of the lattice containing an overlaid 1:1 mixture of $BF_4^-$ ion and nitromethane solvent. See Figure S39 for more details.

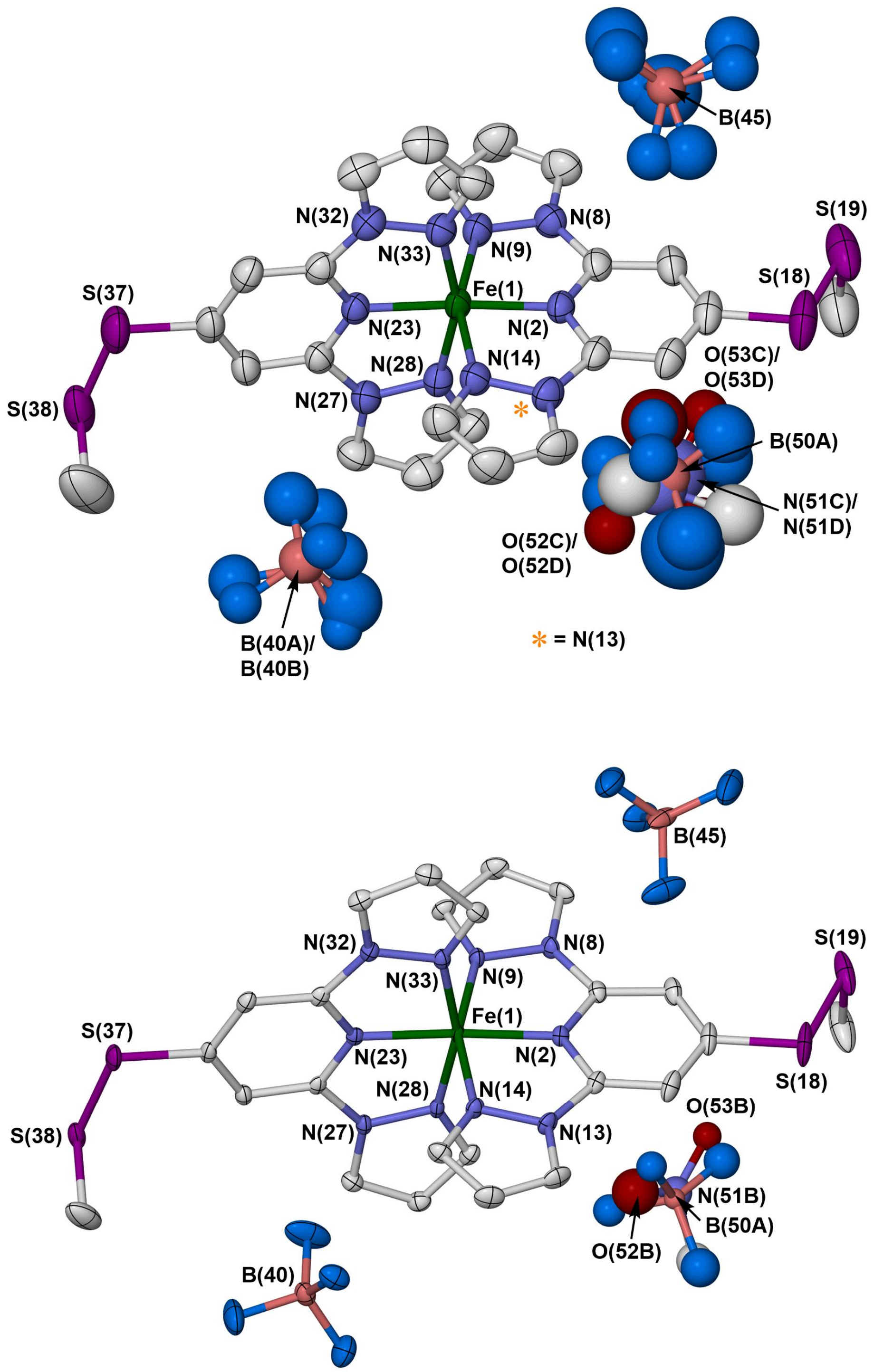


**Figure S40** The asymmetric unit of **2[BF$_4$]$_2$**·0.5MeNO$_2$. Top, at 290 K; bottom, at 100 K. The crystal is low-spin at both temperatures. Other details as for Figure S39, which also shows the full atom numbering scheme for the structure. Color code: C, white; B, pink; F, cyan; Fe, green; N, blue; O, red; S, purple.

Anion B(40)-F(44) is fully occupied on a general crystallographic site, but B(45)-F(49) lies near the crystallographic inversion center, and is only half-occupied by symmetry. The remaining half-anion equivalent B(50A)-F(54A), is superimposed upon a half-solvent molecule C(50B)-O(53B) occupying the same lattice site. The disordered region occupies a cavity spanning the inversion center 1, 0, 0, with each cavity containing one anion and one solvent molecule in a disordered distribution about that inversion center.

The composition of the mixed anion/solvent site is obvious in the 100 K structure, where each individual residue is crystallographically ordered. The disorder at 290 K makes this less clear, but the refinement is consistent with the 100 K model and is crystallographically reasonable.

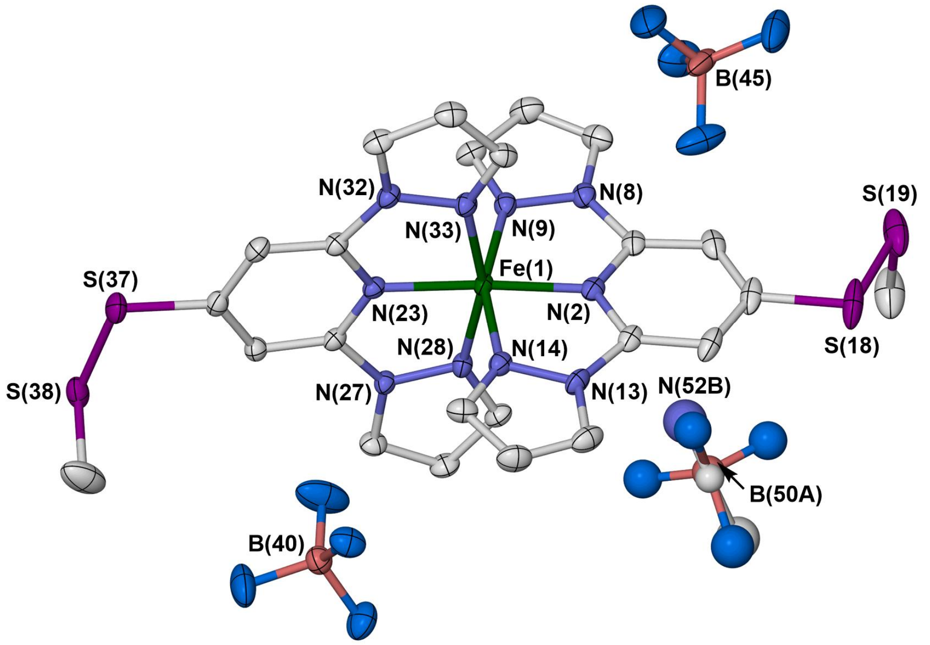


**Figure S41** The asymmetric unit of **2[BF$_4$]$_2$**·0.5MeCN at 120K. Other details as for Figure S39, which also shows the full atom numbering scheme for the structure. Color code: C, white; B, pink; F, cyan; Fe, green; N, blue; S, purple.

This crystal is isomorphous with **2[BF$_4$]$_2$**·0.5MeNO$_2$ in the previous Figure, with the half-anion equivalent B(50A)-F(54A) being superimposed upon a half-solvent molecule C(50B)-N(52B) in the same lattice site.

**Table S14** Selected bond lengths, angles and other structural parameters (Å , °, $Å^3$) for the solvates of **2[BF$_4$]$_2$**. See Figures S39-S41 for the atom numbering scheme, while definitions of $V_{Oh}$, $\Sigma$, $\Theta$, $\varphi$ and $\theta$ are given on page S16.

| | **2[BF$_4$]$_2$**·0.5MeNO$_2$ | | **2[BF$_4$]$_2$**·0.5MeCN |
|---|---|---|---|
| $T$ / K | 100 | 290 | 120 |
| Fe(1)–N(2) | 1.895(2) | 1.902(3) | 1.893(2) |
| Fe(1)–N(9) | 1.975(2) | 1.983(4) | 1.981(2) |
| Fe(1)–N(14) | 1.956(2) | 1.962(4) | 1.955(2) |
| Fe(1)–N(21) | 1.893(2) | 1.898(3) | 1.893(2) |
| Fe(1)–N(28) | 1.964(2) | 1.964(4) | 1.957(2) |
| Fe(1)–N(33) | 1.970(2) | 1.973(4) | 1.970(2) |
| | | | |
| N(2)–Fe(1)–N(9) | 79.95(8) | 79.65(15) | 79.92(9) |
| N(2)–Fe(1)–N(14) | 80.31(8) | 80.39(15) | 80.44(9) |
| N(2)–Fe(1)–N(21) | 175.78(8) | 175.71(16) | 175.64(9) |
| N(2)–Fe(1)–N(28) | 99.28(8) | 98.72(15) | 98.57(9) |
| N(2)–Fe(1)–N(33) | 100.25(8) | 101.20(15) | 101.06(9) |
| N(9)–Fe(1)–N(14) | 160.26(9) | 160.03(15) | 160.36(9) |
| N(9)–Fe(1)–N(21) | 104.24(8) | 104.41(15) | 104.27(9) |
| N(9)–Fe(1)–N(28) | 93.41(8) | 93.19(15) | 93.41(9) |
| N(9)–Fe(1)–N(33) | 91.78(8) | 91.70(16) | 91.86(9) |
| N(14)–Fe(1)–N(21) | 95.50(8) | 95.56(15) | 95.37(9) |
| N(14)–Fe(1)–N(28) | 90.06(8) | 90.43(15) | 89.93(9) |
| N(14)–Fe(1)–N(33) | 91.41(8) | 91.56(16) | 91.46(9) |
| N(21)–Fe(1)–N(28) | 80.12(8) | 79.80(15) | 80.12(8) |
| N(21)–Fe(1)–N(33) | 80.27(8) | 80.23(15) | 80.16(8) |
| N(28)–Fe(1)–N(33) | 160.38(9) | 160.03(15) | 160.28(9) |
| | | | |
| $V_{Oh}$ | 9.461(6) | 9.524(11) | 9.454(7) |
| $\Sigma$ | 85.3(3) | 86.7(5) | 85.4(3) |
| $\Theta$ | 281 | 286 | 281 |
| $\phi$ | 175.78(8) | 175.71(16) | 175.64(9) |
| $\theta$ | 87.52(2) | 87.53(4) | 87.32(2) |

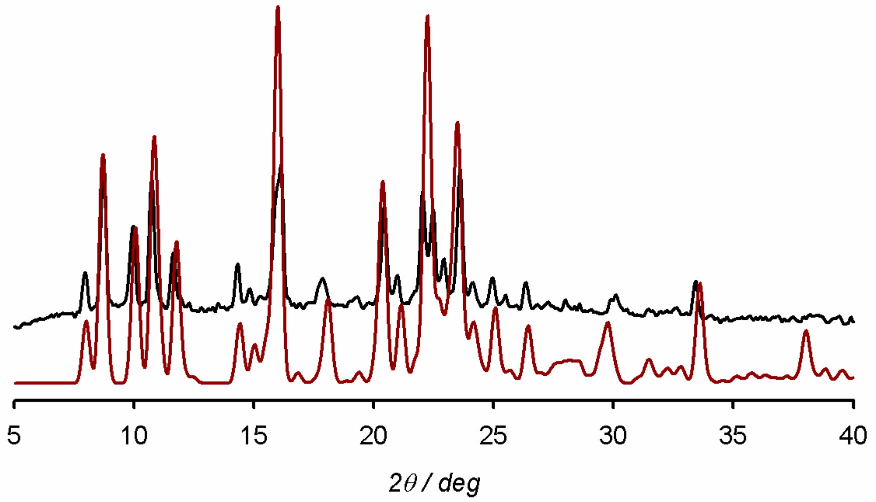


**Figure S42** Measured (black) and simulated (red) X-ray powder diffraction pattern for **2[BF$_4$]$_2$**·0.5MeNO$_2$.

The powder pattern is quite broad, but the agreement with the simulation is excellent.

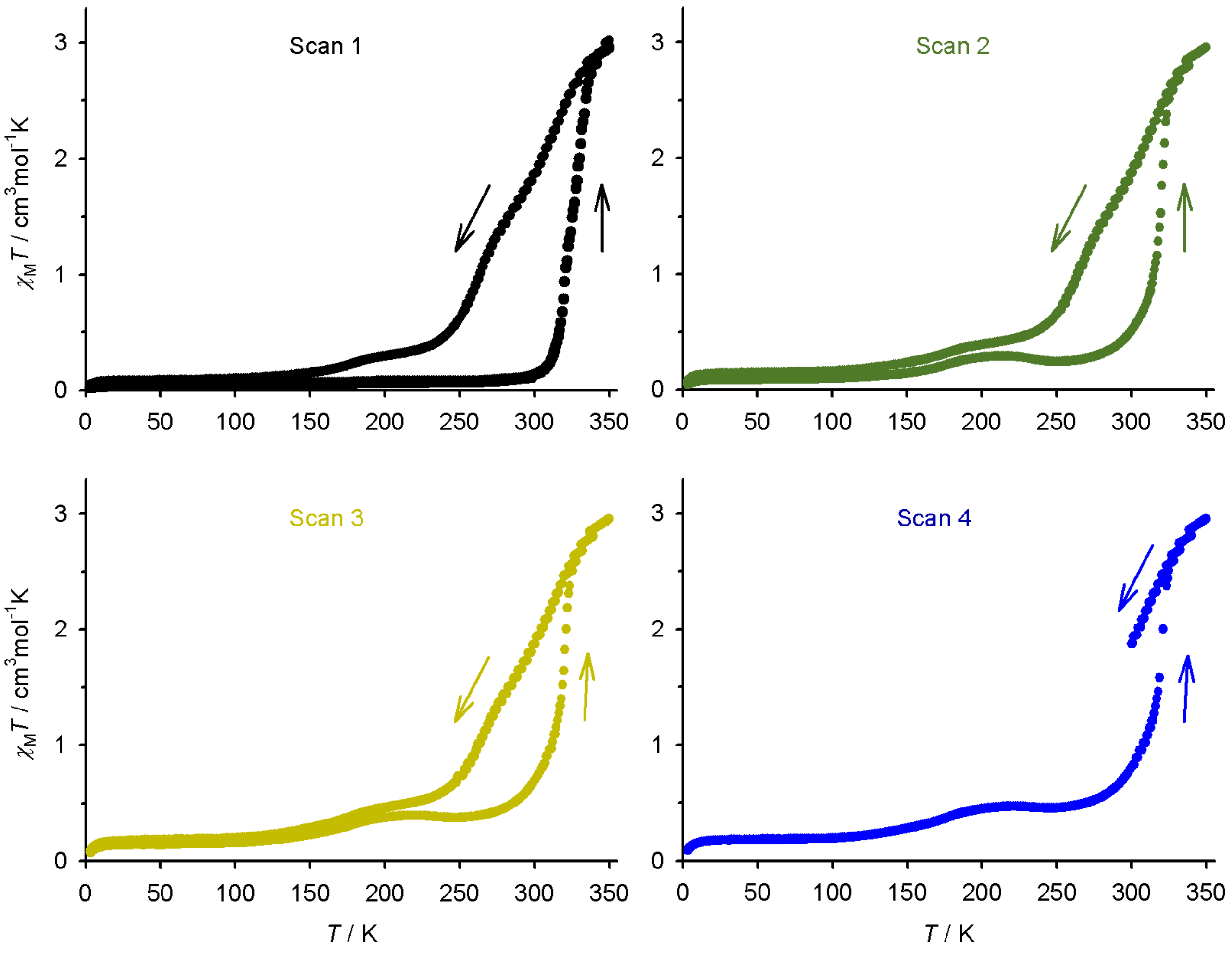


**Figure S43** Individual plots of the multiple scans of **2[BF$_4$]$_2$**·0.5MeNO$_2$ inside the SQUID magnetometer (Figure 9, main article). Four consecutive thermal scans are shown, all at scan rate 5 K min$^{-1}$:

(1) 300→3→350→3 K (black);
(2) 3→350→3 K (green);
(3) 3→350→3 K (yellow);
(4) 3→350→300 K (blue).

The feature near 200 K first appears on the cooling branch of scan 1, but is present in both warming and cooling modes for scans 2-4. It also slowly grows in on repeated scanning. We proposes this feature arises from slow desolvation of the sample as the experiment proceeds.

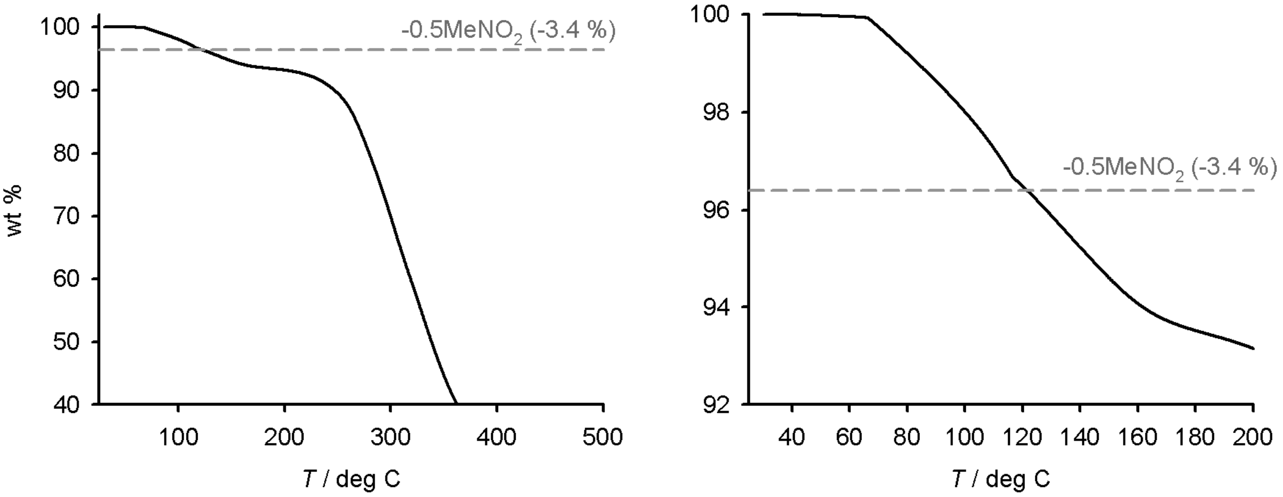


**Figure S44** Thermogravimetric analyses for **2[BF$_4$]$_2$**·0.5MeNO$_2$. Left: full scan. Right, expansion of the initial mass loss.

The curve plateaus near 160 °C at –6.0 % mass loss, which corresponds to *ca* 0.8 equiv MeNO$_2$. However, the expansion shows an inflection in the curve at 115 °C/3.3 % mass loss, close to the expected value for the crystallographic solvent content as shown in the Figure.

The origin of the additional mass loss is uncertain. There were no crystalline contaminents in the sample (Figure S42), and no significant void space in the crystal structure that could accommodate extra solvent. One possibility could be partial decomposition of the $BF_4^-$ ions, which is usually expected above 200 °C but can occur at lower temperatures in some circumstances.[25]

Be that as it may, the material is stable to solvent loss below 68 °C (340 K), and only loses solvent slowly above that temperature. That is consistent with our suggestion that the feature near 200 K in the magnetic measurements, which grows in slowly upon repeated thermal scanning, arises from slow solvent loss from the sample (Figure S43).

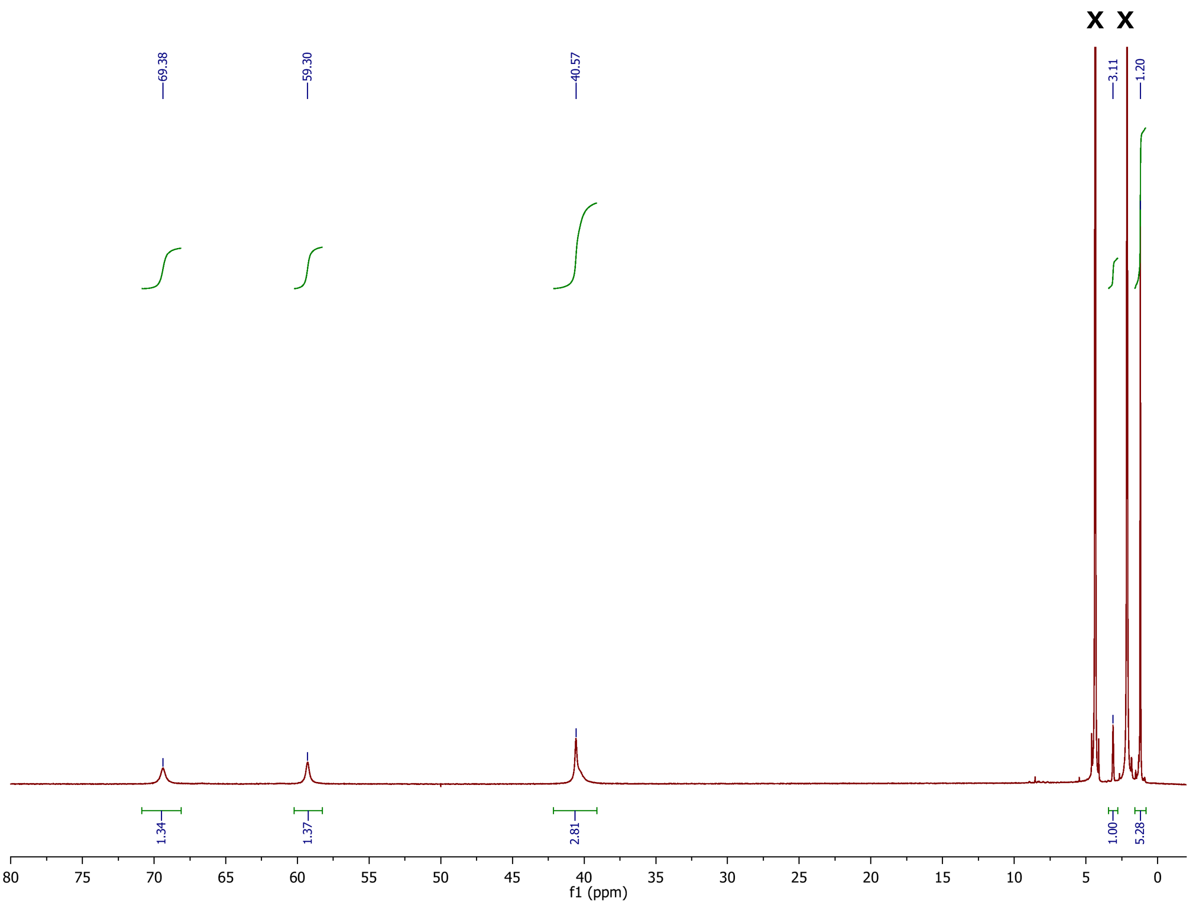


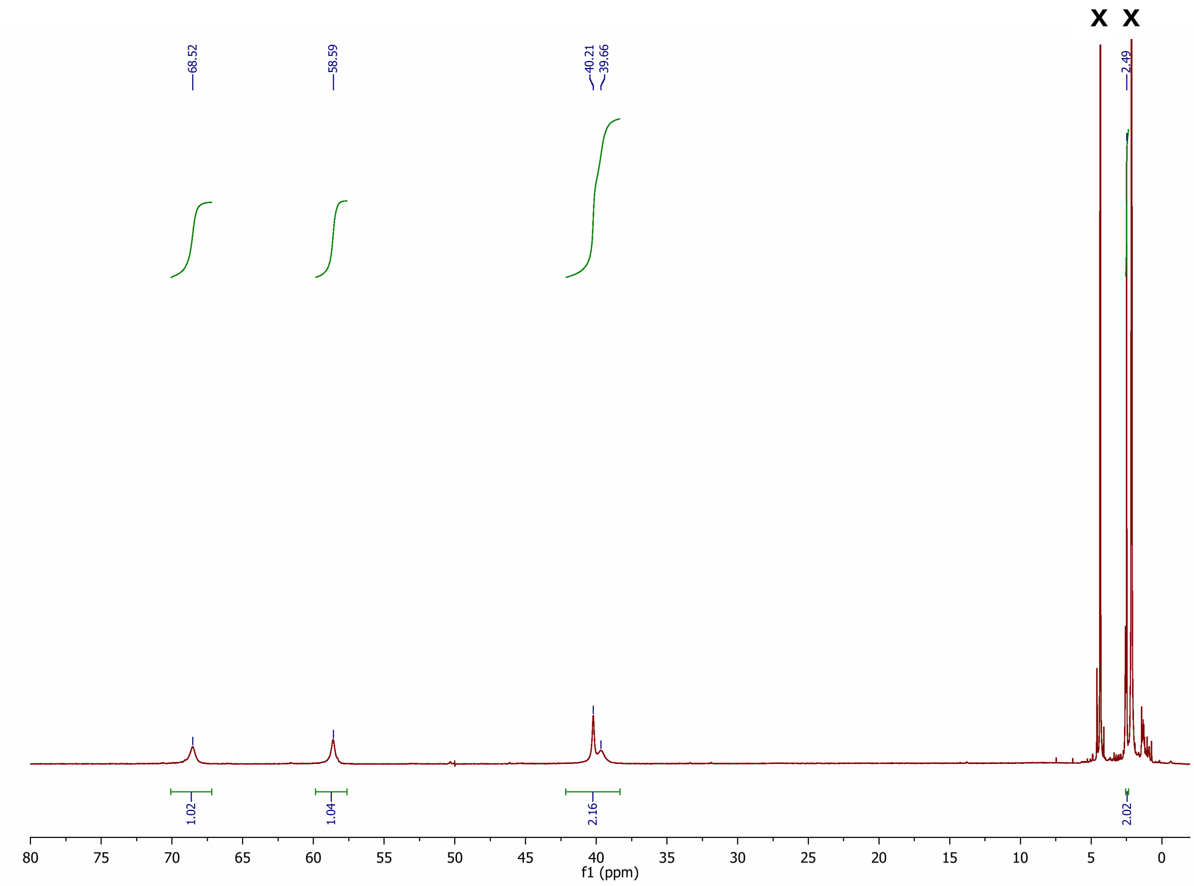


**Figure S45** $^{1}$H NMR spectra of **1[BF$_4$]$_2$** (top) and **2[BF$_4$]$_2$** (bottom) in $CD_3NO_2$.

**1[BF$_4$]$_2$**

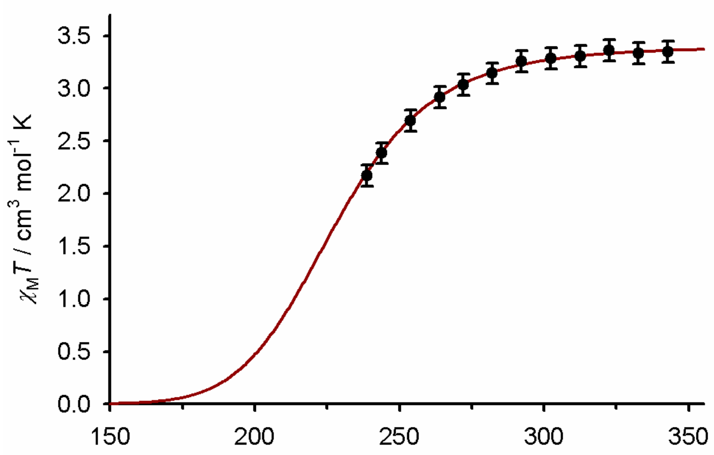


**2[BF$_4$]$_2$**

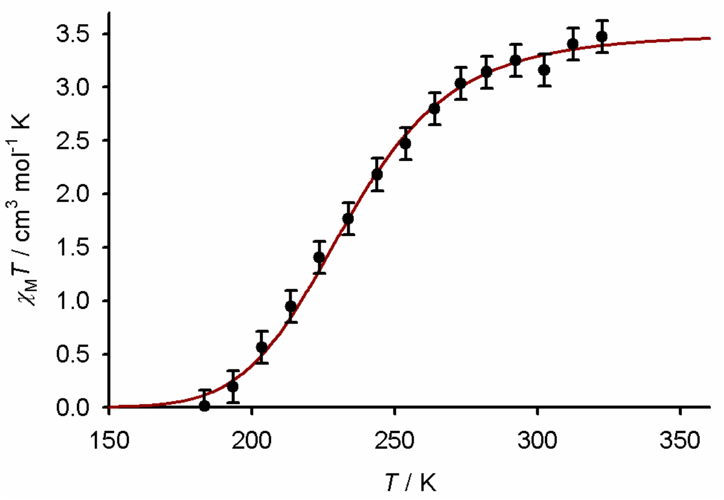


**Figure S46** Variable temperature magnetic susceptibility data for **1[BF$_4$]$_2$** in $CD_3CN$ solution (top) and **2[BF$_4$]$_2$** in $(CD_3)_2CO$ (bottom). The lines show the best fit of the data to eq 1 and 2 in the main article.

**Table S15** Fitted spin-crossover parameters for **1[BF$_4$]$_2$** and **2[BF$_4$]$_2$** in solution (eq 1 and 2).

| | Solvent | $T_{½}$ / K | $\Delta H$ / kJ mol$^{-1}$ | $\Delta S$ / J mol$^{-1}$ K$^{-1}$ |
|---|---|---|---|---|
| **1[BF$_4$]$_2$** | $CD_3CN$ | 228 ±3 | 25.1 ±1.4 | 110 ±6 |
| **2[BF$_4$]$_2$** | $(CD_3)_2CO$ | 233 ±2 | 24.0 ±1.0 | 103 ±4 |

These parameters were derived by fitting the data in Figure S46 to eq 1 and 2, where $n$HS($T$) is the high-spin fraction of the sample at temperature $T$:

$$\ln[(1- n\mathrm{HS}(T))/n\mathrm{HS}(T)] = \Delta H/RT - \Delta S/R \qquad (1)$$

$$\Delta S = \Delta H/T_{½} \qquad (2)$$

The thermodynamic parameters in the Table are typical for a complex of this type.[26]

Our previously published correlation predicts $T_{½}$ = 229 K for $[Fe(bpp^R)_2]^{2+}$ derivatives with an alkyl-disulfanyl ‘R’ substituent,[26] whose $\sigma_P$ Hammett parameter is +0.13.[27] While correlations of $T_{½}$ against the alternative $\sigma_P^+$ Hammett parameter are often more accurate,[26] $\sigma_P^+$ values for alkyldisulfanyl substituents have not been measured.[27]

## References

(1) Kershaw Cook, L. J.; Shepherd, H. J.; Comyn, T. P.; Baldé, C.; Cespedes, O.; Chastanet, G.; Halcrow, M. A. Decoupled Spin-Crossover and Structural Phase Transition in a Molecular Iron(II) Complex. *Chem. – Eur. J.* **2015**, *21*, 4805–4816.

(2) Kershaw Cook, L. J.; Fisher, J.; Harding, L. P.; Halcrow, M. A. An Iron(II) Spin-Crossover Metallacycle from a Back-To-Back Bis-[dipyrazolylpyridine]. *Dalton Trans.* **2015**, *44*, 9417–9425.

(3) Kershaw Cook, L. J.; Kulmaczewski, R.; Cespedes, O.; Halcrow, M. A. Different Spin State Behaviors in Isostructural Solvates of a Molecular Iron(II) Complex. *Chem. – Eur. J.* **2016**, *22*, 1789–1799.

(4) Sheldrick, G. M. Crystal Structure Refinement with *SHELXL*. *Acta Cryst. Sect. C: Struct. Chem.* **2015**, *71*, 3–8.

(5) Barbour, L. J. *X-Seed 4*: Updates to a Program for Small-Molecule Supramolecular Crystallography. *J. Appl. Cryst.* **2020**, *53*, 1141–1146.

(6) Dolomanov, O. V.; Bourhis, L. J.; Gildea, R. J.; Howard, J. A. K.; Puschmann, H. *OLEX2*: a Complete Structure Solution, Refinement and Analysis Program. *J. Appl. Cryst.* **2009**, *42*, 339–341.

(7) Guionneau, P.; Marchivie, M.; Bravic, G.; Létard, J.-F.; Chasseau, D. Structural Aspects of Spin Crossover. Example of the $[Fe^{II}L_n(NCS)_2]$ Complexes. *Top. Curr. Chem.* **2004**, *234*, 97–128.

(8) Capel Berdiell, I.; Kulmaczewski, R.; Halcrow, M. A. Iron(II) Complexes of 2,4-Dipyrazolyl-1,3,5-triazine Derivatives – the Influence of Ligand Geometry on Metal Ion Spin state. *Inorg. Chem.* **2017**, *56*, 8817–8828.

(9) McCusker, J. K.; Rheingold, A. L.; Hendrickson, D. N. Variable-Temperature Studies of Laser-Initiated $^5T_2 \rightarrow {}^1A_1$ Intersystem Crossing in Spin-Crossover Complexes: Empirical Correlations between Activation Parameters and Ligand Structure in a Series of Polypyridyl Ferrous Complexes. *Inorg. Chem.* **1996**, *35*, 2100–2112.

(10) Halcrow, M. A. Structure:Function Relationships in Molecular Spin-Crossover Complexes. *Chem. Soc. Rev.* **2011**, *40*, 4119–4142.

(11) Kershaw Cook, L. J.; Mohammed, R.; Sherborne, G.; Roberts, T. D.; Alvarez, S.; Halcrow, M. A. Spin State Behaviour of Iron(II)/Dipyrazolylpyridine Complexes. New Insights from Crystallographic and Solution Measurements. *Coord. Chem. Rev.* **2015**, *289–290*, 2–12.

(12) Holland, J. M.; McAllister, J. A.; Kilner, C. A.; Thornton-Pett, M.; Bridgeman, A. J.; Halcrow, M. A. Stereochemical Effects on the Spin-State Transition Shown by Salts of $[FeL_2]^{2+}$ [L = 2,6-Di(pyrazol-1-yl)pyridine]. *J. Chem. Soc., Dalton Trans.* **2002**, *2002*, 548–554.

(13) Vela, S.; Novoa, J. J.; Ribas-Arino, J. Insights into the Crystal-Packing Effects on the Spin Crossover of $[Fe^{II}(1\text{-bpp})_2]^{2+}$-Based Materials. *Phys. Chem. Chem. Phys.* **2014**, *16*, 27012–27024.

(14) Other high-spin $[Fe(bpp)_2]^{2+}$ derivatives with highly distorted coordination geometries:
(a) Elhaïk, J.; Evans, D. J.; Kilner, C. A.; Halcrow, M. A. A Structural, Magnetic and Mössbauer Spectroscopic Study of an Unusual Angular Jahn-Teller Distortion in a Series of High-Spin Iron(II) Complexes. *Dalton Trans.* **2005**, 1693–1700.
(b) Elhaïk, J.; Kilner, C. A.; Halcrow, M. A. Structural Diversity in Iron(II) Complexes of 2,6-Di(pyrazol-1-yl)pyridine and 2,6-Di(3-methylpyrazol-1-yl)pyridine. *Dalton Trans.* **2006**, 823–830.
(c) C. A.; Halcrow, M. A. An Iron(II) Complex of 2,6-Di(pyrazol-1-yl)pyrazine that Crystallises in Three Forms, Two of which Exhibit an Unusual Angular Jahn-Teller Distortion. *Polyhedron* **2006**, *25*, 235–240.
(d) Haryono, M.; Heinemann, F. W.; Petukhov, K.; Gieb, K.; Müller, P.; Grohmann, A. Parallel Crystallization of a "Static" and a Spin- Crossover Polymorph of an Iron(II) Complex from the Same Solution. *Eur. J. Inorg. Chem.* **2009**, 2136–2143.
(e) González-Prieto, R.; Fleury, B.; Schramm, F.; Zoppellaro, G.; Chandrasekar, R.; Fuhr, O.; Lebedkin, S.; Kappes, M.; Ruben, M. Tuning the Spin-Transition Properties of Pyrene-Decorated 2,6-Bis-pyrazolylpyridine Based Fe(II) Complexes. *Dalton Trans.* **2011**, *40*, 7564–7570.

(f) Hasegawa, Y.; Sakamoto, R.; Takahashi, K.; Nishihara, H. Bis[(*E*)-2,6-bis(1*H*-pyrazol-1-yl)-4-styrylpyridine]iron(II) Complex: Relationship between Thermal Spin Crossover and Crystal Solvent. *Inorg. Chem.* **2013**, *52*, 1658–1665.
(g) Pukenas, L.; Benn, F.; Lovell, E.; Santoro, A.; Kershaw Cook, L. J.; Halcrow, M. A.; Evans, S. D. Bead-Like Structures and Self-Assembled Monolayers from 2,6-Dipyrazolylpyridines and their Iron(II) Complexes. *J. Mater. Chem. C* **2015**, *3*, 7890−7896.
(h) Bridonneau, N.; Rigamonti, L.; Poneti, G.; Pinkowicz, D.; Forni, A.; Cornia, A. Evidence of Crystal Packing Effects in Stabilizing High or Low Spin States of Iron(II) Complexes with Functionalized 2,6-Bis(pyrazol-1-yl)pyridine Ligands. *Dalton Trans.* **2017**, *46*, 4075–4085.
(i) Šalitroš, I.; Herchel, R.; Fuhr, O.; González-Prieto, R.; Ruben, M. Polynuclear Iron(II) Complexes with 2,6-Bis(pyrazol-1-yl)pyridineanthracene Ligands Exhibiting Highly Distorted High-Spin Centers. *Inorg. Chem.* **2019**, *58*, 4310–4319.
(j) Capel Berdiell, I.; Kulmaczewski, R.; Warriner, S. L.; Cespedes, O.; Halcrow, M. A. Iron and Silver Complexes of 4-(Imidazol-1-yl)-2,6-di(pyrazol-1-yl)pyridine (*L*), Including a $[Fe_3(\mu\text{-}F)_2F_6L_8]^+$ Assembly. *Eur. J. Inorg. Chem.* **2020**, 4334–4340.

(15) Kershaw Cook, L. J.; Thorp-Greenwood, F. L.; Comyn, T. P.; Cespedes, O.; Chastanet, G.; Halcrow, M. A. Unexpected Spin-Crossover and a Low Pressure Phase Change in an Iron(II)/Dipyrazolylpyridine Complex Exhibiting a High-Spin Jahn-Teller Distortion. *Inorg. Chem.* **2015**, *54*, 6319–6330.

(16) Kumar, K. S.; Heinrich, B.; Vela, S.; Moreno-Pineda, E.; Bailly, C.; Ruben, M. Bi-Stable Spin-Crossover Characteristics of a Highly Distorted $[Fe(1\text{-}BPP\text{-}COOC_2H_5)_2](ClO_4)_2{\cdot}CH_3CN$ Complex**.** *Dalton Trans.* **2019**, *48*, 3825–3830.

(17) Capel Berdiell, I.; Kulmaczewski, R.; Shahid, N.; Cespedes, O.; Halcrow, M. A. The Number and Shape of Lattice Solvent Molecules Controls Spin-Crossover in an Isomorphous Series of Crystalline Solvate Salts. *Chem. Commun.* **2021**, *57*, 6566–6569.

(18) Gordon, A. J.; Ford, R. A. *The Chemists Companion – A Handbook of Practical Data, Techniques and References*, John Wiley, Chichester, 1972, p. 109.

(19) (a) Money, V. A.; Carbonera, C.; Elhaïk, J.; Halcrow, M. A.; Howard, J. A. K.; Létard, J.-F. Interplay Between Kinetically Slow Thermal Spin-Crossover and Metastable High-Spin State Relaxation in an Iron(II) Complex with Similar $T_{½}$ and *T*(LIESST). *Chem. – Eur. J.* **2007**, *13*, 5503–5514.
(b) Kulmaczewski, R.; Trzop, E.; Kershaw Cook, L. J.; Collet, E.; Chastanet, G.; Halcrow, M. A. Role of Symmetry Breaking in the Structural Trapping of Light-Induced Excited Spin States. *Chem. Commun.* **2017**, *53*, 13268–13271.
(c) Galadzhun, I.; Kulmaczewski, R.; Shahid, N.; Cespedes, O.; Howard, M. J.; Halcrow, M. A. The Flexibility of Long Chain Substituents Influences Spin-Crossover in Isomorphous Lipid Bilayer Crystals. *Chem. Commun.* **2021**, *57*, 4039–4042.

(20) Spackman, M. A.; Jayatilaka, D. Hirshfeld Surface Analysis. *CrystEngComm* **2009**, *11*, 19–32.

(21) McKinnon, J. J.; Spackman, M. A.; Mitchell, A. S. Novel Tools for Visualizing and Exploring Intermolecular Interactions in Molecular Crystals. *Acta Cryst. Sect. B: Struct. Sci.* **2004**, *60*, 627–668.

(22) McKinnon, J. J.; Jayatilaka, D.; Spackman, M. A. Towards Quantitative Analysis of Intermolecular Interactions with Hirshfeld Surfaces. *Chem. Commun.* **2007**, 3814–3816.

(23) Guionneau, P.; Marchivie, M.; Bravic, G.; Létard, J.-F.; Chasseau, D. Co(II) Molecular Complexes as a Reference for the Spin Crossover in Fe(II) Analogues. *J. Mater. Chem.* **2002**, *12*, 2546–2551.

(24) Kulmaczewski, R.; Trzop, E.; Collet, E.; Vela, S.; Halcrow, M. A. Structure:Function Relationships for Thermal and Light-Induced Spin-Crossover in Isomorphous Molecular Materials. *J. Mater. Chem. C* **2020**, *8*, 8420–8429.

(25) (a) Göbbels, D.; Meyer, G. Aufbau und Abbau von $(NH_4)[BF_4]$ und $H_3N–BF_3$. *Z. Anorg. Allg. Chem.* **2002**, *628*, 1799–1805.
(b) Koval'chuk, E. P.; Reshetnyak, O. V.; Kozlovs'ka, Z. Ye.; Błażejowski, J.; Gladyshevs'kyj, R. Ye.; Obushak, M. D. Mechanism of the Benzenediazonium Tetrafluoroborate Thermolysis in the Solid State. *Thermochim. Acta* **2006**, *444*, 1–5.

(c) Nikolova, D.; Georgiev, M. Synthesis, Thermal Investigations and Kinetic Data of $Zn(BF_4)_2 \cdot 6H_2O$. *J. Thermal Anal. Calorim.* **2009**, *95*, 319–321.

(26) Kershaw Cook, L. J.; Kulmaczewski, R.; Mohammed, R.; Dudley, S.; Barrett, S. A.; Little, M. A.; Deeth, R. J.; Halcrow, M. A. A Unified Treatment of the Relationship Between Ligand Substituents and Spin State in a Family of Iron(II) Complexes. *Angew. Chem. Int. Ed.* **2016**, *55*, 4327–4331.

(27) Hansch, C.; Leo, A.; Taft, R. W. A Survey of Hammett Substituent Constants and Resonance and Field Parameters. *Chem. Rev.* **1991**, *91*, 165–195.

.
